# Answer ALS, a large-scale resource for sporadic and familial ALS combining clinical and multi-omics data from induced pluripotent cell lines

**DOI:** 10.1038/s41593-021-01006-0

**Published:** 2022-02-03

**Authors:** Emily G. Baxi, Terri Thompson, Jonathan Li, Julia A. Kaye, Ryan G. Lim, Jie Wu, Divya Ramamoorthy, Leandro Lima, Vineet Vaibhav, Andrea Matlock, Aaron Frank, Alyssa N. Coyne, Barry Landin, Loren Ornelas, Elizabeth Mosmiller, Sara Thrower, S. Michelle Farr, Lindsey Panther, Emilda Gomez, Erick Galvez, Daniel Perez, Imara Meepe, Susan Lei, Berhan Mandefro, Hannah Trost, Louis Pinedo, Maria G. Banuelos, Chunyan Liu, Ruby Moran, Veronica Garcia, Michael Workman, Richie Ho, Stacia Wyman, Jennifer Roggenbuck, Matthew B. Harms, Jennifer Stocksdale, Ricardo Miramontes, Keona Wang, Vidya Venkatraman, Ronald Holewenski, Niveda Sundararaman, Rakhi Pandey, Danica-Mae Manalo, Aneesh Donde, Nhan Huynh, Miriam Adam, Brook T. Wassie, Edward Vertudes, Naufa Amirani, Krishna Raja, Reuben Thomas, Lindsey Hayes, Alex Lenail, Aianna Cerezo, Sarah Luppino, Alanna Farrar, Lindsay Pothier, Carolyn Prina, Todd Morgan, Arish Jamil, Sarah Heintzman, Jennifer Jockel-Balsarotti, Elizabeth Karanja, Jesse Markway, Molly McCallum, Ben Joslin, Deniz Alibazoglu, Stephen Kolb, Senda Ajroud-Driss, Robert Baloh, Daragh Heitzman, Tim Miller, Jonathan D. Glass, Natasha Leanna Patel-Murray, Hong Yu, Ervin Sinani, Prasha Vigneswaran, Alexander V. Sherman, Omar Ahmad, Promit Roy, Jay C. Beavers, Steven Zeiler, John W. Krakauer, Carla Agurto, Guillermo Cecchi, Mary Bellard, Yogindra Raghav, Karen Sachs, Tobias Ehrenberger, Elizabeth Bruce, Merit E. Cudkowicz, Nicholas Maragakis, Raquel Norel, Jennifer E. Van Eyk, Steven Finkbeiner, James Berry, Dhruv Sareen, Leslie M. Thompson, Ernest Fraenkel, Clive N. Svendsen, Jeffrey D. Rothstein

**Affiliations:** 1 Brain Science Institute, Johns Hopkins University School of Medicine, Baltimore, MD USA; 2 Department of Neurology, Johns Hopkins University School of Medicine, Baltimore, MD USA; 3 On Point Scientific Inc., San Diego, CA USA; 4 Department of Biological Engineering, Massachusetts Institute of Technology, Cambridge, MA USA; 5 Center for Systems and Therapeutics and the Taube/Koret Center for Neurodegenerative Disease, Gladstone Institutes and the Departments of Neurology and Physiology, University of California, San Francisco, San Francisco, CA USA; 6 UCI MIND, University of California, Irvine, CA USA; 7 Department of Biological Chemistry, University of California, Irvine, CA USA; 8 Advanced Clinical Biosystems Research Institute, The Barbra Streisand Heart Center, The Smidt Heart Institute, Cedars-Sinai Medical Center, Los Angeles, CA USA; 9 Cedars-Sinai Biomanufacturing Center, Cedars-Sinai Medical Center, Los Angeles, CA USA; 10 Computational Biology Center, IBM T.J. Watson Research Center, Yorktown Heights, NY USA; 11 Department of Neurology, Healey Center, Massachusetts General Hospital, Harvard Medical School, Boston, MA USA; 12 Technome LLC, Herndon, VA USA; 13 The Board of Governors Regenerative Medicine Institute, Cedars-Sinai Medical Center, Los Angeles, CA USA; 14 Zofia Consulting, Reston, VA USA; 15 Department of Neurology and Genetics, Ohio State University Wexner Medical Center, Columbus, OH USA; 16 Department of Psychiatry and Human Behavior and Sue and Bill Gross Stem Cell Center, University of California, Irvine, CA USA; 17 Texas Neurology, Dallas, TX USA; 18 Department of Neurology, Emory University, Atlanta, GA USA; 19 Department of Neurology, Washington University, St. Louis, MO USA; 20 Department of Neurology, Northwestern University, Chicago, IL USA; 21 Microsoft Research, Microsoft Corporation, Redmond, WA USA; 22 Microsoft University Relations, Microsoft Corporation, Redmond, WA USA; 23 Department of Neurobiology and Behavior, University of California, Irvine, CA USA

**Keywords:** Predictive markers, Amyotrophic lateral sclerosis, Data integration, Experimental models of disease, Stem cells

## Abstract

Answer ALS is a biological and clinical resource of patient-derived, induced pluripotent stem (iPS) cell lines, multi-omic data derived from iPS neurons and longitudinal clinical and smartphone data from over 1,000 patients with ALS. This resource provides population-level biological and clinical data that may be employed to identify clinical–molecular–biochemical subtypes of amyotrophic lateral sclerosis (ALS). A unique smartphone-based system was employed to collect deep clinical data, including fine motor activity, speech, breathing and linguistics/cognition. The iPS spinal neurons were blood derived from each patient and these cells underwent multi-omic analytics including whole-genome sequencing, RNA transcriptomics, ATAC-sequencing and proteomics. The intent of these data is for the generation of integrated clinical and biological signatures using bioinformatics, statistics and computational biology to establish patterns that may lead to a better understanding of the underlying mechanisms of disease, including subgroup identification. A web portal for open-source sharing of all data was developed for widespread community-based data analytics.

## Abstract (web summary)

Answer ALS is a resource of patient-derived iPS cell lines, multi-omic data derived from iPS neurons and longitudinal clinical and smartphone data from over 1,000 patients with ALS. This serves as a foundation to identify distinct disease subgroups.

## Main

Over the last several decades, tremendous progress in the optimization of therapies for various medical conditions, such as cancer, has been realized. Many factors underlie this therapeutic success, including optimization of clinical trial design, new pathway-specific pharmaceuticals and the coordination of participant recruitment efforts across clinics. Perhaps one of the most powerful and fundamental reasons for the success of some cancer therapies is the ability to sample diseased tissues and thereby distinguish the biological and molecular events responsible for individual diseases or disease subgroups within a disease cluster^1^. Thus, skin, breast or prostate biopsies have been important starting points for the investigation of various types of melanomas and breast or prostate cancers. Neurodegenerative diseases such as ALS, Alzheimer’s disease and Huntington’s disease have, however, not seen such advances. Clinical trials in humans, often based on findings from nonhuman model systems, have repeatedly proven disappointing^2,3^. Although there are probably many reasons for such failures (for example, poor pharmacokinetics, wrong biological pathway, lack of target engagement), a critical reason is the inability to identify disease pathways in patient tissues and to segment patients for clinical trials according to these pathways. As a result of the high risk of disability, brain and spinal cord biopsies for tissue analysis are not feasible in neurodegenerative diseases and therefore, unlike the biopsy of other organs and tissues, obtaining neural tissue during the disease course is a significant hurdle to effective therapeutic development.

An alternative is to use stem cell technology and infer disease pathways from cell lines derived from the patients’ own blood. Evidence for this approach is beginning to emerge. Early work employing iPS spinal neurons from patients with *C9orf72* ALS/frontotemporal dementia led the way to the development of the first antisense-based gene therapy for this common familial form of ALS (fALS), with an international clinical trial already under way (clinicaltrials.gov: NCT03626012)^4,5^. But for most patients with ALS, who have sporadic disease (sALS), these discoveries have yet to translate into meaningful therapies. A major barrier has been the lack of a predictive preclinical human model for sALS. However, with advances in iPS cell technology and the unprecedented data and specimen collection efforts of Answer ALS, we can now take an iPS cell-based approach to unraveling mechanisms that may cause or contribute to the heterogeneous clinical spectra of sALS, such as pattern and speed of spread and certain nonmotor manifestations. Notably, multiple gene mutations are already known to cause fALS and represent quite diverse pathways: RNA metabolism, nuclear transport, protein aggregation, axonal trafficking, glial dysfunction, etc.^6^. Curiously, the variability in clinical features is nearly as great when comparing patients with any single mutated gene as it is when comparing across genes or with sALS. Little is known about the derangements in specific biological pathway(s) driving sALS or whether there are ALS subgroups defined by specific biological derangements. Knowledge of these biological subgroups may be critically important and the success of disease-modifying therapies may depend on treating the right ‘subgroup’ with the proper pathway-targeting drug.

The Answer ALS (AALS) program was conceived as a program to generate iPS cell lines from a large number of patients with ALS and apply well-established molecular, biochemical and imaging techniques to understand the heterogeneity of sALS in these patient-derived spinal neurons, to serve as a ‘biopsy-like’ equivalent. After ensuring that results were reproducible, we assembled comprehensive biological datasets from individual subject iPS cell lines and combined them with the longitudinal clinical data. In contrast to smaller previous iPS cell experiments, studies of iPS cells from a large population, like AALS, provide the first opportunity to explore biologically relevant subgroups of sALS. This resource program was designed with the core goals of providing large clinical and biological datasets in an open source-like application that affords researchers the proper tools to identify biological subgroups and an extensive collection of IPS cell lines with which to test ALS therapies and hypotheses about ALS pathogenesis.

## Results

### Clinical demographics and clinical data generation

#### Population demographics

**Fig. 1:**
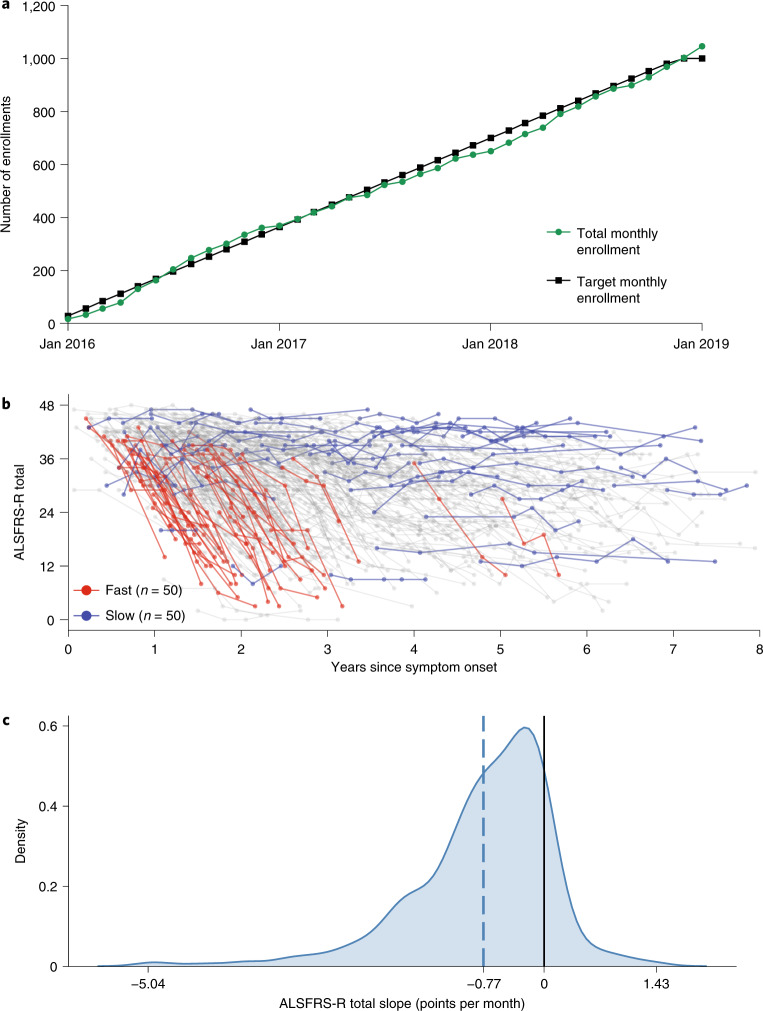
Clinical enrollment and characteristics: ALSFRS-R progression curves for all AALS clinic-enrolled subjects over a 40-month period. **a**, Patients with AALS and control subject enrollment. **b**, ALSFRS-R total slope distribution. Kernel density estimation with Gaussian kernels was used to estimate the probability density function of the ALSFRS-R slope. The dashed line indicates the mean ALSFRS-R slope. **c**, Longitudinal ALSFRS-R measurements with fast and slow progressors. Participants with three or more visits and a maximum visit dates within 8 years of symptom onset were included. The number of participants in fast and slow progressing groups, sorted by ALSFRS-R slope, is indicated by *n*.

**Extended Data Fig. 1:**
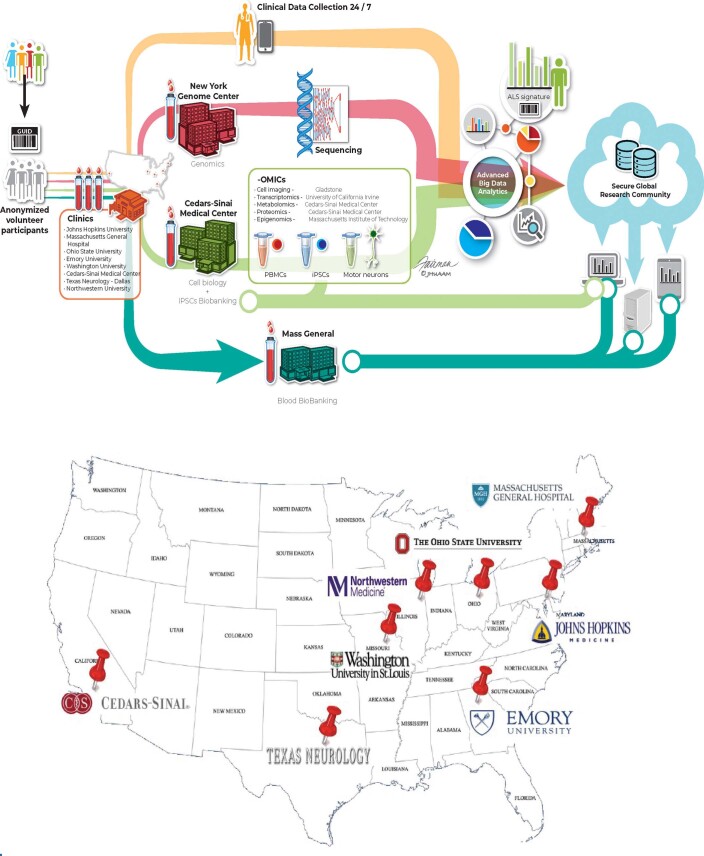
Answer ALS Operations. **Top. Answer ALS Research Program**. Graphic illustration of overall program flow. **Bottom. Clinical Sites**. Participating clinics were districted nationally at 8 academic or private neurology clinics specializing in ALS clinical care and research.

The enrolled participant population for the AALS program (Fig. 1a, Extended Data Fig. 1, Supplementary Information and Supplementary Tables 1–5) had clinical characteristics comparable to past large sALS population demographics, with a slightly higher number of male than female participants, site of disease onset predominantly a limb rather than bulbar and a mean age of disease onset of approximately 57 years. The mean delay in clinical diagnosis for **ALS patients** included in the study was 14.8 months. A higher percentage of patients with rapid progression had bulbar-onset disease. There was a wide range of disease progression rates over the time period of observation (Fig. 1b,c), with an average follow-up duration of 12.5 months and an average rate of decline of 0.77 points per month (Fig. 1b,c). The smaller population of patients with fALS in the resource had typical representations of the common gene mutations including *C9orf72* and *SOD1* (Table 1), with a small subset of patients with *C9orf72* and non-*C9orf72* ALSs developing cognitive decline during the study (https://dataportal.AnswerALS.org). A small number of individuals were ALS mutation carriers (asymptomatic ALS) without overt neurological disease (Table 1). Non-ALS motor neuron disease (MND) included patients with predominantly upper MND, not formally categorized as ALS (for example, primary lateral sclerosis), and their demographic information is included in Supplementary Table 4. The healthy control subject population consisted of age-matched participants without ALS or a family history of ALS.

**Table 1:** Answer ALS basic clinical demographics

			Subjects				Statistics
Variable	Level	Overall: no. (%)	ALS: no. (%)	Asymptomatic ALSl: no. (%)	Healthy control: no. (%)	Non-ALS MND: no. (%)	ALS versus healthy control
Participants	*n*	100.0 (1,047)	82.2 (861)	1.1 (12)	10.3 (108)	6.3 (66)	
Sex	Female	40.6 (423)	37.4 (320)	58.3 (7)	66.4 (71)	37.9 (25)	<0.001
	Male	59.4% (618)	62.6 (536)	41.7 (5)	33.6 (36)	62.1 (41)	<0.001
	[missing]	(6)	(5)	(0)	(1)	(0)	N/A
Race	Native American	0.2 (2)	0.1 (1)	0.0 (0)	1.0 (1)	0.0 (0)	0.078
	Asian	2.0 (21)	1.5 (13)	0.0 (0)	5.7 (6)	3.0 (2)	0.004
	Black	4.8 (49)	5.0 (42)	0.0 (0)	4.8 (5)	3.0 (2)	0.928
	Pacific Islander	0.1 (1)	0.1 (1)	0.0 (0)	0.0 (0)	0.0 (0)	0.724
	White	92.9 (956)	93.3 (789)	100.0 (12)	88.6 (93)	93.9 (62)	0.081
	[missing]	(18)	(15)	(0)	(3)	(0)	N/A
Ethnicity	Hispanic or Latino	4.8 (50)	5.3 (45)	0.0 (0)	2.8 (3)	3.1 (2)	0.271
	Not Hispanic or Latino	95.2 (989)	94.7 (810)	100.0 (12)	97.2 (104)	96.9 (63)	0.271
	[missing]	(8)	(6)	(0)	(1)	(1)	N/A
Age at baseline (years)	Mean (s.d.)	58.9 ± 11.6 (20.0, 91.0)	59.3 ± 11.1 (24.0, 91.0)	48.3 ± 10.3 (33.0, 62.0)	55.0 ± 14.1 (20.0, 82.0)	61.9 ± 12.0 (26.0, 85.0)	<0.001
Time between symptom onset and diagnosis (months)	Mean (s.d.)	15.9 ± 20.4 (−5.7, 286)	14.8 ± 16.8 (−5.7, 185)	N/A	N/A	40.8 ± 52.6 (0.1, 286)	N/A
Time between symptom onset and study enrollment (months)	Mean (s.d.)	32.0 ± 39.4 (0.6, 458)	29.8 ± 35.6 (0.6, 458)	N/A	N/A	78.4 ± 75.3 (11.1, 353)	N/A
BMI at screening visit	Mean (s.d.)	26.8 ± 6.39 (10.1, 150)	26.5 ± 4.83 (10.1, 44.4)	29.2 ± 3.38 (23.6, 34.2)	29.2 ± 14.9 (17.0, 150)	27.3 ± 5.61 (16.6, 47.3)	<0.001
ALSFRS-R at first ALSFRS-R visit	Mean (s.d.)	33.8 ± 8.65 (0.0, 47.0)	33.8 ± 8.67 (0.0, 47.0)	N/A	N/A	33.5 ± 8.44 (7.0, 46.0)	N/A
ALSFRS-R slope		−0.73 ± 0.87 (−5.1, 1.4)	−0.77 ± 0.88 (−5.1, 1.4)	N/A	N/A	−0.11 ± 0.40 (−1.6, 1.0)	N/A
FVC (percentage predicted) at first ALSFRS-R visit	Mean (s.d.)	69.9 ± 24.0 (4.0, 126)	69.6 ± 23.9 (4.0, 125)	N/A	N/A	73.7 ± 25.3 (17.0, 126)	N/A
FVC slope		−1.5 ± 2.53 (−16,14.1)	−1.6 ± 2.59 (−16,14.1)	N/A	N/A	−0.12 ± 0.86 (−1.9, 2.1)	N/A
Follow-up duration	Months (mean (s.d.))	13.3 ± 17.3 (0.0, 340)	12.5 ± 12.6 (0.0, 94.1)	N/A	N/A	24.0 ± 47.2 (0.0, 340)	N/A
Time from onset to death	Months (mean (s.d.))	N/A	34.7 ± 27.6 (8.3, 187)	N/A	N/A	N/A	N/A

BMI, body mass index; N/A, not available.

#### App-based voice recordings—motor and speech analyses

**Fig. 2:**
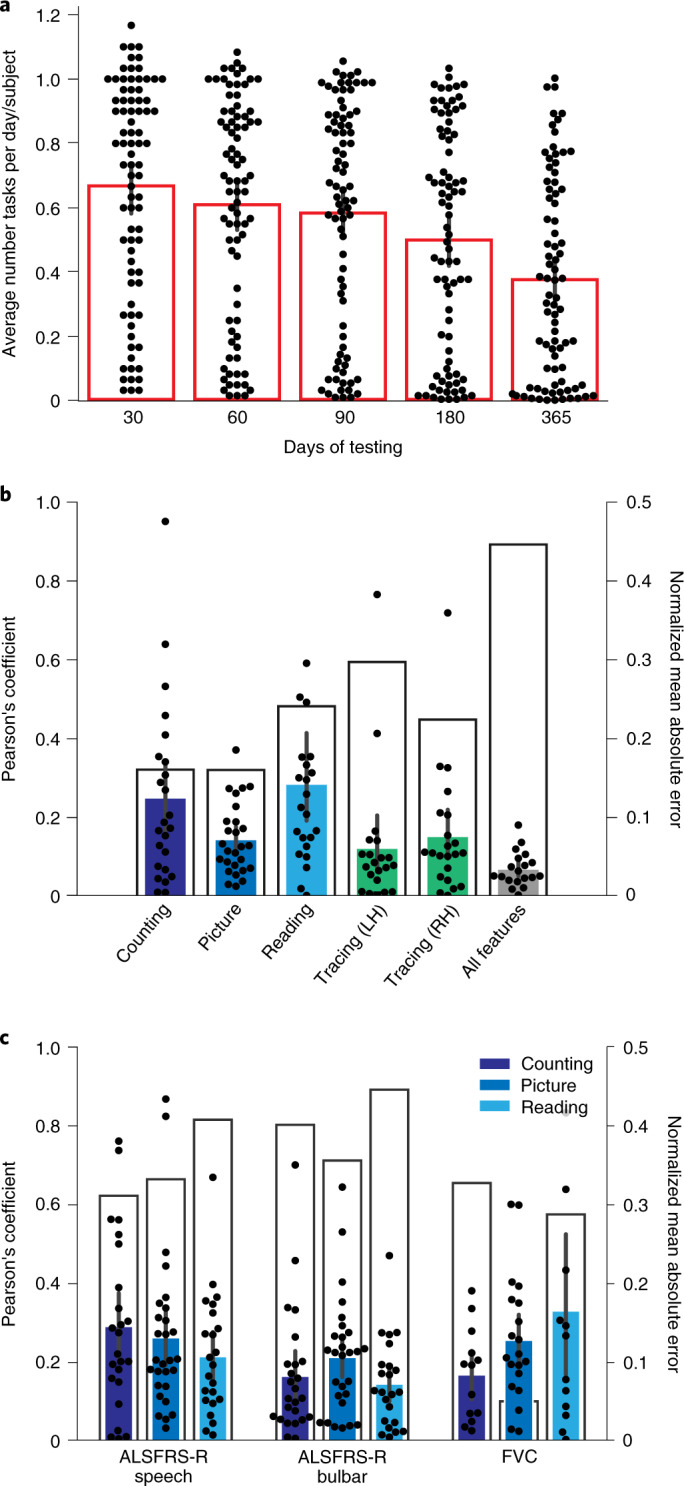
Smartphone use and analytics (*n* = 80 biologically independent samples). **a**, Smartphone app compliance mean and 95% confidence interval (CI). Compliance was calculated using the average number of tasks done per day and per subject. **b**, Results of inferring ALSFRS-R total. Pearson’s values are shown in black contoured bars (left, *y* axis) and mean absolute errors of the prediction are shown in color bars with 95% CI (right, *y* axis). Performance values were obtained using each individual task as well as the combination of all the tasks. The highest performance was obtained using all tasks (*R* = 0.89, *P* < 1 × 10^−5^). LH, left hand; RH, right hand. **c**, Results of inferring ALSFRS-R scores using only speech-related tasks. Pearson’s values are shown in black contoured bars (left, *y* axis) and the mean absolute errors of the prediction are shown in color bars with 95% CI (right, *y* axis). Performance values were calculated independently for each of the three speech tasks to infer FVC and ALSFRS-R speech and bulbar subscores. Highest performance was obtained using information from the reading task for both ALSFRS-R subscores, obtaining up to *R* = 0.89 (*P* < 1 × 10^−5^) or ALSFRS-R bulbar subscore. On the other hand, counting task information produced the best result when inferring the FVC score (*R* = 0.65, *P* = 2 × 10^−2^).

A core tool to gather more comprehensive longitudinal clinical data, ultimately to integrate with the biological datasets, was the development of a new smartphone app, designed to inform elements of motor activity, speech, breathing, voice and cognition (Supplementary Information) while patients were at home. Given the nature of this progressively disabling disorder, the reliability of utilization is an important variable. Compliance for using the smartphone app was analyzed over 18 months from the beginning of the app rollout to a subset of 80 study subjects. Surprisingly, only a modest decrease in compliance was observed with increased duration of use (Fig. 2a).

#### App data accurately predicted clinical progression

**Extended Data Fig. 2:**
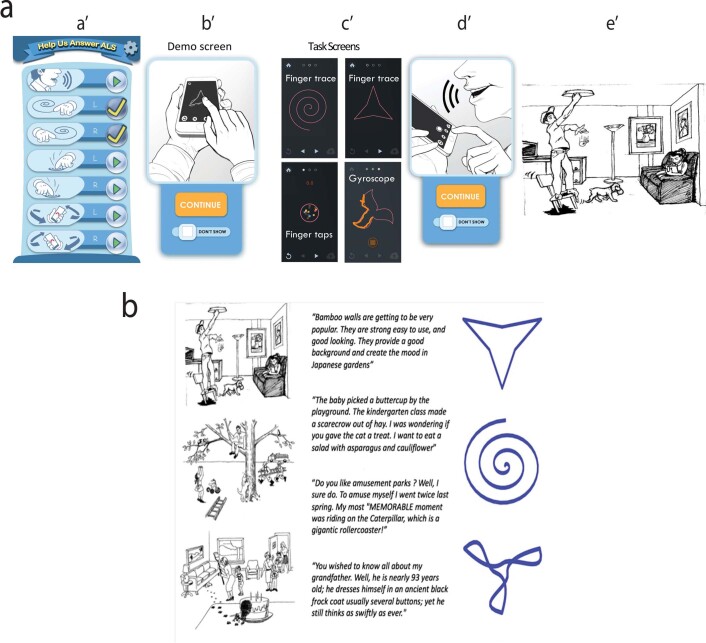
Smartphone App. **a. Smartphone App**. Illustrations from app of various activities. a’. Main Menu, b’. Upper limb motor tests, c’. Bulbar activities, including single breath counting, speech and cognition, d’. Example of cartoon used for speech/cognition analytics. **b. Examples of speech and fine motor tasks performed by the smartphone app study participants**. Data are collected with an app called “Help us Answer ALS”. Each week, the app asks the participant to perform different tasks. The tasks involve motor control in the upper body, speech and cognition. Each task is performed once per week. The speech tasks include describing a picture (a,b,c), reading a passage (d,e,f), and counting until the subject runs out of breath (not represented). Describing a picture also serves as a cognition task. The motor task involves tracing 3 different contours in sequential order (h,i,j), alternating hand each day of the week.

From speech recordings, we extracted linguistic features to evaluate word diversity and complexity of thought such as semantic similarity, dispersion and frequency, as recently detailed^7^. Features derived from the voice tasks (single-breath count, read-aloud passage and free speech; Extended Data Fig. 2) each correlated highly with the bulbar subdomain of the ALS Functional Rating Scale-Revised (ALSFRS-R; Pearson’s *R* = 0.8, slope = 1.14; Pearson’s *R* = 0.89, slope = 0.98; and Pearson’s *R* = 0.71, slope = 1.12, respectively). Features from the finger tracing showed modest individual correlations with the ALSFRS-R total score (Fig. 2b and Extended Data Fig. 2). Importantly, the combination of features from all of these tasks correlated very highly with the ALSFRS-R total score (Pearson’s *R* = 0.89, slope = 1.16; Fig. 2c).

Features obtained from the single-breath counting task correlated well with vital capacity (*R* = 0.63) and strongly suggest that voice analysis could be a proxy for vital capacity measurements in a clinic. Similar results by others employing sustained phonation are in agreement with our new observations^8^.

Importantly, semantic analysis of the picture description task was highly correlated with the ALS-Cognitive Behavioral Screen (CBS) (*R* = 0.72) and less correlated with the central nervous system (CNS) lability scale (*R* = 0.45). These studies then also suggest that at-home app analytics can be useful for longitudinal cognition analytics.

This task also predicted well the ALSFRS-R speech subscore (Fig. 2b); however, models using features from the reading task outperformed the counting and picture description tasks. A more detailed account of these results is reported elsewhere^7^.

These results demonstrate that the modules implemented to assess hand function and speech may be useful to quantify ALS function when patients are not in clinic and can substantially aid in the acquisition of progressively declining clinical indices. Furthermore, the picture description task may be useful to evaluate cognitive function in ALS. The potential to record voice and store it encrypted in the cloud could provide a powerful clinical tool to assess change over time that could be used clinically and in ALS trials.

### Production of the iPS cell line

**Fig. 3:**
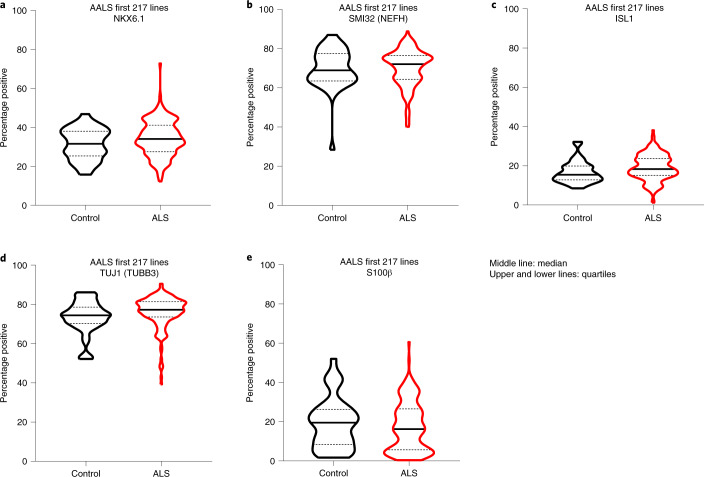
Uniformity in the generation of large sets of ALS and control iPS cell lines. Violin plots of immunofluorescent immunocytochemistry-stained diMNs cultures quantified using Image Express Micro. The iPS cells from both control and patients with ALS differentiated for 32 d after the Cedars-Sinai Biomanufacturing Center-directed diMN protocol, then fixed, immunostained and analyzed for the number of cells that stain positively for neuronal (**a**: NKX6.1, **b**: SMI32 (NEFH), **c**: ISL1, **d**: TUJ1 (TUBB3)) and non-neuronal marker proteins (**e**: s100β). Data are presented as a positive percentage of total DAPI-labeled nuclei; 217 different subject iPS cell lines were analyzed. There were no significant differences between ALS and control for any of the assessments.

**Extended Data Fig. 3:**
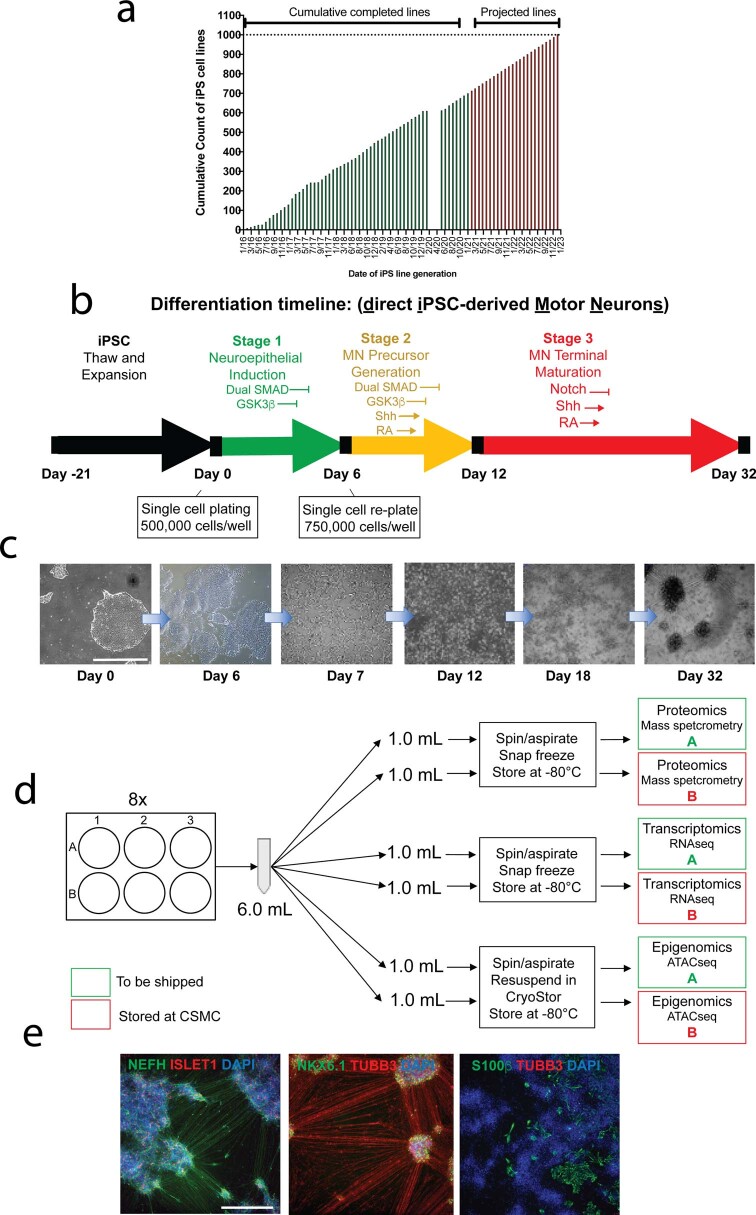
Production of ALS and control iPS cell spinal motor neurons. **a. Example of IPS Generation Schedule. b**. Method of generating iPS cell-derived motor neuron cell lines using the diMNs protocol. **c**. Brightfield images show the morphology of the cells during differentiation from iPS cell stage to the generation of motor neurons over a period of 32 days. **d**. Production flow and harvesting schematic of diMNs for multi-omics analyses. **e**. Quality control of the diMNs produced from iPS cells is performed by imaging of representative wells for immunohistochemical staining with neuronal, motor neuron and glial markers after 32 days of differentiation. Scale bar=400μm. Images representative of over 600 patient cell lines.

A core design and strength of the program are the set of iPS cell lines from a large population of >1,000 patients with ALS and control subjects, all deeply phenotyped, provided to the research community. To date, more than 850 of the iPS cell lines have been generated and are available through the web portal. Out of the ~850 unique samples, only 18 lines (~2%) failed reprogramming. As there are multiple different protocols to generate iPS cells and differentiate them into motor neurons, it was essential that the uniformity of the generated cultures be evaluated, thereby establishing the reliability of this new and renewable biological resource. To address this central issue, we evaluated the iPS cell-derived spinal neurons from a large cohort of 217 control and ALS iPS cell lines. Specifically, we examined expression of five different cell-identifying markers for neurons and glia, including cell markers NKX6.1, SMI32, ISL1, TUJ1 and S100beta. This differentiation protocol (Extended Data Fig. 3) generates a mixed population of neurons consisting of ~75% (±8%) β_III_-tubulin- (TuJ1-) and ~70% (±10%) NF-H-positive cells, ~19% (±6%) Islet-1- and ~34% (±9%) Nkx6.1-positive spinal motor neurons, and ~18% (+/13%) S100B-positive progenitors 32 d after the onset of differentiation (Fig. 3 and Supplementary Table 6). As shown in Fig. 3, there was great uniformity in the cellular composition of the cultures for this large selection of human lines. This was important, because past work or methods can lead to variable cultures, making the interpretation of downstream analysis complicated. Notably the cellular composition was not substantially different between the ALS and control iPS cell-derived neurons. As expected, these cultures presented a mixture of motor neurons, neurons and, to a lesser extent, glia. This was important, because ALS is not simply a motor neuron disease, but is a disorder of multiple different nervous system cell types, as reflected in these uniformly generated cultures.

### Generation of multi-omics data

#### Genomics

**Fig. 4:**
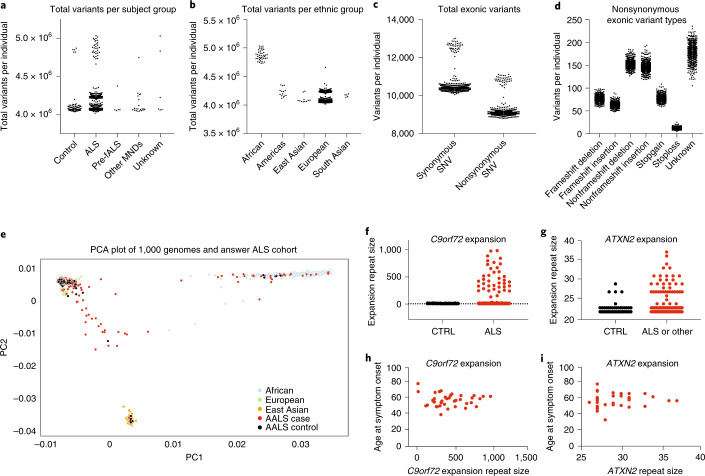
Summary of variants for the AALS cohort of 830 sequences. **a,** Total number of variants per participant. **b,** Total variants per participant based on ethnic origin. DHS, DNase 1-hypersensitive site**. c**, Total exonic variants**. d**, Nonsynonymous variant types. Each dot represents a participant. **e**, PCA plot revealing how the AALS samples cluster among various ancestry groups of the 1000 Genomes Project dataset. PC1 showed that African samples (green) clustered apart from the other populations and PC2 that Asian samples (red/brown) were distinct from European samples (purple), with admixed American located in between. Most of the AALS samples were clustered with the European samples, although some were closer to the African group and a few clustered with the Asian group, corroborating the NYGC ancestry results (**b**). **f**,**g**, Size of the repeat expansion in *C9orf72* (**f**) and *ATXN2* (**g**) for the AALS cohort. The graphs are based on Expansion Hunter^14^ reads for 601 sequences out of the AALS 830 samples. Top: 41 ALS cases and 4 individuals who are pre-fALS have expansions >26 repeats. Bottom: 35 ALS cases have *ATXN2* expansions, whereas 4 normal controls and 1 uncharacterized individual have *ATXN2* expansions >26 repeats. CTRL, controls. **h**,**i**, The **r**elationship between repeat size in *C9orf72* (**h**) or *ATXN2* (**i**) and age of ALS onset (*n* = 830 biologically independent samples). Data are presented as mean values ± s.e.m.

As an appreciation of the overall diversity of the program’s ALS and control population, especially valuable for future global analytics, we evaluated the AALS cohort using New York Genome Center’s (NYGC’s) ancestry pipeline^9^. Most participants were white and of European descent (91.45%); the remainder had ancestry consistent with the Americas (1.69%), Africa (4.94%) and east (1.33%) and south Asia (0.6%) (Fig. 4). On average, each sample harbored a total of ~4.1 million variants and ~9,800 protein-altering variants, including SNPs, frameshift and nonframeshift deletions and insertions, and protein-truncating variants (Table 2 and Fig. 4a–d), similar to previous reports^10^. Notably, the samples with African descent had a higher number of variants than other ethnic populations, as expected (Fig. 4b)^11^.

**Table 2:** Summary table of variants in the AALS cohort

Variant type	Total variants in all genes in ALS cases	Total variants in all genes in CTRLs	ALS gene^a^ variants in ALS	ALS gene^a^ variants in controls	Number of variants per 33-ALS gene
All variants	Sum = 2,941,489,030 Average = 4,166,415 variants per ALS case	Sum = 379,092,863 Average = 4,120,575 variants per control	Sum = 1,092 Average = 1.5 variants per ALS case	Sum = 141 Average = 1.5 variants per control	*ALS2* (20), *ANG* (5), *ANXA11* (15), *ATXN2* (29), *C21orf2* (19), *C9orf72* (5), *CAMTA1* (24), *CCNF* (28), *CHCHD10* (2), *DAO* (7), *DCTN1* (24), *FIG4* (14), *FUS* (6), *HNRNPA1* (2), *HNRNPA2B1* (1), *KIF5A* (9), *MATR3* (10), *MOBP* (4), *NEK1* (19), *OPTN* (10), *PFN1* (7), *SCFD1* (13), *SETX* (57), *SOD1* (14), *SQSTM1* (14), *TAF15* (16), *TARDBP* (11), *TBK1* (18), *TUBA4A* (3), *UBQLN2* (7), *UNC13A* (19), *VAPB* (4), *VCP* (4). Details of variants are found in Supplementary Tables
ClinVar P/LP (C-PLP) variants	Sum = 23,924 Average = 33.9 variants per ALS case Rare = 3,659 (5.2 variants per ALS case)	Sum = 3,097 Average = 33.7 variants per control Rare = 61 (5 variants per control)	Sum = 85 (2% of cases harbor) Rare only = 21 (3% of cases harbor)	Sum = 11 (12% of controls harbor) Rare only = 3 (3.3% or control harbor)	*ALS2* (1) *ANG* (2), *CHCHD10* (1), *FIG4* (2), *FUS* (1), *OPTN* (2)^b^, *PFN1* (2), *SETX* (4), *SOD1* (5), *SQSTM1* (3),*TARDBP* (2), *UBQLN2* (2), *VCP* (1)
Harms P/LP (H-PLP) variants	N/A	N/A	Sum = 4 (3.4% of cases harbor)	Sum = 1 (1% of controls harbor)	*FUS* (1), *PFN1* (2), *SOD1* (11), *TARDBP* (3) *UBQLN2* (1), *VCP* (1)
Intervar P/LP (I-PLP) variants	Sum = 2,346 Average = 3.3 variants per sample Rare = 2272 Average = 3.21 variants per case	Sum = 288 Average = 3.1 variants per sample Rare = 276 Average = 3.2 per control	Sum = 25 (3.5% of cases harbor)	0 (0%)	*NEK1* (2), *OPTN* (1), *SOD1* (12), *SETX* (1), *TBK1* (2), *VCP* (2),
In silico prediction: 6/9 predicted to be damaging	Sum = 79,010 Average = 112 variants per sample Rare = 40,910 Average = 58 variants per sample	Sum = 5,464 Average = 113 variants per sample Rare = 5,464 Average = 59.4 variants per sample	Sum= 97 (13.7% of cases harbor)	Sum=11 (12% of controls harbor)	*ALS2*(2), *ANXA11* (4), *ATXN2* (3), *C21orf2* (1), *CAMTA1* (1), *DAO* (3), *DCTN1* (5), *FIG4* (3), *FUS* (1), *HNRNPA2B1* (1), *KIF5A* (2)MOBP(1), NEK1(2), *OPTN* (1), *PFN1* (3), *SCFD1* (2), *SETX* (14), *SOD1* (11), *SQSTM1* (2), *TARDBP* (4), *TUBA4A* (2), *UBQLN2* (1), *UNC13A* (3), *VCP* (2)

Sum = the total number of variants found per group, ALS versus control.

^a^Variants <1%.

^b^*OPTN* variants listed here are high frequency, >1%.

We used PCA^12,13^ to visualize the ancestry background of the AALS cohort and a set of 2,504 samples from the 1000 Genomes Project with well-defined ancestry. We find that most of the samples clustered with the NYGC’s European samples, although some were closer to the African group and a few clustered with the Asian group (Fig. 4e), corroborating the NYGC ancestry results and probably consistent with the local recruiting clinics geographic locations (Extended Data Fig. 1).

#### Variants in ALS genes

As most of the ALS lines were derived from patients with sALS, an analysis of the genomic variants is important, especially as future opportunities for researchers to correlate the observed variants along with the deep clinical and multi-omics data, as well as the future use of the living cell lines. Within the 830 samples, we observed 440 exonic variants in the 33-ALS genes (Supplementary Information) that were <1% frequent (Fig. 4c,d, Table 2 and Supplementary Table 7). Both controls and ALS cases averaged 1.5 rare ALS variants per individual within the 33-ALS genes. Of these, 79% were SNPs, 13% uncharacterized, ~1% splicing, ~1% nonframeshift deletion, 1% frameshift deletion, 1% frameshift insertion, 2% frameshift insertion, 2% nonframeshift insertion and 1% stop-gain (Supplementary Table 7).

As future biological pathways in ALS subgroups could reflect the expression of genetic variants of established ALS genes, we first evaluated how many pathogenic or probably pathogenic variants existed as reported in ClinVar (CP) in the 33-ALS genes. We found that 12% of ALS cases harbored a CP variant within one of the 33-ALS genes (Supplementary Tables 7 and 8). All of these CP variants were rare (<1% frequency within the population) except two found within the *OPTN* gene. For example, we observed five *SOD1* CP variants (within eight patients with ALS), two *TDP43* CP variants (within two patients with ALS) and one CP *FUS* variant in a patient with ALS (Supplementary Tables 7 and 8). CP variants were also detected in individuals who did not show signs of ALS at the time of the clinic visit, and there were eleven CP variants within control samples (within *ALS2*, *SETX*, *OPTN* and *PFN1*), four CP variants in the pre-fALS cohort (within *FIG4*, *OPTN* and *CHCHD10*), three CP variants within individuals with other MNDs (within *SQSTM1*, *OPTN* and *PFN1*) and three CP variants in uncharacterized individuals (within *SQSTM1* and *SETX*; Supplementary Table 8). In summary, rare CP variants were observed in 3.11% (22 total) of ALS cases and 1% of controls (1 out of 92 samples). We also investigated the number of P/LP variants called by Intervar (IP), in in silico prediction (ISD variants) and a new combination of ACMG gene criteria as well as the in silico prediction and family-based segregation data, a list of high-confidence causal variants in 12 genes—*ALS2*, *CCNF*, *CHCHD10*, *FUS*, *OPTN*, *PFN1*, *SOD1*, *TARDBP*, *TBK1*, *UBQLN2*, *VAPB* and *VCP*—which have been curated and designated as the HP (Harms P/LP, Supplementary Table 7) variants. These are reported in Supplementary Tables 7–11. We investigated CP, IP and ISD variants found across all genes in 830 samples and these are listed in Supplementary Tables 12, 13 and 14.

#### Expansions in *C9orf72* and *ATXN2*

Genomic expansions of both *C9orf72* and *ataxin* 2 are associated with both fALS and sALS. The availability of large numbers of iPS cell lines and the matched multi-omics data from this phenotypically variable genetic subgroup provide a unique future opportunity to investigate these genes that alternatively lead to ALS and/or FTD. Using Expansion Hunter to identify repeat expansions within whole-genome sequencing (WGS) data, we found 601 expanded regions in the 830 samples^14^. In total, 41 patients with ALS and 4 pre-fALS subjects in the AALS study population harbored hexanucleotide expansions in *C9orf72* that were >26 repeats (Fig. 4f and Supplementary Table 15). We also observed 35 patients with ALS, 4 controls and 1 uncharacterized individual harboring CAG triplet repeat expansions in *ATXN2* >26 repeats (Fig. 4g and Supplementary Table 16). All patients with ALS with >26 *ATXN2* repeats had clinical phenotype characteristics of MNDs and no other reported neurological abnormalities. Notably, in this population of patients and cell lines, for carriers of expansions in both *ATXN2* and *C9orf72* simultaneously, we found no correlation between age of ALS onset and expansion size (Fig. 4h,i and Supplementary Tables 15). However, future multi-omic studies of the patient iPS spinal neurons may reveal different biological pathways/properties when both mutations are co-expressed in humans.

#### ACMG genes

Pathogenic or probable pathogenic variants in 59 genes are currently considered to be medically actionable by the American College of Medical Genetics and Genomics (ACMG), due to the potential for medical intervention to modify morbidity and mortality in carriers of such variants^15^. Within the 830 samples, we identified 73 C-PLP variants within 32 ACMG genes (Supplementary Table 17). Of the individuals, 50.4% did not harbor a C-PLP variant in an ACMG gene, 41.2% harbored 1, 7.6% harbored 2 and 0.84% harbored 3 C-PLP variants. Of these variants found within 110 individuals, 66 were rare (<1%; Supplementary Table 17). We also found 42 I-PLP variants within ACMG genes within 51 individuals, all of which were rare (Supplementary Table 18). Participants were offered to receive the results of these medically actionable genes through the return of genetic results substudy (Extended methods).

### Transcriptomics

**Extended Data Fig. 4:**
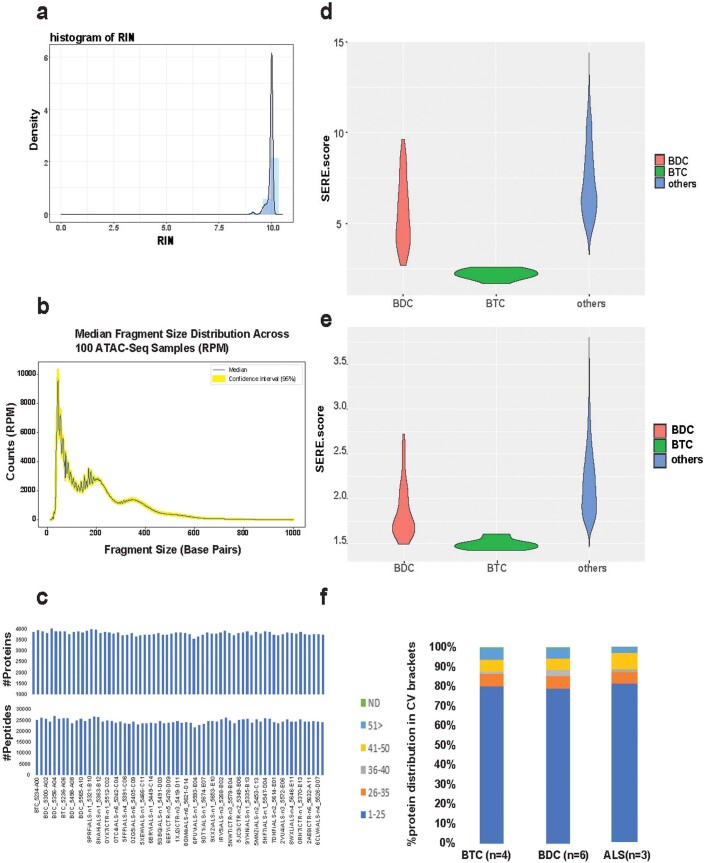
Omics Quality Control metrics. **a. Histogram of RNA integrity numbers for current AALS samples**. Density plot and histogram of RIN values for all current AALS samples with RNAseq data. Plot shows all processed samples have RIN > 8. **b. fragment size distribution** Size distribution of ATAC seq data, with peaks representing different n-nucleosomal fragments and clear nucleosome-free regions separated by ~147 bp, the size of a nucleosome. **c**. Number of Proteins and peptide identification consistency in the data generation batches of AALS samples. **d. Violin plot of SERE values for RNAseq data for current AALS samples**. Violin plot showing variance of SERE values in BTC (green) and BDC (red) control samples relative to all other (blue) current AALS samples. BTC shows lowest score with the least amount of variance indicating that samples are true technical replicates, while BDC and other samples show increase variance. **e. Violin plot of SERE values for ATACseq data for current AALS samples**. Similar to RNA data the BTC (green) show lowest variability indicating low technical confounds. **f**. Coefficient of Variation (CV) for Batch Technical Control (BTC) and Batch differentiation control (BDC) replicates showing 80% proteins to be under a CV of 25%.

**Extended Data Fig. 5:**
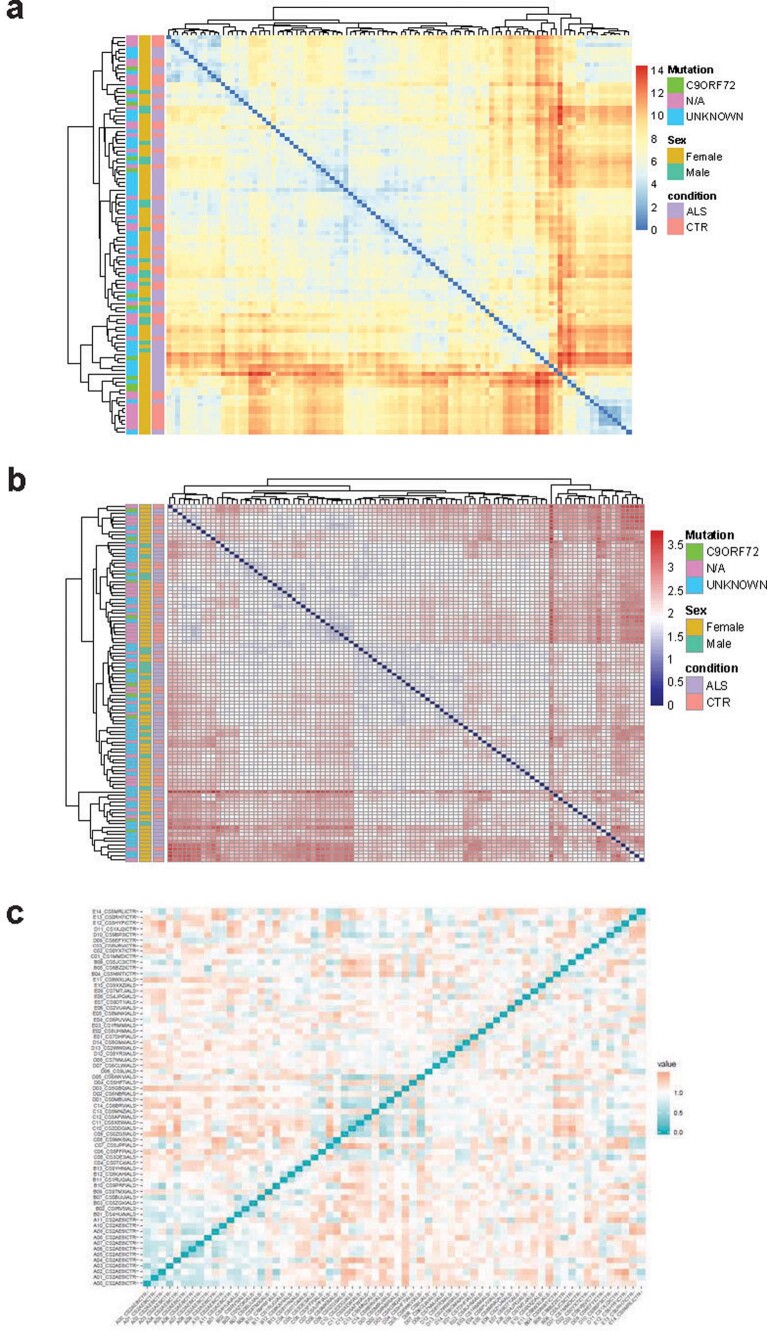
Heatmap and hierarchical clustering of current AALS samples. **a&b**. Heatmap and hierarchical clustering of SERE values using RNA/ATACseq data. Heatmap and clustering of current AALS samples using SERE values from the **(a)** RNAseq and **(b)** ATACseq data. Samples are annotated with gender, genotype, and C9orf72 mutation. No distinct clustering separates samples by these categories, but BTC sample cluster together. **c**. Spearman correlation matrix plot for the AALS proteomics data.

For each of the omics assays, vials from an identical pool of differentiated motor neurons were processed to ensure comparability, including batch differentiation controls (BDCs) and batch technical controls (BTCs) from the control 2AE8 line, as detailed in Extended methods. Overall the analytics revealed minimal to no technical confounders and low batch effects between differentiation and no clear batch-related abnormalities with regard to disease status (Extended Data Figs. 4a,d and 5a).

**Fig. 5:**
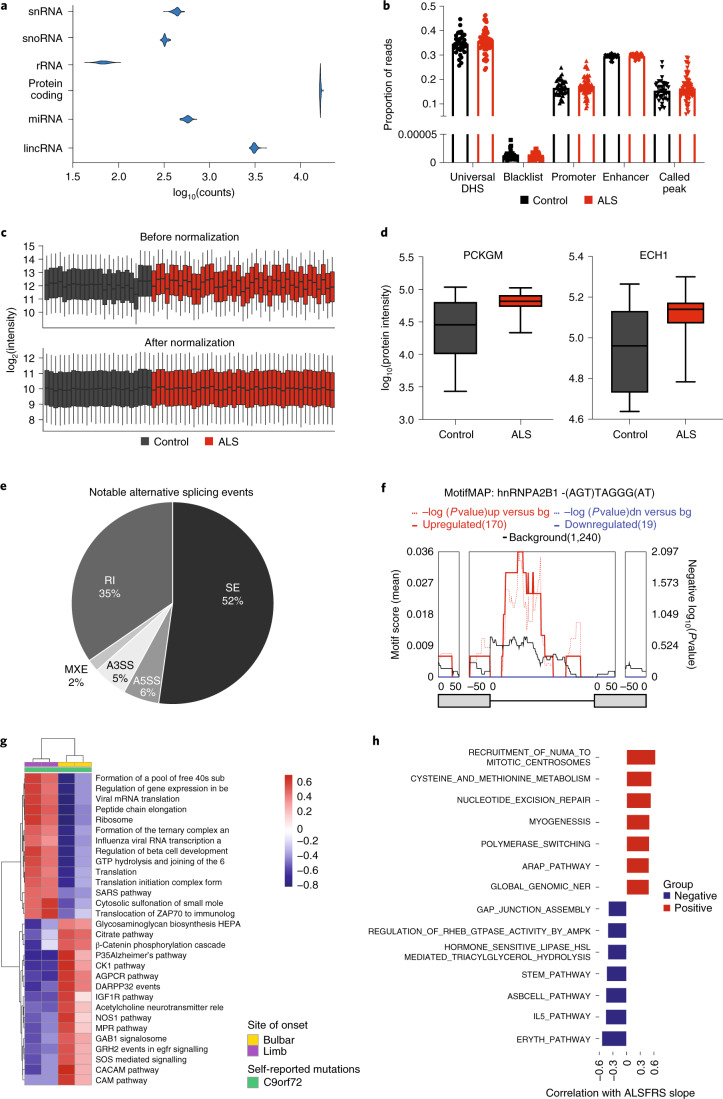
Omics exploratory analysis of results. **a**, Violin plot showing counts of RNA species identified in the current AALS samples. As expected, protein-coding and lincRNAs represent the largest proportions whereas rRNAs, which have been depleted, are the lowest. Minimal variability has been observed among samples. Types represented are: protein coding, lincRNA, miRNA, small nuclear RNA, small nucleolar RNA and rRNA in green, red, gold, purple, blue and teal, respectively (*n* = 102 biologically independent samples). **b**, Peak functional annotations. Analysis of read distribution across all ATAC-seq samples shows an enrichment in known open chromatin regions, such as DNase 1-hypersensitive sites and previously annotated enhancers and promoters (*n* = 100 biologically independent samples). **c**, The log_2_(protein intensity distribution) unnormalized (top) and normalized (bottom). **d**, The log_10_(protein intensity) comparison of selected proteins (PCKGM, ECH1) showing differential expression between ALS and controls. Box plots in **c** and **d** indicate median, quartiles and range (*n* = 66 biologically independent samples). **e**, Pie chart of proportions of rMATS analysis of differentially alternative splicing identified events comparing male *C9orf72* ALS samples versus male controls. An FDR cutoff of 0.05 was used to define statistical significance. SE has the highest number of events (*n* = 617, 52%), followed by RIs (*n* = 409, 35%). **f,** The rMAPS2-based motif enrichment analysis of alternatively RIs (409 RI events) shows that the RBP-binding motif HNRNPA2B1 is significantly enriched in the male control samples versus male *C9orf72* ALS samples near the RI sites. Wilcoxon’s rank-sum test (one sided) was used to get the *P* values for comparing up- and downregulated exons (RI) versus control/background exons. Motif scores are plotted in solid lines and *P* values are in dotted lines. Red designates control samples and blue the ALS. **g**, Heatmap of pathway activity scores defined by GSVA against MsigDB’s C2 canonical pathways from KEGG and Biocarta. The top 30 pathways are shown from comparing samples with bulbar versus limb ALS disease onset (FDR < 0.05)**. h**, The top 14 pathways that have high Pearson’s correlation between GSVA enrichment scores and ALSFRS clinical progression slope.

Annotation of transcripts detected in the samples revealed various RNA species that were captured in the deep sequencing, with protein-coding RNAs accounting for most (~82%) of all RNAs, followed by long intergenic noncoding (linc)RNA (~13%) (Fig. 5a). A low proportion of reads mapped to small RNAs and a very minimal portion to ribosomal RNAs, which were depleted during library preparation and act as a technical quality assessment. The use of total RNA-sequencing (RNA-seq) and deeper sequencing allows for differential alternative splicing analyses, as well as circular RNA and cryptic exon analyses (Fig. 5e,f). As an example of RNA-seq analyses, we assessed the ability of our cell model and RNA-seq methods to capture common, alternative splicing types and found significant enrichment in skipped exon (SE, 52%) and retention of introns (RIs, 35%) when comparing male C9 samples with male controls (Fig. 5e). RNA-binding protein (RBP) motif enrichment analysis of the significant RI events (cryptic exons) predicts that the binding of HNRNPA2B1 (Fig. 5f) is upregulated in ALS samples. These findings are consistent with previous reports in human postmortem brain tissue^16^.

To assess pathway activities, we used gene set variation analysis (GSVA) to score samples against canonical Kyoto Encyclopedia of Genes and Genomes (KEGG) and Biocarta pathways from the MsigDB database, and identified pathways that are differentially regulated between subjects with bulbar and limb onset (Fig. 5g). Using these pathway activity scores, we also identified pathways that are positively or negatively correlated with the patient ALSFRS progression slope (Fig. 5h).

These data indicate that both gene expression differences and RNA-splicing differences could be captured by our differentiated iPS cell model. Notably, these data can be explored for additional new alterations in ALS and potential associations with ALS subtype and clinical data, and with other omics data that are being captured from these samples.

### Epigenomics

**Extended Data Fig. 6:**
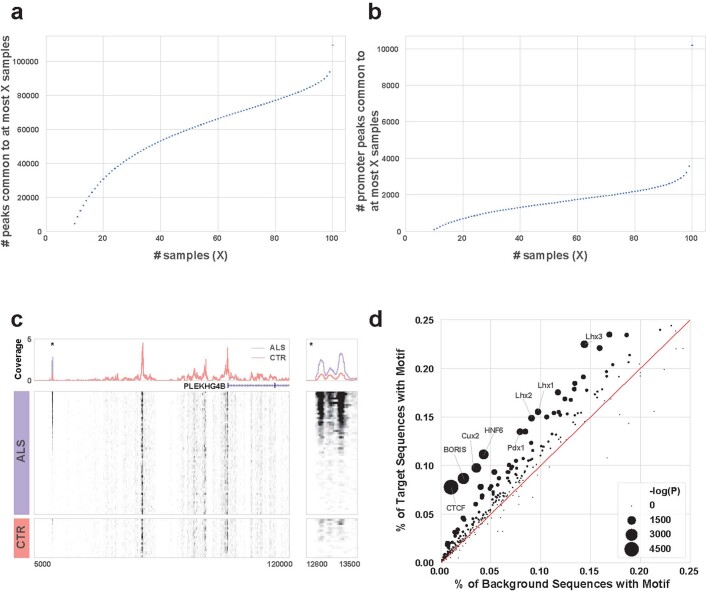
ATACSeq data. **a** and **b**. CDFs. The number of all peaks **(a)** and promoter peaks **(b)** that are common to different numbers of samples. **(c)** PLEKHG4B locus. (Left) ATAC-seq read density upstream of the PLEKHG4B gene for ALS (middle) and CTR (bottom) samples. Average coverage for each group is shown at the top. (Right) Zoomed in region around the starred peak. **d**. Motifs. The most overrepresented genomic motifs corresponding to known transcription factors as determined by the HOMER discovery algorithm for ATAC-seq. Motifs for transcription factors implicated in neuronal identity, such as Pdx1, Cux2, and the Lhx family, are significantly enriched.

Overall the quality of transposase-accessible chromatin using sequencing (ATAC-seq) data was high, with very good reproducibility of BDCs and BTCs, as assessed by the simple error rate estimate (SERE) (Fig. 5b, Extended Data Figs. 4b,e and 5b, and Supplementary Information). Hypersensitive sites were distributed across the genome in the expected regions (Extended Data Fig. 6a,b), especially in previously annotated regulatory regions, with very few reads in ENCODE blacklist regions. Although, overall, samples did not cluster by genotype or disease status, many loci did show strong differences between patients and controls (Extended Data Fig. 6c). As an example of a potential application of the epigenomic data, we identified potential transcriptional regulators through analysis of sequence motifs in the open chromatin (Extended Data Fig. 6d). Consistent with the expected cell composition, we observed an overrepresentation of transcription factors implicated in neuronal differentiation, such as Pdx1, Cux2 and the Lhx family (Extended Data Fig. 6d).

### Proteomics

In total, >25,000 peptides corresponding to >3,600 proteins per sample were quantified. As detailed in the Supplementary Information, for proteomic analytics, there was minimal drift between the batches (Fig. 5c and Extended Data Figs. 4c,f and 6c). Although patient and control iPS neuron clusters are interspersed, indicating their overall similarity, these iPS neuron models have significant individual protein-level differences and we selected representative proteins ECH1 and PCKGM (Fig. 5d) that show significant (*P* ≤ 0.05) differences, based on what is seen in the differential analysis-based evidence (Fig. 5d).

### Longitudinal single-cell imaging and analysis

**Fig. 6:**
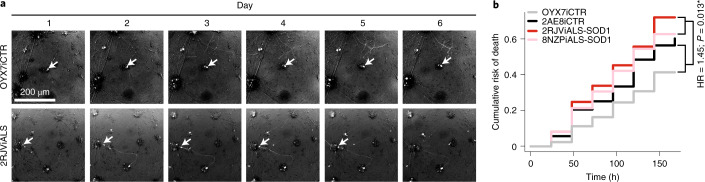
Progressive degeneration of spinal neurons derived from patients with mutant *SOD1* (diMNs) was detected by longitudinal robotic microscopy. **a**, Example images of iPS diMNs over time. Control (top OYX7iCTR) and SOD1-ALS (2RJViALS) lines were transduced with the fluorescent reporter Synapsin::EGFP^33^ and differentiated for ~24 d. Cells were imaged every 24 h starting at day 24 (day 1) using robotic microscopy. Although some of the diMNs are clumped in cell clusters, sparse transfection and robotic microscopy enable them to be tracked over time (soma indicated by white arrowheads). Control neurons survive the duration of the experiment; SOD1-ALS neurons degenerate at the last time point. **b**, Longitudinal robotic imaging of mutant *SOD1* iPS spinal neurons (2RJViALS-SOD1; 8NZiALS-SOD1) compared with two control iPS spinal neuron lines (OXY7iCTR; 2AE8iCTR) revealing time-dependent in vitro neurodegeneration. The diMNs were subjected to robotic microscopy for 7 d starting on differentiation day ~20. The rate of cell death was tracked over time and compared across lines using Cox’s proportional hazards. The diMNs from patients with ALS that harbor *SOD1* mutations (2RJViALS: *n* = 3 and 391 neurons; 8NZPiALS: *n* = 2 and 221 neurons) die faster than controls (2AE8iCTR: *n* = 2 and 192 neurons; OYX7iCTR: *n* = 3 and 291 neurons). HR (hazard ratio) = 1.45; *P* = 0.013. Future studies of sporadic lines will be incorporated into the AALS data portal.

Validation of the identification of pathological phenotypes was achieved with longitudinal single-cell robotic imaging of mutant *SOD1* patient-derived iPS spinal neurons as described previously (Fig. 6a)^17^. As shown in Fig. 6b, mutant *SOD1* neurons exhibited an enhanced cell death profile, similar to that reported previously with spinal motor neurons^18^. Future data will be available on similar analytics of cohorts of the sporadic iPS cell-derived neurons from the AALS dataset.

### Data dissemination: data portal

The AALS data portal (http://data.answerals.org; Supplementary Table 3) was designed to provide information about the various types of biological and clinical data generated by the AALS partners and to allow easy visualization/access to the metadata and data, along with links to obtain biofluids and iPS cell lines. Additional details regarding the portal can be found in Extended methods. In the future, the portal will also host online data analytics and visualization tools.

## Discussion

The pathogenesis of sALS remains a mystery and few comprehensive data collections, on a population scale, exist to truly inform researchers about the biological underpinnings of the disease or the possibility of disparate biological subgroups. To date, clinical studies alone have not yielded reliable data to suggest a common pathway or, more importantly, a means to target relevant biological subgroups. The identification of biological subgroups has been impactful in various cancers, where the ability to actually sample disease tissues from skin, liver, prostate or pancreas biopsies, coupled with clinical characteristics of tumor type, has led to marked improvements in therapeutic approaches, drug treatments and decisions about disease management^19,20^.

The core goal of AALS is to provide a comprehensive set of tools including deeply phenotyped longitudinal clinical data and biological tools such as iPS cell lines, and a multi-omics platform consisting of whole-genome, iPS-derived, spinal neuron-enriched proteomes, transcriptomes and epigenomes, to uncover underlying biological subgroups. Previous studies have demonstrated the ability to generate small populations of fALS or sALS iPS cell-derived motor neurons and glia, as well as relatively limited multi-omics data. However, none approximates true population-based tools, with reproducible quality assurance protocols, necessary to accurately assess disease pathways or identify population subgroups combining longitudinal clinical, genomic and living multi-omics data^4,21,22^.

The AALS reagent collection includes individual iPS cell lines from approximately 850 sALS and control participants (soon to reach >1,200), the iPS cell-derived spinal neurons from each participant, their longitudinal clinical data (collected over 1 year), sequentially amassed fluid biospecimens (blood and cerebrospinal fluid (CSF)) and the early multi-omics data generated from each participant’s blood (whole genome) as well as from their ‘spinal cord biopsy’-equivalent, iPS-derived neuronal cell lines. The collection also includes autopsy samples and pathology data from a subset of participants. The autopsy pathology data and CNS specimens will eventually be available through the AALS web portal and coupled with the iPS cell lines from these participants.

A reasonable question is the utility of patient-derived iPS cells to predict the disease-causing pathways in an adult-onset disease. Can reprogrammed human spinal neurons reflect adult-onset disease pathogenic cascades? Already multiple studies have documented that human iPS cell lines, in either two-dimensional cultures or three-dimensional organoids, can reproduce the pathology seen in human brain^23–25^. One advantage of the iPS platform is the ability to dynamically detect early pathogenic events and even serially occurring events. In fact, early use of the AALS iPS cell lines has already provided evidence that the iPS collection can provide insights into new pathways (nuclear pore complex and nuclear transport defects) in ALS pathophysiology, generate new therapies and validate gene therapy based on the approaches^4,25–27^.

This population and its dataset were never envisioned to enable the identification of new ALS genes. A cohort of ~1,000 ALS participants does not amount to a large enough database for new gene identifications. However, sharing the whole-genome sequences from this dataset has aided in the identification of a new ALS gene, *Kif5A*^28^. In fact, the estimated 6+ billion data points generated from each participant, combining the longitudinal clinical demographic and observational data, the longitudinal smartphone app data (motor activity, speech, breathing, cognition) and the aggregate multi-omics data (whole genome, epigenome, proteome, transcriptome) represent an exceptionally large set of data per participant. Furthermore, the core multi-omics dataset reflects the human cells affected in individual ALS participants and spinal neurons, and acts as an organ- or tissue-specific biopsy. When these combined longitudinal and multidimensional clinical and biological data are analyzed by integrative methods, such as artificial intelligence, clinical and biological subgroups might emerge, potentially assigning a unique risk or modifier gene or a unique molecular pathway to a specific patient subgroup, which could one day enable patient-specific interventions, or serve as drug target engagement marker or subgroup biomarker.

How many individual sporadic patient lines would be required to detect one of more pathophysiologically relevant subgroups is simply not known. Prior work in fALS suggests that at least 10–15 C9orf72 iPS cell lines is sufficient to robustly detect defects in nuclear pore biology. However, sALS may have multiple risk pathways associated with gene variants (for example, ataxin 2 expansion, TMEM 106b)^29,30^ or environmental stressors and, as such, may require more patient cell lines and multi-omics data to allow detection of robust pathway readouts. A recent study, targeting imaging-based strategies to detect and evaluate an ESCRT-III-based pathway and therapy in >40 different sporadic and *C9orf72* ALS and control iPS cell lines, approached the size of a small clinical trial^25^. However, it remains unclear how many iPS cell lines are needed to robustly and reproducibly detect pathophysiological alterations from human omics analyses.

The other research advantage to such a dataset and living tools is the immediate ability to test for potentially ALS-relevant pathogenic pathways using the participant’s own iPS cells/iPS cell-derived spinal neurons to test drugs for candidate pathogenic pathways and, importantly, to develop CNS biomarkers from the iPS cells and validate drug target engagement. Libraries of iPS cell lines derived from participants with neurological diseases, including Alzheimer’s disease and FTD, have been growing over the last several years and represent a valuable tool to truly examine specific disease pathways^31,32^. Most of these iPS cell libraries are relatively small, including our original library of 22 fALS iPS cell lines^21^, with a few selected lines for each disease mutation and, when appropriate, isogenic controls. None represents the far more common sporadic forms of the disease. Furthermore, none provides deep longitudinal clinical and extensive multi-omics data.

Aside from the biological data generated from the program, the results from the AALS smartphone app demonstrate that the modules implemented to assess limb function, speech and cognition may be useful to identify early bulbar and cognitive symptoms in ALS and track disease progression over time. Specifically, limb-function tests reveal that it can be useful to infer ALSFRS-R scores. Importantly, we observed that, by combining the features from multiple domains, motor tests and all the voice tests highly correlated with the ALSFRS, now commonly used as a primary or secondary outcome measure in ALS clinical trials, thereby providing a reliable tool for at-home longitudinal monitoring of patient progression. Furthermore, the single-breath testing also correlated well with in-clinic forced vital capacity (FVC), often a prominent secondary outcome measure in clinical trials. This test typically requires in-clinic testing, which limits enrollment or follow-up data collection in clinical trials. The application of this app test alone could greatly enhance patient participation in nationwide clinical trials—especially in those areas where travel to a testing center is challenging. Overall, we observe that quantitative motor speech analysis holds tremendous promise in both identifying changes limited not only to ALS rating scales but also to others such as cognitive assessment. The potential to record voice, and store it encrypted in the cloud, could provide a powerful clinical tool to assess change over time for use clinically and in ALS trials. Overall, the app data, coupled with in-clinic data, provide deep and longitudinal clinical datasets available for multi-domain biological and clinical correlations for future users.

The overall clinical demographics and population genomics in the AALS program accurately reflect the ALS subject population described in previous studies. This observation validates the AALS iPS cell lines and multi-omics platform as a database that others can employ to generate and test biological hypotheses.

Importantly, all the clinical data, multi-omic data and iPS cell lines were generated to be freely accessible to all researchers, academic and commercial, free of restrictions other than standard Health Insurance Portability and Accountability Act (HIPAA) compliance rules. A web portal for downloading filtered datasets, for example, proteome, whole genome, etc., has been set up with minimal but appropriate requirements for data access (Supplementary Table 3). The ALS and control iPS cell lines, matched to datasets, are also fully available for research studies, for a minimal fee (to cover the replacement of the depleted stock of cells). Biospecimens (for example, CSF and plasma) longitudinally collected from patients are also available (Supplementary Table 3). Future web-based links will include access to autopsied CNS tissues from patients matched to the iPS cell lines and iPS cell-based multi-omics.

## Methods

### Program process

#### Overall design (Extended Data Fig. 1)

The overall AALS program, from clinical enrollment to smartphone app data collection, iPS cell-line generation, biological data generation and data storage is outlined in Extended Data Fig. 1 (ClinicalTrials.gov: NCT02574390). Methods for each element of the program are provided below and in Supplementary Methods.

### Enrollment, clinical characterization and sample collection

The clinical portions of AALS were coordinated through Johns Hopkins University and Massachusetts General Hospital. The eight enrolling neuromuscular clinics were distributed across the USA and included Johns Hopkins University, Massachusetts General Hospital, Ohio State, Emory University, Washington University, Northwestern University, Cedars-Sinai and Texas Neurology (Supplementary Table 1 and Extended Data Fig. 1). The study was approved by local institutional review boards, and all participants provided written informed consent. Consent was uniform across all sites and included agreement to share data broadly for medical research (also see Data access in Supplementary Information). Subjects with sALS, fALS and related MNDs (referred to as non-ALS MNDs), including those with primary lateral sclerosis, progressive bulbar palsy and progressive muscular atrophy, along with asymptomatic ALS gene mutation carriers, were enrolled in AALS. Age-matched control participants without ALS or a family history of ALS were also enrolled. Additional enrollment details are provided in Supplementary Information.

Participants were monitored every 3 months for a year and, when possible, the ALSFRS-R was conducted by telephone every 3 months for another year thereafter. Baseline descriptors included the following: demographics and vital signs, genetic and family history of MND, general medical history, CNS lability and a brief focused history of environmental exposures. Concomitant medications and past medical history were collected at enrollment and updated throughout study participation. Measures of ALS progression included: deep tendon reflexes, Ashworth Spasticity Scale, Hand Held Dynamometry, ALSFRS-R and pulmonary slow vital capacity (Supplementary Tables 2 and 3 and Supplementary Information). To enhance depth of longitudinal clinical data collection, a secure and HIPAA-compliant smartphone app, with a specific focus on motor activity, voice and cognition, was created for home data collection (Fig. 2 and Extended Data Fig. 2). At each in-clinic visit, blood was collected and processed according to the methods outlined in Supplementary Information. At the first visit, whole blood was collected for generation of primary peripheral blood mononuclear cell (PBMC)-derived iPS cell lines.

### Biofluid collection and processing

At each in-clinic visit along with follow-up visits, approximately 50–100 ml of blood was collected from each participant. Plasma and serum were processed for storage and PBMC isolation. Whole blood was sent to the NYGC for DNA extraction and WGS. CSF was optionally collected and flash frozen at −80 °C. Serum, plasma and CSF samples were shipped on dry ice to a centralized biofluid repository to be stored at −80 °C (Supplementary Table 3). Additional details are provided in Supplementary Methods

### Return of AALS results

To provide medical and ethically appropriate feedback, study participants with ALS were offered the opportunity to receive the results of their WGS for 5 ALS genes (*C9orf72*, *SOD1*, *FUS*, *TARDBP* and *TBK1*), as well as 59 genes designated as medically actionable by the ACMG^15^, as part of a substudy, Return of Answer ALS Results (ROAR). ROAR participants completed a separate online consent after enrollment in the parent study. Additional details are provided in Supplementary Information.

### AALS smartphone app

The app has seven modules designed to gather information about upper limb motor function, respiration, bulbar function and cognition. Six modules measured arm function: finger tapping, finger tracing and phone tilt tracing; each was performed using the right and left hand separately (Fig. 2a). The speech module (Fig. 2c), consisted of three tasks, rotated weekly to reduce learning effect: (1) single-breath count, in which participants were instructed to draw in a deep breath and count at a measured pace (a surrogate for FVC)^34^; (2) read-aloud passage, in which participants read aloud one of four standardized passages from their screen; and (3) picture description, in which participants described one of three line-art illustrations over 30–120 s. Details regarding this digital clinical module are included in Supplementary Information.

### The iPS cell-line methods

#### PBMC processing

Fresh blood was collected, and samples were centrifuged at 18–25 °C in a horizontal rotor centrifuge for 20 min at 1,800 r.c.f. within 2 h of collection. The plasma/buffy coat mixture was collected and centrifuged for 15 min at 300 r.c.f. Isolated PBMCs were counted and cryopreserved. The average cell count was ~25 million PBMCs per sample with an average cell viability of 91%. Additional details are provided in Supplementary Methods

#### Generation, reprogramming and QC of iPS cells

The iPS cells were generated by reprogramming the cryopreserved and nonexpanded PBMCs, using a method based on a nonintegrating episome. Clones were isolated, expanded and maintained according to standard feeder-free protocols and characterized extensively as described in Supplementary Table 6. The iPS cell lines were generated from ~25 patients per month and stored frozen until they were differentiated (Extended Data Fig. 3a). Each cell line was thawed and cultured for 2–3 weeks before passaging for differentiation. Cell lines were differentiated in batches of up to 11 lines. PBMCs were used instead of fibroblasts to limit the potential for genetic defects and facilitate sampling from the large number of patients enrolled in our study. Overall, blood draws are less invasive and carry lower risk for patients than skin biopsies, which improved the overall risk–benefit ratio for the study. Rigorous quality control (QC) (Supplementary Table 6) was performed on each AALS iPS cell line, similar to previously publications^35^. G-band karyotype was performed at multiple passages for each AALS iPS cell line, which provides confidence about the genetic integrity of the AALS iPS cell repository, given that each iPS cell line is karyotyped at multiple passages. Cell-line authentication is repeated at multiple stages. The cell line authentication (STR) is performed on the original donor blood/PBMC sample, then performed on the reprogrammed iPS cell line and the differentiated neurons (Supplementary Tables 6 and 19). Additional details are provided in Supplementary Information.

#### Generation of iPS cell spinal neurons

The iPS cells were differentiated into motor neurons according to the direct iPS cell diMN protocol, which comprises three main stages (Extended Data Fig. 3 and Supplementary Table 6), as described previously^25^. Additional details are provided in Supplementary Information. On day 32 of differentiation, cell lines were collected and pelleted as illustrated in Fig. 4. Thus far, ~800 iPS cell lines have been successfully reprogrammed and one clone line banked and characterized per donor. Out of the ~800 unique samples, only 18 lines (~3%) failed reprogramming. Additional details are provided in Supplementary Information.

#### QC of diMNs

As referenced in Extended Data Fig. 3, on day 32 one 6-well plate from each cell line for immunostaining was reserved for QCs, which included the following markers of neuronal differentiation: SMI32 (NF-H), TUBB3 (TUJ), ISL1, NKX6.1, S100β and Nestin. This protocol generates a mixed population of neurons consisting of ~75% (±8%) β_III_-tubulin- (TuJ1-) and ~70% (±10%) NF-H-positive cells, ~19% (±6%) Islet-1- and ~34% (±9%) Nkx6.1-positive spinal motor neuron, and ~18% (+/13%) S100B-positive progenitors 32 d after the onset of differentiation (Fig. 3). Additional details are provided in Supplementary Information.

### Multi-omics data generation for each iPS cell-derived motor neuron line

At the end of the 32-d differentiation protocol, the spinal neurons were harvested for RNA-seq, proteomics or epigenome profiling as detailed in Supplementary Methods. WGS was performed on PBMCs. Day 32, chosen for independent experiments with selected *C9orf72* ALS/FTD iPS cell-derived spinal neurons, demonstrated phenotypic and molecular changes in nuclear pore complex and biology, matching that seen in patient autopsies, by this time point^26^.

#### Program QCs: cell generation batch controls

To detect and compensate for cell culture-associated confounders, all differentiations were conducted in a single facility and included two key control groups of biological samples: BDCs were differentiated with each batch from the same original line to assess interbatch variability of iPS cell differentiation to diMNs and BTCs, consisting of a single differentiation of the same line were frozen, aliquoted and distributed with each batch to assess technical variability of the omics assay batch runs, were performed as detailed in Supplementary Information. Complete details for the design and implementation of these critical operational controls (Extended Data Figs. 4 and 5) can be found in Supplementary Information.

### Data quality and batch effect assessments

#### RNA-seq

For the RNA-seq data samples were processed and passed all QC metrics including RNA integrity (Extended Data Fig. 4a), library and sequencing QC metrics. To assess data quality and technical batch effects, sample-to-sample SERE scores (0 = identical samples) were generated using gene expression for three groups: the BDCs, BTCs and all other samples (Extended Data Figs. 4 and 5).

A heatmap of SERE scores between all samples with hierarchical clustering (Extended Data Fig. 5) shows that, although BTCs form their own cluster, the rest of the samples fall into multiple small clusters with no clear relationship to their disease status.

#### Proteomics

Each block of samples comprised case, control, BDC samples and HEK293 cell control samples. The numbers of proteins and peptides quantified for all 66 samples were very consistent (Extended Data Fig. 4c). The percentage coefficient of variation for the proteins quantified were calculated for the BTC and BDC samples (Extended Data Fig. 4f). Individual samples are normalized to the total MS2 spectra intensity across the chromatographic profile of eluting peptides to smooth any inconsistencies in sample loading on to the mass spectrometry (MS) instrument, thereby eliminating systemic variation in signal intensities (Extended Data Fig. 4c). We found that BTCs and BDCs (both originating from the 2AE8 CTR cell line) cluster tightly (Extended Data Fig. 6c), indicating minimal drift between the MS batches.

#### Epigenetics

ATAC-seq data quality was determined according to ENCODE^36^. The distribution of fragment sizes across all samples revealed a clear nucleosome-free region and regular peaks corresponding to nucleosomal fractions (Extended Data Fig. 6). As expected, replicates from our batch control line were highly correlated with each other, with BTCs having an even smaller variation in correlation values compared with BDCs (Extended Data Fig. 4e). We also generated a consensus set of peaks present in >10% of samples using DiffBind (Extended Data Fig. 6) and characterized transcription factor motif enrichment within these peaks using HOMER^37^. There was an overrepresentation of transcription factors implicated in neuronal differentiation, such as Pdx1, Cux2 and the Lhx family (Extended Data Fig. 6d). We then obtained a counts matrix of reads mapped to each peak in the consensus peakset across all samples and performed hierarchical clustering using the same approach as the RNA-seq data (Extended Data Figs. 4, 5 and 6). Subjects did not cluster by disease status, presence of C9 mutation, sex or processing batch. Additional data on quality control can be found in Supplementary Methods.

### Whole-genome methods: WGS and analysis

PBMCs were sent by each clinic to the NYGC (https://www.nygenome.org) for DNA extraction and sample QC and WGS libraries. We evaluated pathogenic or probable pathogenic variants reported in ClinVar (C-PLP) for all genes. We also examined pathogenic variants called by Intervar Li^38^ (I-PLP) and predicted damaging variants as called by in silico prediction tools (IS-D), which are reported in Table 2 and Supplementary Table 8. The variant calls from NYGC were assessed by examining the actual reads for alignment issues and spot checking the BAM files for specific variants in Integrative Genomic Viewer determined to be of good quality. The variant call formats (VCFs) were converted into genomic VCFs (GVCFs), and joint genotyping calling was run using Sentieon v.201911 (https://www.sentieon.com); applied variant quality score recalibration (VQSR) was done using GATK v.3.8 (truth sensitivity level = 99.0), and the files were annotated using Annovar v.2018Apr16 (ref. ^39^). For each variant, we also incorporated functional in silico predictions from nine programs, including databases such as SIFT^40^, PolyPhen2 (ref. ^41^) and Mutation Taster^42^, and those described in Li et al.^43^. Additional databases were included that assess the variant tolerance of each gene using the Residual Variation Intolerance Score (RVIS)^44^ and the gene damage index (GDI)^45^ and LoFTool^46^. For variants in genes that are highly expressed in the brain, we incorporated data from the Human Protein Atlas^47^ (http://www.proteinatlas.org) and expression data from GTEx portal^48,49^ (https://gtexportal.org/home) for the cortex and spinal cord. Frequency information from three databases on all known variants was obtained from ExAC^50^, the National Heart, Lung, and Blood Institute (NHLBI) Exome Sequencing Project (ESP)^51^ and the 1000 Genomes Project^10^.

PCA was carried out (Fig. 4d) to reveal how the AALS samples cluster among various ancestry groups of the 1000 Genomes Project dataset. PCA was used^12,13^ to visualize the ancestry background of the AALS cohort and a set of 2,504 samples from the 1000 Genomes Project with well-defined ancestry. We used a set of 10,000 randomly chosen autosomal SNPs (singletons and multiallelic SNPs were removed) that were present in both datasets and removed correlated SNPs by linkage disequilibrium pruning. We implemented randomized PCA^52^ using the Python library scikit-allel package^53^.

The annotation pipeline incorporated elements from ANNOVAR^39^ and generated reports, including genotypes for all samples. These reports are available on request. The following annotation was used: for genes and exonic variants that have clinical significance, the Clinical Genomic Database^54^, the Online Mendelian Inheritance in Man^55^ and ClinVar^56^, and genes listed in the ACMG^57^ database were incorporated. We also incorporated Intervar, which is based on the ACMG and AMP standards and guidelines for interpretation of variants^58–61^. This tool uses 18 criteria to prescribe the clinical significance and classifies based on a 5-tiered system^62^. To flag ALS genes, ALS gene lists and variants were incorporated from ALSoD^63^ (http://alsod.iop.kcl.ac.uk), a list provided by M. Harms, a gene list from J. Landers and associations from DisGeNet^64^. Functional predictions were based on in silico prediction from nine databases: SIFT^40^, PolyPhen2 (refs. ^65–67^) (HDIV and HVAR), LRT_Prediction^67^, Mutation Taster^42^, Mutation assessor^68^, FATHMM prediction^69–71^ and dbNSFP (RadialSVM_pred and LR_pred)^72–74^. Databases that assess the variant tolerance of each gene using the RVIS^44^ and the GDI^45^ were also included, and LoFTool^46^ will be incorporated. To identify variants in genes that are highly expressed in the brain, data from the Human Protein Atlas^47^ (http://www.proteinatlas.org) and the GTEx portal^75,76^ (https://gtexportal.org/home) for the cortex and spinal cord were used. Frequency information was derived from ExAC^50^, the NHLBI ESP^51^ and the 1000 Genomes Project^11^.

A separate annotation pipeline was developed for variants in intergenic and regulatory regions. Variants are reported relative to the closest gene, whether intronic, upstream and downstream (up to 4 kb from the start and stop of a gene) or in 5′- and 3′-UTRs. The annotation was based on RegulomeDB, which annotates variants with known or predicted regulatory elements such as transcription factor-binding sites, expression quantitative trait loci, validated functional SNPs and DNase sensitivity^77^, with source data from ENCODE^78,79^ and the Gene Expression Omnibus^80^. Additional regulatory databases such as Target Scan, an algorithm that uses 14 features to predict and identify microRNA (miRNA) target sites within messenger RNAs^81^ and miRBase^82–84^, were also used. Extensive details on the methods for whole-genome analytics can be found in Supplementary Methods.

### RNA methods

Total RNA was isolated from each sample using the QIAGEN RNeasy mini-kit. RNA QC was conducted using an Agilent Bioanalyzer and Nanodrop. Our primary QC metric for RNA quality is based on RNA integrity number (RIN) values ranging from 0 to 10, 10 being the highest quality RNA. In addition, we collected QC data on total RNA concentration and 260:280 and 260:230 ratios to evaluate any potential contamination. Only samples with RIN > 8 were used for library prep and sequencing. The rRNAs were removed and libraries generated using TruSeq Stranded Total RNA library prep kit with Ribo-Zero (QIAGEN). RNA-seq libraries were titrated by quantitative (q)PCR (Kapa), normalized according to size (Agilent Bioanalyzer 2100 High Sensitivity chip). Each complementary DNA library was then subjected to 100 Illumina (Novaseq 6000) PE sequencing cycles to obtain over 50 million PE reads per sample. After sequencing, raw reads were subject to QC measures and reads with quality scores >20 collected and analyzed. Reads were mapped to the GRCh38 reference genome using Hisat2, QCed and gene expression quantified with featureCounts^85^, and differential expression was quantified using DESeq2 (ref. ^86^). Normalized and transformed count data were also used for exploratory analysis and differentially expressed genes (false discovery rate (FDR) < 0.1) were analyzed with commercial and open-source pathway and network analysis tools, including Ingenuity Pathway Analysis, gene set enrichment analysis (GSEA), GOrilla, Cytoscape and other tools to identify transcriptional regulators, predict epigenomic changes and determine potential effects on downstream pathways and cellular functions.

### ATAC-seq methods

We used the assay for ATAC-seq to assess chromatin accessibility and identify functional regulatory sites involved in driving transcriptional changes associated with ALS. ATAC-seq sample prep, sequencing and peak generation were carried out by Diagenode Inc. as further described^87^. Briefly, cells were lysed in ATAC-seq resuspension buffer (RSB; 10 mM Tris-HCl, pH 7.4, 10 mM NaCl, 3 mM MgCl_2_ and protease inhibitors) with a mixture of detergents (0.1% Tween-20, 0.1% NP-40 and 0.01% digitonin) on ice for 5 min. The lysis reaction was washed out with additional ATAC–RSB containing 0.1% Tween-20 and inverted to mix. Then 50,000 nuclei were collected and centrifuged at 450 r.c.f. for 5 min at 4 °C. The pellet was resuspended in 50 µl of transposition mixture (25 µl of 2× Illumina Tagment DNA buffer, 2.5 µl of Illumina Tagment DNA enzyme, 16.5 µl of phosphate-buffered saline, 0.5 µl of 1% digitonin, 0.5 µl of 10% Tween-20 and 5 µl of water). The transposition reaction was incubated at 37 °C for 30 min followed by DNA purification. An initial PCR amplification was performed on the tagmented DNA using Nextera indexing primers (Illumina). Real-time (RT)-qPCR was run with a fraction of the tagmented DNA to determine the number of additional PCR cycles needed, and a final PCR amplification was performed. Size selection was done using AMPure XP beads (Beckman Coulter) to remove small, unwanted fragments (<100 bp). The final libraries were sequenced using the Illumina NextSeq platform (PE, 75-nt kit). All samples passed QC checks that included morphological evaluation of nuclei, fluorescence-based electrophoresis of libraries to assess size distribution and RT-qPCR to assess the enrichment of open chromatin sites. The quality of the sequencing was assessed using FastQC and the reads were aligned to GRCh38 genome build using Bowtie2. We identified open chromatin regions separately for each sample using the peak-calling software MACS2 (ref. ^88^) and determined differentially open sites using DESeq2 (FDR < 0.1). Peaks were assigned to unique genes using the default HOMER^37^ parameters, and gene ontology analysis was performed using GOrilla^89^.

### Proteome methods

Whole-proteome extracts from frozen diMNs were digested with trypsin and LysC and subjected to acquisition on the SCIEX 6600 as detailed below. Snap-frozen cell pellets were stored at −80 °C and transferred to the Cedars-Science Medical Center proteomics lab on dry ice, where it was stored at −80 °C until use. Samples were lyophilized and aliquoted into 600-µl polystyrene microcentrifuge tubes containing lysis buffer (6 M urea and 1 mM dithiothreitol in 1.5 M NH_4_HCO_3_). The sample was sonicated (QSonica Q800R1) by alternating 10 s on and 10 s off at 70% amplitude while rotating in a 4 °C water bath until the solution was homogenized (~20 min). Samples were centrifuged and the protein concentration determined on the supernatant according to manufacturer’s instructions (Pierce BCA Protein Assay Kit). Then 200 µg of each sample was transferred to a 96-well plate in aliquots and processed on the Biomek i7 Automated workstation (Beckman Coulter) as outlined previously. Briefly, samples underwent the following: reduction of disulfide bonds in 3 mM tris(2-carboxyethyl)phosphine hydrochloride solution, alkylated in 5 mM iodo-3-acetic acid. Addition of β-galactosidase at 2 µg and protein digestion in solution using equimolar trypsin and LysC enzyme mixture (Promega, catalog no. V5111) followed at 1:40 enzyme:protein ratio under optimized digestion conditions (4 h at 37 °C). Digested proteins were desalted on a 5-mg Oasis HLB 96-well plate (Waters, catalog no. 186000309) and eluted in 50% acetonitrile. Samples were dried to completion using a speed-vac system and stored at −80 °C until MS analysis. For MS analysis, digested peptides were resuspended in 0.1% formic acid (FA) and analyzed on a 6600 Triple TOF (Sciex) in data-independent acquisition (DIA) mode and on the 6600 Triple TOF (Sciex) for data-dependent acquisition (DDA) mode. Specifically, samples were acquired in DDA mode for ion library generation and in DIA mode over 100 variable windows, similar to previously described acquisition protocols^90,91^.

DDA data were used for the generation of a sample-specific peptide ion library. DDA files were run through a *trans*-proteome pipeline using a human canonical FASTA file (Uniprot). A consensus peptide library with decoys was generated and used to quantify ions identified in DIA data files. Previously described DDA library build principles^92^ were utilized to generate a cell-specific library, which allowed for greater accuracy in matching DIA data to the DDA library during OpenSWATH, as indicated by higher *d* scores in PyProphet. The differential protein expression between ALS and control samples analyzed was calculated using mapDIA^93^.

DIA data files were analyzed using OpenSWATH pipeline against the sample-specific peptide ion library generated. Protein-level quantification is calculated by summing transition level intensities for all the proteotypic peptides identified. Differential protein expression between ALS and control samples analyzed was calculated using mapDIA.

### Imaging methods

#### Longitudinal single-cell imaging and analysis

Differentiated iMNs from a subset of the AALS iPS cell lines were plated on 96-well plates for longitudinal single-cell imaging using robotic microscopy as previously described^94–103^. At day 25, cells were transduced with expression marker plasmids such as synapsin::EGFP^33^ to visualize cell morphology and viability. After transduction cells were imaged in an automated fashion with robotic microscopy once per day for 10–14 d. Some image analysis was performed in a computational pipeline constructed within the open-source program Galaxy, to identify and track individual cells and perform survival analysis and other morphological measurements. Additional method details can be found in Supplementary Methods.

### Statistics

Statistical methods for the various programs are detailed in the Supplementary Information for the various programs.

### Data portal

#### Data storage and data integration/analytics

AALS was designed to be an ‘open source’ program. All of the clinical datasets, the various omics results, including whole-genome, proteome, transcriptome and epigenome, along with the data integration have been posted to a portal for data sharing and crowd sourcing (https://data.answerals.org; Supplementary Table 3). Data are available for download to all academic and commercial researchers.

##### Web-based analytics

We have included online analytics for the many ALS researchers who will neither need nor want to download the full dataset. The current set of tools available at http://data.answerals.org/analyze allows users to select genes/pathways of interest and visualize them using braid maps, heatmaps, volcano plots, bar charts or networks (Fig. 4).

The data portal provides users with information about the AALS program, the data, relevant terminology and data release notes. Users can download a metadata package associated with each versioned release. This versioned package contains comprehensive clinical, iPS cell and inventory metadata. In addition, processes for enrolling patients, producing iPS cell lines and performing WGS are explained with links provided to the relevant facilities/institutions. Explanations for sample collection and analysis of epigenomic, proteomic and transcriptomic data are available. Finally, precise definitions are provided for our data levels, which are ways to stratify all the various omics data coming from our analyses (Supplementary Table 20).

### Data dissemination

The AALS data portal (http://data.answerals.org; Supplementary Table 3) provides all raw and processed data including longitudinal clinical data and biological data generated by the AALS program, along with visualization/access to the metadata, data and biosamples released. The portal provides an overview of the data release notes, assays, data-level descriptions and links to sites for viewing cell lines/biosamples associated with the program. The website allows browsing of all available metadata (using filter and text search functions), the option to download all data and metadata or a filtered subset and links to obtain individual iPS cell lines from the Cedars-Sinai Biomanufacturing Center. Users interested in downloading datasets are required to submit an online form, acknowledge data use parameters and return a signed Data Use Agreement in compliance with the HIPAA.

### Data organization and naming

Data products were organized and named in a unified and systematic manner to allow a smooth end-user experience. Data levels (Supplementary Table 20) were employed as a categorization schema to group similar types of omics data products together. Supplementary Table 21 describes examples of these data levels in action with each experimental assay that our program collects. All data products were prefixed in a systematic manner. The prefix consists of the following components: whether the sample is from a diseased patient or healthy control patient, the de-identified patient GUID, the sample vial ID and the assay type abbreviation. An example of this is the raw transcriptomics FASTQ file CASE-NEUAA599TMX-5310-T_P10_1.fastq.gz. The first underscore separates the prefix from any supplementary file information, allowing for easy tokenization. This nomenclature is applied consistently to all metadata and data files, making it easy to establish relationships with a single study participant.

### Reporting Summary

Further information on research design is available in the Nature Research Reporting Summary linked to this article.

## Online content

Any methods, additional references, Nature Research reporting summaries, extended data, supplementary information, acknowledgements, peer review information; details of author contributions and competing interests; and statements of data and code availability are available at 10.1038/s41593-021-01006-0.

## Supplementary information

Supplementary Information (A) Expanded methods and (B) Data collection forms.

Supplementary Tables Supplementary Table 1 Clinic locations for AALS. Supplementary Table 2 AALS clinical events. Supplementary Table 3 Data and biospecimen sources. The authors declare that all data supporting the findings of the present study are available within the paper and its supplementary information files and web portals listed in this table. Supplementary Table 4 Overall clinical demographics. Supplementary Table 5 Fast versus slow progression demographics. Supplementary Table 6 Characterization and validation of IPS cell lines. SupplementaryTable 19 Cell line authentication. Supplementary Table 20 Data-level definitions. Supplementary Table 21 Examples of data levels for each assay. Supplementary Table 22 Stage 1 cell culture media. Supplementary Table 23 Stage 2 platedown media. Supplementary Table 24 Stage 2 media. Supplementary Table 25 Stage 3 media. Supplementary Table 26 Antibody reagents for iPS evaluation.

Reporting Summary

Supplementary Tables 7–18 Large Excel file-containing Tables 7–18: Supp Table 7_33-ALS-Summary; Supp Table 8_33-ALS-C-PLP Supp Table 9-33-ALS-I-PLP; Supp Table 10-33-ALS-H-PLP; Supp Table 11-33-ALS-ISD; Supp Table 12_C-PLP_All; Supp Table 13_I-PLP_All; Supp Table 14_IS-D_All; Supp Table 15-C9orf72_EH; Supp Table 16-ATXN2_EH; Supp Table 17_ACMG_Gene_ClinVar; Supp Table 18_ACMG_Gene_Intervr.

## Data Availability

All data supporting the findings of the present study are available within the paper, its Supplementary information files and the AALS web portals listed in Supplementary Table 3 (or via data.answerals.org).

## Extended data

are available for this paper at 10.1038/s41593-021-01006-0.
