# Supplementary material for: Answer ALS, a large-scale resource for sporadic and familial ALS combining clinical and multi-omics data from induced pluripotent cell lines
Source: Nat Neurosci. 2022 Feb 3;25(2):226–37. doi: 10.1038/s41593-021-01006-0 (PMC8825283; doi:10.1038/s41593-021-01006-0)
Supplement: Supplementary file 1 — (A) Expanded methods and (B) Data collection forms. [file 41593_2021_1006_MOESM1_ESM.pdf]

nature portfolio

https://doi.org/10.1038/s41593-021-01006-0

Supplementary information

Answer ALS, a large-scale resource for sporadic and familial ALS combining clinical and multi-omics data from induced pluripotent cell lines

In the format provided by the authors and unedited

Supplementary Information:

Answer ALS, A Large-Scale Resource for Sporadic and Familial ALS Combining Clinical and Multi-Omics Data from Induced Pluripotent Cell Lines

Correspondence to: jrothstein@jhmi.edu

A. Expanded Methods

Program Process

Overall Design (**Extended Figure 1**).
The overall AALS program, from clinical enrollment to smartphone app data collection, iPS cell line generation, biological data generation, and data storage is outlined in Extended Figure 1 (ClinicalTrials.gov: NCT02574390). Detailed methods for each element of the program are provided below.

Enrollment, Clinical Characterization and Sample Collection

The clinical portions of AALS were coordinated through Johns Hopkins University and Massachusetts General Hospital. The eight enrolling neuromuscular clinics were distributed across the USA and included Johns Hopkins University, Massachusetts General Hospital, Ohio State, Emory University, Washington University, Northwestern University, Cedars Sinai and Texas Neurology (**Supplementary Table 1** and **Extended Data Figure 1**). The clinics were chosen for their geographic distribution, expertise in ALS clinical research and ability to recruit participants rapidly based on prior Northeast ALS Consortium (NEALS) clinical research studies (https://www.neals.org). Enrollment proceeded as planned at a rapid and regular pace (**Figure 1**). To ensure rapid enrollment, clinics were assigned a designated clinic coordinator to work full, half or quarter time, depending on historical rates of monthly enrollment from previous ALS clinical trials. Full clinics were assumed to enroll 5–10 participants/mo, half clinics 2.5–5 participants/mo and quarter clinics 1–3 participant/mo. Of the 861 ALS patients enrolled, over 400 were seen in at least 3 follow-up visits, allowing us to track longitudinal ALSFRS-R total scores

The study was approved by local institutional review boards, and all participants provided written informed consent prior to undergoing any study procedures. Consent was uniform across all sites and included agreement to share data broadly for medical research, in accordance with the mission of the overall program (see Data Access below for specifics). Subjects with sALS, fALS and related motor neuron diseases (referred to as non-ALS motor neuron disease), including those with primary lateral sclerosis, progressive bulbar palsy, and progressive muscular atrophy, along with asymptomatic ALS gene mutation carriers were enrolled in AALS. Age matched control participants without ALS or a family history of ALS were also enrolled. Control subjects were typically spouses and minimal information was collected from each subject with regards to family history, demographics (e.g., ethnic and racial categories) environmental exposures. Demographic comparisons between ALS and controls did not include the non-ALS motor neuron disease subjects.

Upon enrollment, participants were assigned a NeuroGUID (global unique identifier; https://nctu.partners.org/neurobank), used to link participant data within AALS and across studies. Clinical data were entered and stored in a centralized, custom web based electronic data capture system, (NeuroBank). All people over 18 years of age diagnosed with ALS or related motor neuron diseases were eligible to join the study irrespective of disease severity. Control participants were recruited at the same clinics - many were spouses, partners, or caregivers.

Participants were monitored every three months for a year. When possible, the ALS Functional Rating Scale-Revised (ALSFRS-R) was conducted by telephone every three months for another year thereafter. Visits included collection of baseline descriptors followed by measures of ALS progression. Baseline descriptors included: demographics and vital signs, genetic and family history of MND, general medical history, CNS -lability and a brief focused history of environmental exposures. Concomitant medications, and past medical history were collected at enrollment and updated throughout study participation. Measures of ALS progression included: deep tendon reflexes (DTR), Ashworth Spasticity Scale, Hand Held Dynamometry (HHD), ALS Functional Rating Scale – Revised (ALSFRS-R), and pulmonary slow vital capacity (SVC). (See Extended Tables 2,3 and Supplementary data forms). To enhance depth of longitudinal clinical data collection, a secure and HIPAA-compliant smartphone app with a specific focus on motor activity, voice and cognition, was created for home data collection (See **Figure 2** and **Extended Data Figure 2**).

At each in-clinic visit, approximately 50–100 ml of blood was collected from each participant and processed according to methods outlined in the **Supplemental Information**. In addition, at the first visit, whole blood was collected, processed (see **Supplemental Information**) and shipped to Cedar-Sinai for generation of primary peripheral blood mononuclear cell (PBMC)-derived induced pluripotent cell lines.

Biofluid Collection and Processing

At each in-clinic visit, approximately 50–100 ml of blood was collected from each participant. Plasma and serum were processed at the site using standard protocols, divided into 0.5mL aliquots and flash-frozen at -80°C within 30 minutes of collection. In addition, at the first clinic visit ~ 24 ml of blood was collected and processed for PBMC isolation as indicated in the PBMC processing section below. Whole blood was also collected in EDTA tubes and sent to New York Genome Center (NYGC) for DNA extraction and whole genome sequencing. Whole blood collected in EDTA tubes was aliquoted unprocessed and flash-frozen. Cerebrospinal fluid (CSF) was optionally collected, centrifuged at a standard speed to eliminate cellular components, aliquoted and flash-frozen at -80°C. At follow-up visits, plasma and serum were collected, and whole blood, urine and CSF were collected optionally, processed and stored as above. If blood was not collected at the initial visit or quality control problems prevented appropriate analysis or iPSC creation, then blood was collected at a subsequent visit. Serum, plasma, and CSF samples were shipped on dry ice to a centralized biofluid repository at Massachusetts General Hospital (NeuroBank) to be stored at -80°C (See **Extended Table 3**).

Return of Answer ALS Results

To afford medical and ethically appropriate feedback to study participants with ALS were offered the opportunity to receive the results of their whole genome sequencing for five ALS genes (C9orf72, SOD1, FUS, TARDBP, and TBK1), as well 59 genes designated as medically actionable by the American College of Medical Genetics[1], as part of a sub-study, Return of Answer ALS Results (ROAR). ROAR participants completed a separate online consent after enrollment in the parent study. A separate variant interpretation pipeline was applied for the purposes of return of results. Clinical confirmation of each identified variant interpreted as Pathogenic or Likely Pathogenic and genetic counseling by a licensed genetic counselor was offered to all participants in this study.

Answer ALS Smartphone

The app has seven modules designed to gather information about upper limb motor function, respiration, bulbar function and cognition. All seven tasks were repeated weekly. Six modules measured arm function: finger tapping, finger tracing, and phone tilt tracing, each performed using the right and left hand separately (Figure 2a). From speech recordings, we extracted linguistic features to evaluate word diversity and complexity of thought such as semantic similarity, dispersion, and frequency, as recently detailed[2]. The speech module (Figure 2c), consisted of three tasks, rotated weekly to reduce learning effect: 1) Single-Breath Count, in which participants were instructed to draw in a deep breath and count at a measured pace (a surrogate for forced vital capacity)[3] 2) Read Aloud Passage, in which participants read aloud one of four standardized passages from their screen, and 3) Picture Description, in which participants described one of three line-art illustration over 30-120 seconds. For speech task analysis, we used standard acoustic features to assess motor speech degradation such as pitch variations, prosody features, vowel space, vowel quality, noise measurements, mel frequency cepstral coefficients (MFCCs), tremor features and others. We extracted linguistic features to evaluate word diversity and complexity of thought such as semantic similarity, dispersion, and frequency. More details of the methodology have been reported[2]. To evaluate the potential of the tasks to assess different clinical variables used to monitor ALS (e.g. ALSFRS-R, vital capacity, cognitive behavioral screen), the extracted features were entered into three machine learning algorithms (linear, ridge, lasso regression) and validated using 10-fold cross validation.

Limb function tasks included finger tapping, finger tracing, and gyroscope-based tracing that called for the participant to tilt the phone to trace a complex line/figure. Each task was performed using the right and left hand separately, for a total of six individual tasks (**Extended Figure 2**, **Figure 2B,C**).

For the speech module (**Figure 2C**), participants were asked to perform one of 3 tasks: 1) Single-Breath Count, a surrogate for forced vital capacity [3] in which participants were instructed to draw in a deep breath and count at a measured pace; 2) Read Aloud Passage, in which text such as the Bamboo Passage, a standard text used to assess motor speech characteristics, was displayed on the screen and participants were asked to read it aloud, and 3) Free Speech (picture description)(**Figure 2D**), in which participants were presented with a cartoon showing various activities, and asked to record their description of the particular scene over a period spanning 30 seconds to two minutes. These tasks alternated each week, so each one was performed once every 3

weeks. In addition, for the reading and free speech tasks, we alternated between 4 passages and 3 different pictures to reduce learning effects. We analyzed compliance over time, calculating the average number of tasks (total tasks, limb tasks, and bulbar tasks) completed per week of use to evaluate continued engagement with the app.

Depending on the type of task, different features were extracted. To characterize the data obtained from the arm function tasks, errors metrics such as Hausdorff and dynamic time warping distances were calculated. In addition, the number of points acquired by the device during the tracing task was also obtained as a measurement of speed. On the other hand, for speech tasks, we employed standard acoustic features that are known to assess speech degradation such as pitch variations, prosody features, vowel space, vowel quality, noise measurements, mel frequency cepstral coefficients (MFCCs), tremor features, among others. Since one of the speech tasks also evaluates cognition (free speech), recordings were manually transcribed because automated speech-to-text engines were unable to reliably detect dysarthric speech. From the transcripts, we extracted linguistic features to evaluate word diversity and complexity of thought such as semantic similarity, dispersion, and frequency. More details of the methodology have been reported[2]. To evaluate the potential of the tasks to assess different clinical variables used to monitor ALS (e.g. ALSFRS-R, vital capacity, cognitive behavioral screen), the extracted features were entered into different machine learning algorithms (linear, ridge, lasso regression) and validated using a 10-fold cross-validation approach.

IPSC Line Extended Methods:

PBMC Processing: Blood from participants with motor neuron disease and controls was sent to a central iPS cell generation lab (Cedars Sinai) by overnight service where PBMCs were isolated, logged and frozen until iPS cell generation. Fresh blood was collected into 3 8-ml Sodium Citrate BD Vacutainer CPT Tubes (BD, Cat 362761) according to the manufacturer's instructions. Samples were centrifuged at 18-25°C in a horizontal rotor centrifuge for 20 minutes at 1800 RCF within 2 hours of collection. After centrifugation, tubes were inverted to mix the separated buffy coat and plasma layer together. The tubes were then packaged and shipped to Cedars-Sinai via overnight delivery. Once received at Cedars-Sinai, the plasma/buffy coat mixture was collected and centrifuged for 15 mins at 300 RCF. Isolated PBMCs were counted and cryopreserved at 5 million cells per vial in a 1:1 mixture of plasma and CyrostorCS10. Vials were placed in an alcohol freezing container (Mr. Frosty, Nalgene) overnight at -80°C before being transferred to liquid nitrogen for long-term storage.

A total of 1,030 whole-blood samples were collected and sent to Cedars-Sinai for PBMC isolation and cryopreservation. Of the 1,030 samples, 32 were unusable due to issues with sample collection or shipment and 34 samples were redrawn. The average cell count was ~25 million PBMCs per sample with an average cell viability of 91%. In total, the iPS Cell Core at Cedars-Sinai has frozen 2579 vials of PBMCs from 964 unique participants, comprising 860 ALS participants and 104 healthy controls.

iPS Cell line Generation and Reprogramming. iPSCs were generated by reprogramming the cryopreserved and non-expanded PBMCs using a method based on a non-integrating episome. Clones were isolated, expanded and maintained according

to standard feeder-free protocols and characterized extensively as described in **Supplementary Table 6 and Supplementary Table 19**. iPSC lines were generated from ~25 patients per month and stored frozen until they were differentiated (**Extended Figure 3A**). iPSC colonies were maintained on Matrigel-coated 6-well plates (Falcon 353934) at a concentration of 1 mg Matrigel / plate. The Batch Technical Control lines (BTC) through Batch 14 were cultured in mTeSR1 (Stemcell Technologies). All subsequent batches were cultured in mTeSR+. For conciseness, “mTeSR media” will hereafter refer to both mTeSR1 and mTeSR+. Each cell line was thawed and cultured for two to three weeks before passaging for differentiation. Cell lines were differentiated in batches of up to eleven lines.

As of August 2021 ~800 iPS cell lines from participants have been generated. PBMCs were used instead of fibroblasts to limit the potential for genetic defects and facilitate sampling from the large number of patients enrolled in our study. Overall, blood draws are less invasive and carry lower risk for patients than skin biopsies, which improved the overall risk-to-benefit ratio for the study. In addition, it was widely felt that patients would be less likely to consent to a skin biopsy than a blood collection.

Quality Control of iPS Cell Line Generation. Rigorous QC are performed on each Answer ALS iPSC line similar to previously published. [4] For the Answer ALS iPS cell lines, the QC tests for these iPSCs is extensive and includes tests as outlined in a typical assessment shown in **Supplemental Table 6**.

G-band karyotype was performed at multiple passages for each Answer ALS iPSC line at the seed bank under passage 10 and then as well at the working/distribution cell bank at the passage which the iPSC lines are then thawed and differentiated into neurons for the multi-omics studies. With this assessment, we are confident about the genetic integrity of the Answer ALS iPSC repository given that each iPSC line is karyotyped at multiple passages and each time we regenerate a distribution/working cell bank. The G-band karyotype can detect microscopic genomic abnormalities such as inversions, duplications/deletions, balanced and unbalanced translocations, and aneuploidies. Further, the enhanced genetic stability of Answer ALS iPSCs generated from unexpanded blood cells (PBMCs) compared to methods to derived iPSCs from expanded cells (such as fibroblast or LCL), engenders confidence in the genetic stability of the Answer ALS iPSC repository resource generated for this resource.

In addition, cell line authentication is performed at multiple stages (**Supplemental Table 19)**. The STR is performed on the original donor blood/PBMC sample, then performed on the reprogrammed iPSC line and the differentiated neurons.

Generation of iPS Spinal neurons The iPS cells were differentiated into motor neurons according to the direct iPS cell-derived motor neuron (diMNs) protocol, which comprises three main stages (**Extended Figure 3**, **Supplementary Table 6**). As of December 2021, successful motor neuron differentiations from ~ 850 iPS cell lines have been completed by the AALS program. The iPS Cell Core at Cedars-Sinai Medical Center reprograms PBMCs using a non-integrating episomal plasmid method involving 3 stages detailed below.

The direct induced motor neuron (diMN) protocol comprises three stages (**Extended Figure 3**). In stage 1, neural induction and hindbrain specification of iPS cells is achieved by dual inhibition of the SMAD and GSK3β pathways. At the outset of Stage 1, plates from each iPSC cell line were washed with 1 mL DPBS (Corning 21-031-CV)/well and then incubated in 1 mL Accutase (EMD Millipore SCR005)/well for 5 minutes at 37°C. After incubation, 1 mL DPBS/well was added, cells were quickly collected into multiple 15-mL conical tubes (Falcon 352097). Typically, one 6-well plate was collected per tube. Tubes were then centrifuged at 161 g for 2 minutes. The supernatant was aspirated and discarded, and each pellet was re-suspended in 1 mL mTeSR media by gentle trituration using a P-1000 pipette. Once resuspended, all pellets were combined in a final volume of up to 10 mL mTeSR media. Viability and concentration were determined by automated cell counting (Nexcelom Auto 2000). Based on the cell concentration, up to four Matrigel-coated 6-well plates were seeded at a density of 5.0×10^5 cells/well in 2 mL mTeSR media/well. Twenty-four hours following platedown, mTeSR media was exchanged for Stage 1 media (refer to **Supplementary Table 22** for composition). Stage 1 media was exchanged daily until Day 6.

During stage 2, specification of spinal motor neuron precursors is achieved by addition of Shh agonists and retinoic acid. Day 6 began Stage 2 of the differentiation process. For each cell line, all wells were washed with 1 mL DPBS/well and incubated in 1 mL Accutase/well for 5 minutes at 37°C. After incubation, 1 mL DPBS/well was added, cells were quickly collected into multiple 15-mL conical tubes and centrifuged at 161 g for 2 minutes. The supernatant was aspirated and discarded, and each pellet was re-suspended in 1 mL Stage 2 Platedown Media (ST2PD, **Supplementary Table 23**) by gentle trituration using a P-1000 pipette. Once resuspended, all pellets were combined in a final volume of up to 10 mL St2PD. Viability and concentration were then determined by automated cell counting. Based on the cell concentration, up to nine Matrigel-coated 6-well plates were seeded at a density of 7.5×10^5 cells/well in 2 mL St2PD/well. 24 hours following platedown, St2PD was exchanged for Stage 2 media (**Supplementary Table 24**). Stage 2 media was exchanged every other day until day 12.

Maturation of these precursors into neurons with more complex processes and neurites occurs during stage 3 with the addition of neurotrophins and Notch pathway antagonists. Day 12 began Stage 3 of differentiation. For each cell line, Stage 2 media was completely aspirated from all wells and replaced with 2 mL Stage 3 media/well. Stage 3 media (**Supplementary Table 25**) was exchanged every other day until Day 32 of differentiation. During feedings, approximately 75% of old media was aspirated and 2 mL Stage 3/well was added dropwise in a circular manner in order to minimize disruption of the cell monolayer.

On Day 32 of differentiation, cell lines were collected and pelleted as illustrated in Fig. 4. Prior to collection and pelleting, one 6-well plate was selected from each line for brightfield imaging (Molecular Devices ImageExpress Micro). Six regions of interest were captured per well at a magnification of 10X. After imaging, the plates were collected with their respective lines.

An average of two additional iPSC clones per donor were banked at an early passage and reserved as backup. Each iPSC line was banked in an average of 50 vials from multiple passages, including 25 vials at the distribution bank around passage 20.

For each cell line, the number of wells in which the cell monolayer became detached was recorded (**Extended Figure 4**). The mean detachment rate was ~19% (SD +/-0.09). Any of these "lifted" monolayers were not included in the pellet. In addition, four wells/cell line were set aside for short tandem repeat (STR) analysis. For all remaining adherent wells, Stage 3 media was aspirated and replaced with 1 mL DPBS/well. Adherent cell monolayers were manually scraped with a cell scraper (Falcon #353085) and collected using a serological pipette into 15-mL conical tubes. Typically, two 6-well plates were collected per 15-mL conical, and up to eight 6-well plates were collected per line. The 15-mL conicals were centrifuged for 2 minutes at 161 g. The supernatant was then aspirated and discarded, and the pellets were re-suspended in 1 mL DPBS by gentle trituration using a P-1000 pipette. Once resuspended, all pellets were combined in a final volume of approximately 10 mL DPBS and centrifuged for 2 minutes at 161 g. Again, the supernatant was aspirated and discarded. The pellet was then resuspended in 6mL DPBS using a 5-mL serological pipette and aliquoted to six 1.7-mL Eppendorf tubes (1 mL/Eppendorf tube). The Eppendorf tubes were centrifuged for 4 minutes at 161 g, and the supernatants were aspirated and discarded. Four of the Eppendorf tubes were snap-frozen in an ethanol/dry ice slurry and stored at -80˚C until shipment to omics centers for analysis. The remaining two pellets were re-suspended in 1 mL each of CryoStor CS10 (Biolife Solutions #210102) using a P-1000 pipette (typically, 2-4 triturations were sufficient to resuspend the pellets) and each pellet was transferred to an individual cryovial (Thermo Scientific #5000-1020). CryoStor vials were then stored in a Mr. Frosty (Nalgene #5100-0001) at -80˚C for 24 hours, at which time they were transferred to sample boxes and stored at -80˚C until shipment to omics center for processing.

Thus far, ~800 iPS cell lines have been successfully reprogrammed and one clone line banked and characterized per donor. Out of the ~800 unique samples, only 18 lines (~3%) failed reprogramming. When reprogramming fails, a new attempt is made from a blood sample collected at a follow-up clinic visit. In all, the AALS program has created ~30,000 iPS cell vials from all the individual PBMCs reprogrammed thus far

Quality Control of diMNs. As referenced in **Extended Figure 3**, on Day 32 we reserved one 6-well plate from each cell line for immunostaining. We stained each line with the following markers of neuronal differentiation: SMI32(NF-H), TUBB3(TUJ), ISL1, NKX6.1, S100β, and Nestin, as well as DAPI to obtain a total nuclear stain. This protocol generates a mixed population of neurons consisting of ~75% (+/-8%) β_{III}-tubulin (TuJ1) and ~70% (+/-10%) NF-H positive cells, ~19% (+/-6%) Islet-1 and ~34% (+/-9%) Nkx6.1 positive spinal motor neuron, and ~18% (+/13%) S100B positive progenitors 32 days after the onset of differentiation (See **Figure 3**).

To begin the process, each plate was fixed as follows: old media was aspirated and each well washed with 1 mL DPBS +Ca/+Mg (Corning #21-030-CV)/well. Cells were then incubated in 1 mL 4% paraformaldehyde (PFA) solution/well for 10 minutes at room temperature. After incubation, PFA was aspirated and each well carefully washed with 1 mL DPBS (Ca+/Mg+). Finally, 3 mL DPBS (Ca+/Mg+)/well was added and the plates stored at 4˚C until immunostaining.

For immunostaining, each well was blocked for 1 hour at room temperature (5% normal donkey serum (EMD Millipore #S30), 0.2% Triton X-100 (Sigma-Aldrich #T9284) in DPBS (Ca+/Mg+)). Following blocking each well was incubated with primary antibody

(refer to Table# for antibody reference and dilution) for 1 hour at room temperature. Following primary incubation, each well was washed in 1 mL washing solution (0.1% Triton X-100 in DPBS (Ca+/Mg+))/well for 2-3 minutes. Following the wash, secondary antibody (**Supplementary Table 26**) was added to each well and allowed to incubate for 1 hour at room temperature in the dark. Following secondary incubation, each well was washed with 1 mL washing solution/well for 2-3 minutes. Following the wash, each well was incubated with DAPI solution (**Supplementary Table 26**) for 3 minutes at room temperature. Wells were then washed again with 1 mL DPBS (Ca+/Mg+)/well. Finally, 1 mL DPBS (Ca+/Mg+) was added to each well, and the plates were covered with aluminum adhesive film and stored at 4˚C until image acquisition using the ImageExpress Micro system (Molecular Devices). During image acquisition, 64 regions of interest were captured per stained well.

Multi-omics data generation for each iPS cell-derived motor neuron line

At the end of the 32-day differentiation protocol, the spinal neurons were harvested for RNA-Seq, proteomics, or epigenome profiling as detailed below. Whole-genome sequencing was performed on PBMCs. Day 32 was chosen as independent experiments with selected C9orf72 ALS/FTD iPS derived spinal neurons demonstrated phenotypic and molecular change in nuclear pore complex and biology, matching that seen in patient autopsies, by this time point[5]. Thus, at least with that genetic insult, the iPS platform could reproducibly reveal a detectable pathogenic cascade comparable to that seen in patients.

Program Quality Controls: Cell generation batch controls.

Reproducibility of disease signatures from iPS cell-based experiments can be confounded not only by genetic differences between donors (diseased and healthy controls), but also by experimental variability in iPS cell differentiation experiments that can be impacted by variations in differentiation efficiency, cellular composition, transcript and protein abundance. To detect and compensate for such confounders all differentiations were conducted in a single facility and included two key control groups of biological samples: batch differentiation controls (BDC), were differentiated with each batch from the same original line to assess inter-batch variability of iPS cell differentiation to diMNs and Batch Technical controls (BTC), consisting of a single differentiation of the same line was frozen, aliquoted and distributed with each batch to assess technical variability of the omics assay batch runs were performed as detailed in Supplemental Information: Expanded Methods.

Batch technical control (BTC) (Extended Figure 4,5):

The BTC controls for technical variability of a particular 'Omics assay between different batch runs. Briefly, one iPSC line from a healthy donor (CS2AE8iCTR-n6 line) was differentiated in a single large batch at the beginning of the project at the cell generation center (Cedars-Sinai iPSC Core). Multiple biological samples, including snap frozen cell pellets and cryopreserved cell pellets, were prepared to last over a significant period of the data generation component of the project. With each shipment batch, end users at each 'Omics center receive the appropriate BTC biological sample. Each shipment batch comprises three to four batches of iPSC-derived motor

neurons of ALS and healthy control (CTR) subjects, as well as the BTC biological sample, while each differentiation batch comprises 10-15 iPSC lines from different experimental subjects. Since BTC pellets were produced at same time with the same diMNs differentiation standard operating procedure (SOP), a given assay should technically return similar results for any BTC sample across multiple 'Omics batch runs. The BTC thus controls for 'Omics assay-specific variability.

<u>Batch differentiation control (BDC):</u> The BDC controls for inter-batch variability in iPSC differentiation to diMNs.

Briefly, a differentiation batch comprises 10-15 iPSC lines from different ALS and CTR subjects. The same iPSC line used to produce the BTC (CS2AE8iCTR-n6 line) is differentiated in every batch with the other experimental iPSC lines at the cell generation center and is referred to as the Batch Differentiation Control (BDC). This line is thawed, expanded, differentiated, and pelleted in addition to the ALS or healthy control (CTR) lines in each batch. The repeated differentiation of this single line, therefore, serves as a differentiation control, reflecting the intrinsic variability in the iPSC to diMNs differentiation process of the same line across multiple differentiation batches. 'Omics centers receive a BDC sample along with ALS and CTR samples for each differentiation batch. Thus, in addition to the BTC sample, a shipment to the 'Omics center contains multiple BDC samples (one for each differentiation batch included in the shipment).

Data Quality and Batch effects assessments:

RNA-Seq. For the RNA-Seq data, the initial set of 102 samples were processed and passed all quality controls (QC) metrics including RNA integrity (**Extended 9a**), library, and sequencing QC metrics. After read trimming, mapping and expression quantification, we evaluated data composition and quality. To assess data quality and technical batch effects, sample to sample SERE scores (Simple Error Rate Estimate, 0 = identical samples) were generated using gene expression for three groups: the batch differentiation controls (BDCs), batch technical controls (BTCs), and all other samples **(Extended Figure 4,5**). These data show low SERE scores (high gene expression correlation) in the BTC and BDC controls groups, relative to all other samples, indicating minimal to no technical confounders and low batch effects between differentiations. The highest SERE values were found between different individuals. A heatmap of SERE scores between all samples with hierarchical clustering (**Extended Figure 5)** shows that while BTCs form their own cluster, the rest of the samples fall info multiple small clusters with no clear relation to their disease status.

Proteomics. Proteomics data was generated for an initial 66 samples that were processed as a single batch and run sequentially on the MS instrument in blocks of 14. Each block of samples was comprised of case, control, BDC (differential batch control) samples and HEK293 cell control samples (the latter processed on the 96-well digestion plate for use as a sample plate digestion control). The numbers of proteins and peptides quantified for all 66 samples were very consistent (**Extended Figure 4c**), a QC measure which indicates accurate processing consistency and the stability of the intra-batch data acquisitions on the instrument across all samples. The % coefficient of variation (CV) for the proteins quantified were calculated for the BTC and BDC samples **(Extended Figure 4f)**. 80% of the proteins identified in the technical replicates of BTC and BDC samples

across all MS batches have % CV less than or equal to 25%, indicating proteomics data acquisitions between batches were highly reproducible. Individual samples are normalized to the total MS2 spectra intensity across the chromatographic profile of eluting peptides to smooth any inconsistencies in sample loading onto the MS instrument thereby eliminating systemic variation in signal intensities **(Extended Figure 5e**). Finally, in a correlation plot of the protein level data for all 66 samples, we find BTCs and BDCs (both originating from 2AE8 CTR cell line) cluster tightly (**Extended Figure 6c**) indicating minimal drift between the MS batches.

Epigenetics. ATAC-seq data quality was determined according to ENCODE [6]. The distribution of fragment sizes across all samples revealed a clear nucleosome-free region and regular peaks corresponding to n-nucleosomal fractions (**Extended Figure 6**). Mitochondrial DNA contamination was low (mtDNA fraction: 0.07 ± 0.01), and the fraction of reads in called peak regions (FRiP) was within the normal range (mean ± SD = 0.160 ± 0.048), with no difference in quality score between ALS and control samples (p =0.32). As expected, replicates from our batch control line were highly correlated with each other, with batch technical controls (BTC) having an even smaller variation in correlation values compared to batch differentiation controls (BDC) (**Figure 5e**).

Next, we generated a consensus set of peaks present in >10% of samples using DiffBind (**Extended Figure 6**) and characterized transcription factor motif enrichment within these peaks using HOMER[7]. Consistent with our expected cell composition, we observed an overrepresentation of transcription factors implicated in neuronal differentiation, such as Pdx1, Cux2, and the Lhx family (**Figure 6d**). We then obtained a counts matrix of reads mapped to each peak in the consensus peakset across all samples and performed hierarchical clustering using the same approach as the RNA-seq data (**Extended Figure 4,5,6**). Subjects did not cluster by disease status, presence of C9 mutation, sex, or by processing batch.

Whole Genome Methods: Whole-genome sequencing and analysis.

PBMCs were sent by each clinic to The New York Genome Center (NYGC) (https://www.nygenome.org/) for DNA extraction and sample QC. Whole-genome sequencing libraries were prepared, and sequencing was performed on an Illumina NovaSeq 6000 sequencer using 2X150 bp cycles. Sequence data were processed on a NYGC automated pipeline. Sequence runs were assessed and only FASTQ data that were of high quality (exhibiting a 99.9% base call accuracy) were used for processing. Paired-end reads were aligned to the GRCh38 human reference using the Burrows-Wheeler Aligner (BWA-MEMv0.7.8) and processed using the GATK best-practices workflow, which includes marking of duplicate reads by the use of Picard tools (v1.83, http://picard.sourceforge.net), local realignment around indels, and base quality score recalibration (BQSR) via Genome Analysis Toolkit (GATK v3.4.0)[8,9].

We analyzed 830 whole-genome sequences from AALS participants. Of these, 706 were ALS cases, 92 were controls without neurological disease, 16 were individuals diagnosed with a motor neuron disease that is not ALS, 5 had another neurological disorder, 5 were pre-familial ALS (pre-fALS), and 6 had undiagnosed clinical syndromes (**Figure 4; Supplementary Table 10).**

We evaluated pathogenic or likely pathogenic variants reported in ClinVar (C-PLP) for all genes. We observed between 22 and 48 C-PLP variants per individual, with an

average of ~ 34 variants per ALS case and control, similar to what has been reported for Caucasian individuals[10]. The number of rare (<1%) C-PLP variants was approximately 5.2 per ALS case and 5 per control (**Table 2 and Supplementary Table 7**). We also examined pathogenic variants called by Intervar Li, [11] (I-PLP), and predicted damaging variants as called by *in silico* prediction tools (IS-D), which is reported in (**Table 2 and Supplementary Table 8).**

There are 33 genes in which mutations have been associated with ALS[12,13], specifically: *ALS2*[14,15], *ANG*[16-18], *ANXA11*, *ATXN2*, *C21orf2*, *C9orf72*, *CAMTA1*, *CCNF*, *CHCHD10*, *DAO*, *DCTN1*, *FIG4*, *FUS*, *HNRNPA1*, *HNRNPA2B*, *KIF5*, *MATR3*, *MOBP*, *NEK1*, *OPTN*, *PFN1*, *SCFD1*, *SETX*, *SOD1*, *SQSTM1*, *TAF15*, *TARDBP*[19-21], *TBK1*, *TUBA4A*, *UBQLN2*, *UNC13A*, *VAPB* and *VCP*[22]**.** We refer to these as the "33-ALS" genes in the context of the genomic analytics.

The variant calls from NYGC were assessed by examining the actual reads for alignment issues and spot-checking the BAM files for specific variants in IGV determined to be of good quality. The VCFs were converted in to GVCFs, and joint genotyping calling was run using Sentieon v. 201911 (https://www.sentieon.com/), applied variant quality score recalibration (VQSR) was done using GATK v. 3.8 (truth sensitivity level = 99.0), and the files were annotated using Annovar v. 2018Apr16[23] .

For each variant, we also incorporated functional *in silico* predictions from nine programs, including databases such as SIFT[24], PolyPhen2[25], and Mutation Taster[26] and those described in Li et al., 2013[27]. Additional databases were included that assess the variant tolerance of each gene using the RVIS[28] and the Gene Damage Index (GDI)[29] and are adding LoFTool[30]. For variants in genes that are highly expressed in the brain, we incorporated data from the Human Protein Atlas[31] (http://www.proteinatlas.org) and expression data from GTEx portal [32 33], (https://gtexportal.org/home/) for the cortex and spinal cord. Frequency information from three databases on all known variants from ExAC[34], the NHLBI Exome Sequencing Project (ESP)[35], and the 1000 Genomes Project[36].

The NYGC developed an ancestry pipeline that estimates individual genome-wide average ancestries from a set of SNP genotypes using the ADMIXTURE tool, which is a maximum likelihood-based method. The pipeline takes a gVCF generated by Haplotype Caller as input, runs through a series of processing steps in PLINK, and passes the processed PLINK output to ADMIXTURE, which performs ancestry determination. The pipeline estimates ancestries for individual samples at the 1000 Genomes defined "super population" level, which are: AFR: African, AMR: Americas, EAS: East Asian, EUR: European, and SAS: South Asian (http://www.internationalgenome.org/category/population/). Samples from the MXL (Mexican Ancestry from Los Angeles USA) and ASW (Americans of African Ancestry in SW USA) populations were excluded from the reference because they might be putatively admixed. The values range from 0–1 to represent the estimated fraction of each population to which the sample belongs.

Principal component analysis was carried out **(Figure 4d)** to reveal how the Answer ALS samples cluster among various ancestry groups of the 1000 genomes project dataset. "Ad Mixed American" includes Mexicans, Puerto Ricans, Colombians and Peruvians; "African" includes Yoruba, Luhya, Gambian, Mende, Esan, Americans of African Ancestry in SW USA, and African Caribbeans in Barbados; "East Asian" includes

Chinese, Japanese, and Vietnamese; “European” includes Utah residents (CEPH) with Northern and Western European ancestry, Toscans (Italy), Finns, British (England and Scotland), and Iberians (Spain); “South Asian” includes Indian, Pakistani, Bengali, and Sri Lankan. Principal component analysis was used [37,38] to visualize the ancestry background of the AALS cohort and a set of 2504 samples from the 1000 genomes project with well-defined ancestry. We used a set of 10,000 randomly chosen autosomal SNPs (singletons and multiallelic SNPs were removed) that were present in both datasets and removed correlated SNPs by LD-pruning. We implemented randomized PCA[39] using the Python library scikit-allel package[40]

The annotation pipeline incorporated elements from ANNOVAR[41] and generated reports, including genotypes for all samples. These reports are available upon request. The following annotation was used: For genes and exonic variants that have clinical significance, the Clinical Genomic Database (CGD)[42] , the Online Mendelian Inheritance in Man (OMIM)[43], ClinVar[44], and genes listed in the American College of Medical Genetics and Genomics (ACMG)[45] database were incorporated. We also incorporated Intervar, which is based upon the ACMG and AMP standards and guidelines for interpretation of variants[46-49]. This tool uses 18 criteria to prescribe the clinical significance and classifies based on a five-tiered system[50]. To flag ALS genes, ALS gene lists and variants were incorporated from ALSoD[51] (http://alsod.iop.kcl.ac.uk/), a list provided by Dr. Matthew Harms, a gene list from Dr. John Landers, and associations from DisGeNet[52]. Functional predictions were based on *in silico* prediction from nine databases: SIFT[24], PolyPhen2[53-55] (HDIV and HVAR), LRT_Prediction [55], Mutation Taster[26], Mutation assessor [56], FATHMM prediction[57-59], and dbNSFP (RadialSVM_pred and LR_pred)[60-62]. Databases that assess the variant tolerance of each gene using the RVIS[28] and the Gene Damage Index (GDI)[29] were also included, and LoFTool[63] will be incorporated. To identify variants in genes that are highly expressed in the brain, data from the Human Protein Atlas[31] (http://www.proteinatlas.org) and the GTEx portal[64,65], (https://gtexportal.org/home/) for the cortex and spinal cord were used. Frequency information was derived from ExAC[66], the NHLBI Exome Sequencing Project (ESP)[67], and the 1000 Genomes Project[10].

A separate annotation pipeline was developed for variants in intergenic and regulatory regions. Variants are reported relative the closest gene, whether intronic, upstream and downstream (up to 4 KBs from the start and stop of a gene) or in 5‘ and 3‘ UTRs. The annotation was based on RegulomeDB, which annotates variants with known or predicted regulatory elements such as transcription factor binding sites (TFBS), eQTLs, validated functional SNPs and DNase sensitivity[68], with source data from ENCODE[69,70] and GEO[71]. Additional regulatory databases such as Target Scan, an algorithm that uses 14 features to predict and identify microRNA target sites within mRNAs[72], and miRBase[73-75], were also used.

As the predominant ethnicity of the Answer ALS dataset is Caucasian, only the Caucasian samples from the 1000 genomes were used (CEU: Utah Residents with Northern and Western European Ancestry, TSI: Toscani in Italy, FIN: Finnish in Finland, GBR: British in England and Scotland, and IBS: Iberian Population in Spain

RNA Methods

Total RNA was isolated from each sample using the Qiagen RNeasy mini kit. RNA samples for each AALS subject (control or ALS) were entered into an electronic tracking

system and processed at the University of California, Irvine GHTF. RNA QC was conducted using an Agilent Bioanalyzer and Nanodrop. Our primary QC metric for RNA quality is based on RIN values (RNA Integrity Number) ranging from 0-10, 10 being the highest quality RNA. Additionally, we collected QC data on total RNA concentration and 260/280 and 260/230 ratios to evaluate any potential contamination. Only samples with RIN >8 were used for library prep and sequencing. rRNAs were removed and libraries generated using TruSeq Stranded Total RNA library prep kit with Ribo-Zero (Qiagen). RNA-Seq libraries were titrated by qPCR (Kapa), normalized according to size (Agilent Bioanalyzer 2100 High Sensitivity chip). Each cDNA library was then subjected to 100 Illumina (Novaseq 6000) paired end (PE) sequencing cycles to obtain over 50 million PE reads per sample. After sequencing, raw reads were subject to QC measures and reads with quality scores over 20 collected and analyzed. Reads were mapped to the GRCh38 reference genome using Hisat2, QCed, and gene expression quantified with featureCounts[76] and differential expression were quantified using DESeq2[77]. Normalized and transformed count data were also used for exploratory analysis and differentially expressed (DE) genes (FDR <0.1) were analyzed with commercial and open-source pathway and network analysis tools, including Ingenuity Pathway Analysis (IPA), GSEA, GOrilla, Cytoscape, and other tools to identify transcriptional regulators, predict epigenomic changes, and determine potential effects on downstream pathways and cellular functions.

ATAC seq Methods

We used the Assay for Transposase-Accessible Chromatin using Sequencing (ATAC-Seq) to assess chromatin accessibility and identify functional regulatory sites involved in driving transcriptional changes associated with ALS. ATAC-Seq detects open chromatin sites genome-wide and maps transcription factor binding events in global regulatory elements without needing prior information about which proteins are present. ATAC-seq sample prep, sequencing, and peak generation was carried out by Diagenode Inc as further described[78]. Briefly, cells were lysed in ATAC-seq resuspension buffer (RSB; 10 mM Tris-HCl pH 7.4, 10 mM NaCl, 3 mM $MgCl_2$, protease inhibitors) with a mixture of detergents (0.1% Tween-20, 0.1% NP-40, 0.01% digitonin) on ice for 5 min. The lysis reaction was washed out with additional ATAC-RSB containing 0.1% Tween-20 and inverted to mix. 50K nuclei were collected and centrifuged at 450 rcf for 5 min at 4 °C. The pellet was re-suspended in 50 µl of transposition mixture (25 µl 2X Illumina Tagment DNA Buffer, 2.5 µl Illumina Tagment DNA Enzyme, 16.5 µl PBS, 0.5 µl 1% digitonin, 0.5 µl 10% Tween-20, 5 µl water). The transposition reaction was incubated at 37°C for 30 min followed by DNA purification. An initial PCR amplification was performed on the tagmented DNA using Nextera indexing primers (Illumina). Real-time qPCR was run with a fraction of the tagmented DNA to determine the number of additional PCR cycles needed and a final PCR amplification was performed. Size selection was done using AMPure XP beads (Beckman Coulter) to remove small, unwanted fragments (<100 bp). The final libraries were sequenced using the Illumina NextSeq platform (paired-end, 75nt kit). All samples passed quality control checks that included morphological evaluation of nuclei, fluorescence-based electrophoresis of libraries to assess size distribution, and real-time qPCR to assess the enrichment of open-chromatin sites. The quality of the sequencing was assessed using FastQC and the reads were aligned to GRCh38 genome

build using Bowtie2. We identified open chromatin regions separately for each sample using the peak-calling software MACS2[79] and determined differentially open sites using DESeq2 (FDR<0.1). Peaks were assigned to unique genes using the default HOMER[7] parameters, and gene ontology analysis was performed using GOrilla[80].

Proteome Methods

Whole proteome extracts from frozen diMNs were digested with trypsin and LysC and subjected to acquisition on the SCIEX 6600 as detailed below. Snap frozen cell pellets were stored at -80°C and transferred to the CSMC proteomics lab on dry ice where it was stored at -80°C until use. Samples were lyophilized and aliquoted into 600 ul polystyrene microcentrifuge tubes containing lysis buffer (6M Urea, 1 mM DTT in 1.5 M NH_4HCO_3). Sample was sonicated (QSonica Q800R1) by alternating 10 seconds on and 10 seconds off at 70% amplitude while rotating in a 4°C water bath until the solution was homogenized (~20 mins). Samples were centrifuges and the protein concentration determined on the supernatant according to manufactures' instructions (Pierce™ BCA Protein Assay Kit). 200 ug of each sample was transferred to a 96 well plate in aliquots and processed on the Biomek i7 Automated workstation (Beckman Coulter) as outlined previously. Briefly, samples underwent the following: reduction of disulfide bonds in 3 mM TCEP, alkylated in 5 mM IAA. Addition of Beta-galactosidase at 2 ug and protein digestion in solution using equal molar Trypsin and LysC enzyme mixture (Promega, product #: V5111) at 1:40 enzyme to protein ratio under optimized digestion conditions (4 hours at 37°C). Digested proteins were desalted on a 5 mg Oasis HLB 96 well plate (Waters; product #: 186000309) and eluted in 50% acetonitrile. Samples were dried to completion using a speed-vac system and stored at -80°C until MS analysis. For MS analysis, digested peptides were resuspended in 0.1% FA and analyzed on a 6600 Triple TOF (Sciex) in data-independent acquisition (DIA) mode and on the 6600 Triple TOF (Sciex) for data dependent acquisition (DDA) mode. Specifically, samples were acquired in DDA mode for ion library generation and in DIA mode over 100 variable windows similar to previously described acquisition protocols [81,82].

DDA data was used for the generation of a sample specific peptide ion library. DDA files were run through Trans Proteome Pipeline (TPP) using a human canonical FASTA file (Uniprot). A consensus peptide library with decoys was generated and used to quantify ions identified in DIA data files. Previously described DDA library build principles [83] were utilized to generate a cell-specific library, which allowed for greater accuracy in matching DIA data to the DDA library during OpenSWATH, as indicated by higher d-scores in PyProphet. Differential protein expression between ALS and control samples analyzed was calculated using mapDIA. [84]

DIA data files were analyzed with OpenSWATH pipeline against the sample specific peptide ion library generated. Protein level quantitation is calculated by summing transition level intensities for all the proteotypic peptides identified. Differential protein expression between ALS and control samples analyzed was calculated using mapDIA.

Imaging Methods

Longitudinal single cell imaging and analysis.

Differentiated iMNs from a subset of the AALS iPSC lines were plated on 96-well plates for longitudinal single cell imaging with robotic microscopy as previously

described[85-94]. At day 25, cells were transduced with expression marker plasmids such as synapsin::EGFP[95] to visualize cell morphology and viability. After transduction cells were imaged in an automated fashion with robotic microscopy once per day for 10–14 days. A fiducial mark from the plate was imaged during the first imaging run and then used each time thereafter to register the position of the plate and align it to its initial position. This enabled the system to collect images of the same microscope fields over the course of the experiment and to identify and track individual iMNs. Images of different microscope fields from the same well were stitched together into montages, and montages of the same well collected at different time points were organized into composite files in temporal order. Some image analysis was performed in a computational pipeline constructed within the open-source program Galaxy, to identify and track individual cells and perform survival analysis and other morphological measurements.

Statistics.

The overall study design with regard to the size of the patient and controls populations was based on clinical considerations, e.g., numbers of patient with various genetic forms of ALS as well as sporadic ALS, rather than specific statistical considerations. Multiple statistical tests were employed to assess population and group differences. Where appropriate, descriptive statistics were used (N, mean, standard error of the mean, median, minimum, and maximum values). For human demographics, T-test was used for continuous variables and chi square test for categorical ones. Other statistical analyses include Pearson R and Spearman correlation matrix.

For longitudinal imaging studies, the control and SOD1 ALS cell lines were assessed across several experiments and statistically modeled using Cox Mixed Effects model. The experiment-to-experiment variability, the image-to-image variability within each experiment and the individual cell lines themselves were modeled as random effects. The hazard ratio of neuron survival of disease lines versus control lines was estimated as a fixed effect. The design of the experiment was such that in no situation the experiment effect was entirely confounded by the cell-line effect. For gene expression, a negative binomial distribution-based model and Wald test implemented in DESeq2 was employed. For splicing analysis, a hierarchical model and likelihood ratio test implemented in rMATs was used while for for RBP motif analysis, a Wilcoxon rank sum test implemented in rMAPs was used.

Data Portal

Data Storage and Data Integration/Analytics

Answer ALS was designed to be an "open source" program. All of the clinical data sets, the various omics results, including whole genome, proteome, transcriptome and epigenome along with the data integration have been posted to a portal for data sharing and crowd sourcing (https://data.answerals.org/; **Supplementary Table 3**). Data are available for download to all academic and commercial researchers. A required data use agreement provides assurance that users will not attempt to violate the research participants NeuroGUID privacy, as well as share or sell the raw data without Answer ALS permissions. There are no intellectual property restrictions on the use of the data.

Web-based analytics. We have included online analytics for the many ALS researchers who will neither need nor want to download the full dataset. The current set

of tools available at http://data.answerals.org/analyze allow users to select genes/pathways of interest and visualize them using braid maps, heat maps, volcano plots, bar charts or networks (**Figure 4).**

The data portal provides users with information about the AALS program, the data, relevant terminology and data release notes. Users can download a metadata package associated with each versioned release. This versioned package contains comprehensive clinical, iPSC and inventory metadata. In addition, processes for enrolling patients, producing iPSC lines and performing Whole Genome Sequencing (WGS) are explained with links provided to the relevant facilities/institutions. Explanations for sample collection and analysis of Epigenomics, Proteomics and Transcriptomics data are available. Finally, precise definitions are provided for our data levels, which are ways to stratify all the various omics data coming from our analyses (**Supplementary Table 20**).

Data Dissemination

The Answer ALS Data Portal (http://data.answerals.org/; **Supplementary Table 3**) provides all raw and processed data including longitudinal clinical data and biological data generated by the AALS program and provides easy visualization/access to the metadata, data and biosamples released. The portal provides an overview of the data release notes, assays, data level descriptions and links to sites for viewing cell lines/biosamples associated with the program. The website allows browsing of all available metadata (using filter and text search functions), the option to download all data and metadata or a filtered subset and links to obtain individual iPS cell lines from the Cedars-Sinai Biomanufacturing Center.

Users interested in downloading datasets are required to submit an online form, acknowledge data use parameters and return a signed Data Use Agreement (DUA). These measures serve to protect our enrolled participants' privacy in compliance with HIPAA. In addition, results generated using AALS have the possibility of being shared for collaborative and open science purposes.

In addition, the data portal provides the user a means to access metadata, data and biosamples. The portal provides visual tools allowing researchers to find data by sample and participant features. Each sample is described by its omics assay, experiment type, sample name and subject ID. Samples can be removed from the visualization based on filter selection (e.g. filtering for only male patients). Once filters are selected, the user can download metadata or data associated with the filtered samples. For example, a researcher can retrieve metadata and data for patients who are older than 50 and have a known C9ORF72 mutation. Users are also able to find iPSC lines to order from Cedars-Sinai Biomanufacturing Center using the same filtering tools.

Data Organization and Naming.

The organization and naming of our data, regardless of data type, is an essential component of the program. We organize and name data products in a unified and systematic manner to allow a smooth end-user experience. A key component to data organization in our program is the usage of data levels. Data levels are a categorization schema to group similar types of omics data products together. **Supplementary Table 20** gives specific details on the data levels we have defined. **Supplementary Table 21**

describes examples of these data levels in action with each experimental assay our program collects.

The AALS data program prefixes all data products in a systematic manner. The prefix consists of the following components: whether the sample is from a disease patient or healthy control patient, the de-identified patient GUID, the sample vial ID and the assay type abbreviation. An example of this is the raw Transcriptomics FASTQ file CASE-NEUAA599TMX-5310-T_P10_1.fastq.gz. The first underscore separates the prefix from any supplementary file information allowing for easy tokenization. This nomenclature is applied consistently to all metadata and data files making it easy to establish relationships with a single study participant.

References: Expanded Methods

1 Kalia, S. S. *et al.* Recommendations for reporting of secondary findings in clinical exome and genome sequencing, 2016 update (ACMG SF v2.0): a policy statement of the American College of Medical Genetics and Genomics. *Genetics in medicine : official journal of the American College of Medical Genetics* **19**, 249-255, doi:10.1038/gim.2016.190 (2017)

2 Agurto, C. *et al.* Analyzing progression of motor and speech impairment in ALS. *Annu Int Conf IEEE Eng Med Biol Soc* **2019**, 6097-6102, doi:10.1109/EMBC.2019.8857300 (2019)

3 Elsheikh, B. *et al.* Correlation of single-breath count test and neck flexor muscle strength with spirometry in myasthenia gravis. *Muscle Nerve* **53**, 134-136, doi:10.1002/mus.24929 (2016) PMC4715713

4 Toombs, J. *et al.* Generation of twenty four induced pluripotent stem cell lines from twenty four members of the Lothian Birth Cohort 1936. *Stem cell research* **46**, 101851, doi:10.1016/j.scr.2020.101851 (2020) PMC7347008

5 Coyne, A. N. *et al.* G4C2 Repeat RNA Initiates a POM121-Mediated Reduction in Specific Nucleoporins in C9orf72 ALS/FTD. *Neuron* **107**, 1124-1140 e1111, doi:10.1016/j.neuron.2020.06.027 (2020)

6 Consortium, E. P. An integrated encyclopedia of DNA elements in the human genome. *Nature* **489**, 57-74, doi:10.1038/nature11247 (2012) PMC3439153

7 Heinz, S. *et al.* Simple combinations of lineage-determining transcription factors prime cis-regulatory elements required for macrophage and B cell identities. *Mol Cell* **38**, 576-589, doi:10.1016/j.molcel.2010.05.004 (2010) PMC2898526

8 McKenna, A. *et al.* The Genome Analysis Toolkit: A MapReduce framework for analyzing next-generation DNA sequencing data. *Genome Res* **20**, 1297–1303, doi:10.1101/gr.107524.110 (2010) PMC2928508

9 DePristo, M. A. *et al.* A framework for variation discovery and genotyping using next-generation DNA sequencing data. *Nat Genet* **43**, 491-498, doi:10.1038/ng.806 (2011) PMC3083463

10 Genomes Project, C. *et al.* A global reference for human genetic variation. *Nature* **526**, 68-74, doi:10.1038/nature15393 (2015) PMC4750478

11 Li, M. X., Gui, H. S., Kwan, J. S., Bao, S. Y. & Sham, P. C. A comprehensive framework for prioritizing variants in exome sequencing studies of Mendelian diseases. *Nucleic acids research* **40**, e53, doi:10.1093/nar/gkr1257 (2012) 3326332

12 Alsultan, A. A., Waller, R., Heath, P. R. & Kirby, J. The genetics of amyotrophic lateral sclerosis: Current insights. *Degener. Neurol. Neuromuscul. Dis.* **6**, 49–64, doi:10.2147/DNND.S84956 (2016) PMC6053097

13 Mejzini, R. *et al.* ALS Genetics, Mechanisms, and Therapeutics: Where Are We Now? *Front Neurosci* **13**, 1310, doi:10.3389/fnins.2019.01310 (2019) PMC6909825

14 Sato, K. *et al.* Altered oligomeric states in pathogenic ALS2 variants associated with juvenile motor neuron diseases cause loss of ALS2-mediated endosomal function. *J Biol Chem* **293**, 17135-17153, doi:10.1074/jbc.RA118.003849 (2018) PMC6222102

15 Hadano, S. *et al.* A gene encoding a putative GTPase regulator is mutated in familial amyotrophic lateral sclerosis 2. *Nat Genet* **29**, 166-173, doi:10.1038/ng1001-166 ng1001-166 [pii] (2001)

16 Greenway, M. J. *et al.* ANG mutations segregate with familial and 'sporadic' amyotrophic lateral sclerosis. *Nat Genet* **38**, 411-413, doi:ng1742 [pii]

10.1038/ng1742 (2006)

17 Wu, D. *et al.* Angiogenin loss-of-function mutations in amyotrophic lateral sclerosis. *Ann Neurol* **62**, 609-617, doi:10.1002/ana.21221 (2007) PMC2776820

18 Bradshaw, W. J. *et al.* Structural insights into human angiogenin variants implicated in Parkinson's disease and Amyotrophic Lateral Sclerosis. *Sci Rep* **7**, 41996, doi:10.1038/srep41996 (2017) PMC5296752

19 Rutherford, N. J. *et al.* Novel mutations in TARDBP (TDP-43) in patients with familial amyotrophic lateral sclerosis. *PLoS Genet* **4**, e1000193, doi:10.1371/journal.pgen.1000193 (2008) 2527686

20 Sreedharan, J. *et al.* TDP-43 mutations in familial and sporadic amyotrophic lateral sclerosis. *Science* **319**, 1668-1672, doi:1154584 [pii]10.1126/science.1154584 (2008)

21 Mackenzie, I. R. & Rademakers, R. The role of transactive response DNA-binding protein-43 in amyotrophic lateral sclerosis and frontotemporal dementia. *Curr Opin Neurol* **21**, 693-700, doi:10.1097/WCO.0b013e3283168d1d (2008) PMC2869081

22 Koppers, M. *et al.* VCP mutations in familial and sporadic amyotrophic lateral sclerosis. *Neurobiol Aging* **33**, 837 e837-813, doi:10.1016/j.neurobiolaging.2011.10.006 (2012)

23 Wang, K., Li, M. & Hakonarson, H. ANNOVAR: functional annotation of genetic variants from high-throughput sequencing data. *Nucleic Acids Res* **38**, e164, doi:10.1093/nar/gkq603 (2010) PMC2938201

24 Sim, N. L. *et al.* SIFT web server: predicting effects of amino acid substitutions on proteins. *Nucleic Acids Res* **40**, W452-457, doi:10.1093/nar/gks539 (2012) PMC3394338

25 Chun, S. & Fay, J. C. Identification of deleterious mutations within three human genomes. *Genome research* **19**, 1553-1561, doi:10.1101/gr.092619.109 (2009) 2752137

26 Schwarz, J. M., Rodelsperger, C., Schuelke, M. & Seelow, D. MutationTaster evaluates disease-causing potential of sequence alterations. *Nat Methods* **7**, 575-576, doi:10.1038/nmeth0810-575 (2010)

27 Li, M. X. *et al.* Predicting mendelian disease-causing non-synonymous single nucleotide variants in exome sequencing studies. *PLoS genetics* **9**, e1003143, doi:10.1371/journal.pgen.1003143 (2013) 3547823

28 Petrovski, S., Wang, Q., Heinzen, E. L., Allen, A. S. & Goldstein, D. B. Genic intolerance to functional variation and the interpretation of personal genomes. *PLoS genetics* **9**, e1003709, doi:10.1371/journal.pgen.1003709 (2013) 3749936

29 Itan, Y. *et al.* The human gene damage index as a gene-level approach to prioritizing exome variants. *Proceedings of the National Academy of Sciences of the United States of America* **112**, 13615-13620, doi:10.1073/pnas.1518646112 (2015) PMC4640721

30 Fadista, J., Oskolkov, N., Hansson, O. & Groop, L. LoFtool: a gene intolerance score based on loss-of-function variants in 60 706 individuals. *Bioinformatics* **33**, 471-474, doi:10.1093/bioinformatics/btv602 (2017)

31 Uhlen, M. *et al.* Proteomics. Tissue-based map of the human proteome. *Science* **347**, 1260419, doi:10.1126/science.1260419 (2015)

32 Consortium, G. T. Human genomics. The Genotype-Tissue Expression (GTEx) pilot analysis: multitissue gene regulation in humans. *Science* **348**, 648-660, doi:10.1126/science.1262110 (2015) PMC4547484

33 Consortium, G. T. The Genotype-Tissue Expression (GTEx) project. *Nat Genet* **45**, 580-585, doi:10.1038/ng.2653 (2013) PMC4010069

34 Lek, M. *et al.* Analysis of protein-coding genetic variation in 60,706 humans. *Nature* **536**, 285-291, doi:10.1038/nature19057 (2016) 5018207

35 Tennessen, J. A. *et al.* Evolution and functional impact of rare coding variation from deep sequencing of human exomes. *Science* **337**, 64-69, doi:10.1126/science.1219240 (2012) 3708544

36 Auton, A. *et al.* A global reference for human genetic variation. *Nature* **526**, 68-74, doi:10.1038/nature15393 (2015) 4750478

37 Patterson, N., Price, A. L. & Reich, D. Population structure and eigenanalysis. *PLoS genetics* **2**, e190, doi:10.1371/journal.pgen.0020190 (2006) PMC1713260

38 McVean, G. A genealogical interpretation of principal components analysis. *PLoS genetics* **5**, e1000686, doi:10.1371/journal.pgen.1000686 (2009) PMC2757795

39 Galinsky, K. J. *et al.* Fast Principal-Component Analysis Reveals Convergent Evolution of ADH1B in Europe and East Asia. *Am J Hum Genet* **98**, 456-472, doi:10.1016/j.ajhg.2015.12.022 (2016) PMC4827102

40 Fabian Pedregosa, G. V., Alexandre Gramfort, Vincent Michel, Bertrand Thirion, Olivier Grisel, Mathieu Blondel, Andreas Müller, Joel Nothman, Gilles Louppe, Peter Prettenhofer, Ron Weiss, Vincent Dubourg, Jake Vanderplas, Alexandre Passos, David Cournapeau, Matthieu Brucher, Matthieu Perrot, Édouard Duchesnay. Scikit-learn: Machine Learning in Python. *Journal of Machine Learning Research* doi:arXiv:1201.0490 (2012)

41 Wang, K., Li, M. & Hakonarson, H. ANNOVAR: Functional annotation of genetic variants from high-throughput sequencing data. *Nucleic Acids Res.* **38**, e164 (2010) 2938201

42 Solomon, B. D., Nguyen, A. D., Bear, K. A. & Wolfsberg, T. G. Clinical genomic database. *Proceedings of the National Academy of Sciences of the United States of America* **110**, 9851-9855, doi:10.1073/pnas.1302575110 (2013) PMC3683745

43 Amberger, J. S., Bocchini, C. A., Schiettecatte, F., Scott, A. F. & Hamosh, A. OMIM.org: Online Mendelian Inheritance in Man (OMIM(R)), an online catalog of human genes and

genetic disorders. *Nucleic Acids Res* **43**, D789-798, doi:10.1093/nar/gku1205 (2015) PMC4383985
44 Landrum, M. J. *et al.* ClinVar: public archive of interpretations of clinically relevant variants. *Nucleic Acids Res* **44**, D862-868, doi:10.1093/nar/gkv1222 (2016) PMC4702865
45 Green, R. C. *et al.* ACMG recommendations for reporting of incidental findings in clinical exome and genome sequencing. *Genet Med* **15**, 565-574, doi:10.1038/gim.2013.73 (2013) PMC3727274
46 Richards, C. S. *et al.* ACMG recommendations for standards for interpretation and reporting of sequence variations: Revisions 2007. *Genet Med* **10**, 294-300, doi:10.1097/GIM.0b013e31816b5cae (2008)
47 Kazazian, J., Boehm, C. D., & Seltzer, W. K. . ACMG recommendations for standards for interpretation of sequence variations. Genetics in Medicine. *Genetics in Medicine,* **2(5)**, 302-303., doi:doi.org/10.1097/00125817-200009000-00009 (2000)
48 Richards, S. *et al.* Standards and guidelines for the interpretation of sequence variants: a joint consensus recommendation of the American College of Medical Genetics and Genomics and the Association for Molecular Pathology. *Genet Med* **17**, 405-424, doi:10.1038/gim.2015.30 (2015) PMC4544753
49 Li, Q. & Wang, K. InterVar: Clinical Interpretation of Genetic Variants by the 2015 ACMG-AMP Guidelines. *Am J Hum Genet* **100**, 267-280, doi:10.1016/j.ajhg.2017.01.004 (2017) PMC5294755
50 Farrer, L. A. *et al.* Effects of age, sex, and ethnicity on the association between apolipoprotein E genotype and Alzheimer disease. A meta-analysis. APOE and Alzheimer Disease Meta Analysis Consortium. *JAMA* **278**, 1349-1356 (1997)
51 Abel, O. *et al.* Development of a smartphone app for a genetics website: The amyotrophic lateral sclerosis online genetics database (ALSoD). *JMIR Mhealth Uhealth* **1**, e18, doi:10.2196/mhealth.2706 (2013) PMC4114449
52 Pinero, J. *et al.* DisGeNET: a comprehensive platform integrating information on human disease-associated genes and variants. *Nucleic Acids Res* **45**, D833-D839, doi:10.1093/nar/gkw943 (2017) PMC5210640
53 Adzhubei, I., Jordan, D. M. & Sunyaev, S. R. Predicting functional effect of human missense mutations using PolyPhen-2. *Curr Protoc Hum Genet* **Chapter 7**, Unit7 20, doi:10.1002/0471142905.hg0720s76 (2013) PMC4480630
54 Ramensky, V., Bork, P. & Sunyaev, S. Human non-synonymous SNPs: server and survey. *Nucleic Acids Res* **30**, 3894-3900, doi:10.1093/nar/gkf493 (2002) PMC137415
55 Sunyaev, S. R. *et al.* PSIC: profile extraction from sequence alignments with position-specific counts of independent observations. *Protein Eng* **12**, 387-394, doi:10.1093/protein/12.5.387 (1999)
56 Reva, B., Antipin, Y. & Sander, C. Predicting the functional impact of protein mutations: application to cancer genomics. *Nucleic Acids Res* **39**, e118, doi:10.1093/nar/gkr407 (2011) PMC3177186
57 Shihab, H. A. *et al.* Predicting the functional, molecular, and phenotypic consequences of amino acid substitutions using hidden Markov models. *Hum Mutat* **34**, 57-65, doi:10.1002/humu.22225 (2013) PMC3558800

58 Shihab, H. A., Gough, J., Cooper, D. N., Day, I. N. & Gaunt, T. R. Predicting the functional consequences of cancer-associated amino acid substitutions. *Bioinformatics* **29**, 1504-1510, doi:10.1093/bioinformatics/btt182 (2013) PMC3673218

59 Shihab, H. A. *et al.* Ranking non-synonymous single nucleotide polymorphisms based on disease concepts. *Hum Genomics* **8**, 11, doi:10.1186/1479-7364-8-11 (2014) PMC4083756

60 Liu, X., Jian, X. & Boerwinkle, E. dbNSFP: a lightweight database of human nonsynonymous SNPs and their functional predictions. *Hum Mutat* **32**, 894-899, doi:10.1002/humu.21517 (2011) PMC3145015

61 Liu, X., Jian, X. & Boerwinkle, E. dbNSFP v2.0: a database of human non-synonymous SNVs and their functional predictions and annotations. *Hum Mutat* **34**, E2393-2402, doi:10.1002/humu.22376 (2013) PMC4109890

62 Liu, X., Wu, C., Li, C. & Boerwinkle, E. dbNSFP v3.0: A One-Stop Database of Functional Predictions and Annotations for Human Nonsynonymous and Splice-Site SNVs. *Hum Mutat* **37**, 235-241, doi:10.1002/humu.22932 (2016) PMC4752381

63 Fadista, J., Oskolkov, N., Hansson, O. & Groop, L. LoFtool: A gene intolerance score based on loss-of-function variants in 60 706 individuals. *Bioinformatics* **33**, 471–474, doi:10.1093/bioinformatics/btv602 (2017)

64 G. TEx Consortium. The Genotype-Tissue Expression (GTEx) project. *Nat Genet* **45**, 580-585, doi:10.1038/ng.2653 (2013) PMC4010069

65 G. TEx Consortium. Human genomics. The genotype-tissue expression (GTEx) pilot analysis: Multitissue gene regulation in humans. *Science (New York, N.Y.)* **348**, 648–660, doi:10.1126/science.1262110 (2015) PMC4547484

66 Lek, M. *et al.* Analysis of protein-coding genetic variation in 60,706 humans. *Nature* **536**, 285-291, doi:10.1038/nature19057 (2016) PMC5018207

67 Tennessen, J. A. *et al.* Evolution and functional impact of rare coding variation from deep sequencing of human exomes. *Science* **337**, 64-69, doi:10.1126/science.1219240 (2012) PMC3708544

68 Boyle, A. P. *et al.* Annotation of functional variation in personal genomes using RegulomeDB. *Genome Res.* **22**, 1790–1797, doi:10.1101/gr.137323.112 (2012) PMC3431494

69 Encode Project Consortium. The ENCODE (ENCyclopedia Of DNA Elements) Project. *Science (New York, N.Y.)* **306**, 636-640, doi:10.1126/science.1105136 (2004)

70 Encode Project Consortium. An integrated encyclopedia of DNA elements in the human genome. *Nature* **489**, 57-74, doi:10.1038/nature11247 (2012) PMC3439153

71 Barrett, T. *et al.* NCBI GEO: Archive for high-throughput functional genomic data. *Nucleic Acids Res* **37**, D885-890, doi:10.1093/nar/gkn764 (2009) PMC2686538

72 Agarwal, V., Bell, G. W., Nam, J. W. & Bartel, D. P. Predicting effective microRNA target sites in mammalian mRNAs. *Elife* **4**, doi:10.7554/eLife.05005 (2015) PMC4532895

73 Griffiths-Jones, S. The microRNA Registry. *Nucleic Acids Res* **32**, D109-111, doi:10.1093/nar/gkh023 (2004) PMC308757

74 Griffiths-Jones, S., Grocock, R. J., van Dongen, S., Bateman, A. & Enright, A. J. miRBase: MicroRNA sequences, targets and gene nomenclature. *Nucleic. Acids Res.* **34**, D140–144, doi:10.1093/nar/gkj112 (2006) PMC1347474

75 Griffiths-Jones, S., Saini, H. K., van Dongen, S. & Enright, A. J. miRBase: Tools for microRNA genomics. *Nucleic. Acids Res.* **36**, D154–158, doi:10.1093/nar/gkm952 (2008) PMC2238936
76 Anders, S., Pyl, P. T. & Huber, W. HTSeq--a Python framework to work with high-throughput sequencing data. *Bioinformatics* **31**, 166-169, doi:10.1093/bioinformatics/btu638 (2015) 4287950
77 Love, M. I., Huber, W. & Anders, S. Moderated estimation of fold change and dispersion for RNA-seq data with DESeq2. *Genome biology* **15**, 550, doi:10.1186/s13059-014-0550-8 (2014) 4302049
78 Milani, P. *et al.* Cell freezing protocol suitable for ATAC-Seq on motor neurons derived from human induced pluripotent stem cells. *Sci Rep* **6**, 25474, doi:10.1038/srep25474 (2016) PMC4857123
79 Zhang, Y. *et al.* Model-based analysis of ChIP-Seq (MACS). *Genome biology* **9**, R137, doi:10.1186/gb-2008-9-9-r137 (2008) 2592715
80 Eden, E., Navon, R., Steinfeld, I., Lipson, D. & Yakhini, Z. GOrilla: a tool for discovery and visualization of enriched GO terms in ranked gene lists. *BMC bioinformatics* **10**, 48, doi:10.1186/1471-2105-10-48 (2009) 2644678
81 Holewinski, R. J., Parker, S. J., Matlock, A. D., Venkatraman, V. & Van Eyk, J. E. Methods for SWATH: Data Independent Acquisition on TripleTOF Mass Spectrometers. *Methods Mol Biol* **1410**, 265-279, doi:10.1007/978-1-4939-3524-6_16 (2016)
82 Kirk, J. A. *et al.* Pacemaker-induced transient asynchrony suppresses heart failure progression. *Science translational medicine* **7**, 319ra207, doi:10.1126/scitranslmed.aad2899 (2015) 4858435
83 Parker, S. J., Venkatraman, V. & Van Eyk, J. E. Effect of peptide assay library size and composition in targeted data-independent acquisition-MS analyses. *Proteomics* **16**, 2221-2237, doi:10.1002/pmic.201600007 (2016)
84 Teo, G. *et al.* mapDIA: Preprocessing and statistical analysis of quantitative proteomics data from data independent acquisition mass spectrometry. *J Proteomics* **129**, 108-120, doi:10.1016/j.jprot.2015.09.013 (2015) PMC4630088
85 Arrasate, M. & Finkbeiner, S. Automated microscope system for determining factors that predict neuronal fate. *Proc. Natl. Acad. Sci. U. S. A.* **102**, 3840–3845 (2005) PMCID: PMC553329
86 Arrasate, M. & Finkbeiner, S. Protein aggregates in Huntington's disease. *Experimental neurology* **238**, 1–11 (2012) PMC3909772
87 Arrasate, M., Mitra, S., Schweitzer, E. S., Segal, M. R. & Finkbeiner, S. Inclusion body formation reduces levels of mutant huntingtin and the risk of neuronal death. *Nature* **431**, 805–810 (2004) PMC15483602
88 Miller, J. *et al.* Identifying polyglutamine protein species *in situ* that best predict neurodegeneration. *Nat. Chem. Biol.* **7**, 925–934 (2011) PMCID: PMC3271120
89 Mitra, S., Tsvetkov, A. S. & Finkbeiner, S. Single neuron ubiquitin-proteasome dynamics accompanying inclusion body formation in Huntington disease. *J. Biol. Chem.* **284**, 4398–4403 (2009b) PMCID: PMC2640959

90 Tsvetkov, A. S. *et al.* Proteostasis of polyglutamine varies among neurons and predicts neurodegeneration. *Nat. Chem. Biol.* **9**, 586–592, doi:10.1038/nchembio.1308 (2013) PMC3900497
91 HD iPSC Consortium *et al.* Induced pluripotent stem cells from patients with Huntington's disease show CAG-repeat-expansion-associated phenotypes. *Cell Stem Cell* **11**, 264–278 (2012) PMC3804072
92 Barmada, S. J. *et al.* Cytoplasmic mislocalization of TDP-43 is toxic to neurons and enhanced by a mutation associated with familial amyotrophic lateral sclerosis. *J. Neurosci.* **30**, 639–649 (2010b) PMC2821110
93 Bilican, B. *et al.* Mutant induced pluripotent stem cell lines recapitulate aspects of TDP-43 proteinopathies and reveal cell-specific vulnerability. *Proc. Natl. Acad. Sci. U. S. A.* **109**, 5803–5808 (2012) PMC3326463
94 Serio, A. *et al.* Astrocyte pathology and the absence of non-cell autonomy in an induced pluripotent stem cell model of TDP-43 proteinopathy. *Proc. Natl. Acad. Sci. U. S. A.* **110**, 4697–4702 (2013) PMC3607024
95 Marchetto, M. C. *et al.* A model for neural development and treatment of Rett syndrome using human induced pluripotent stem cells. *Cell* **143**, 527-539, doi:10.1016/j.cell.2010.10.016 (2010) PMC3003590

Answer ALS

Subject Number: _7_ 1_ _3__ - ___ ___ ___	Subject GUID: _N__ _E_ _U ___ ___ ___ ___ ___ ___ ___ ___
Date: ___ ___/ ___ ___/ _2_ _0_ _1_ _8_	Evaluator Initials: ___ ___ ___
Study Visit: **x** Screening Visit	

CREATE GUID
GUID ID Information Sheet – Collect at Screening Visit only AFTER the participant has signed the ICF. Information collected on this page will be utilized to generate the subject's GUID. Note that dates for date of birth should be entered as 1-31 for day, 1-12 for month, and 4 digits (YYYY) for year.

**Complete Legal Name at Birth (as it appears on birth certificate)**		
Legal, Given First **[REQUIRED]**	Legal, Given Middle **[REQUIRED]**	Legal, Given Last **[REQUIRED]**
Any Additional MIDDLE name(s) given at birth? **[OPTIONAL]**		
**DOB**		
Day of Birth[1-31] **[REQUIRED]**	Month of Birth[1-12] **[REQUIRED]**	Year of Birth[YYYY] **[REQUIRED]**

**Name of City/municipality at birth (as it appears on birth certificate) [REQUIRED]**	
**Country of Birth [REQUIRED]**	
**Physical Gender at birth [REQUIRED]**	○ Male<br>○ Female
**Gov't Issued or National ID [OPTIONAL]**	Not needed for this study
**Country issuing Gov't issued or National ID [OPTONAL]**	Not needed for this study

Please follow your institutional SOP regarding storing confidential patient data. Do not retain this form in the subject's binder.

Subject Number: _7_ 1_ _3__ - ___ ___ ___	Subject GUID: _N__ _E_ _U ___ ___ ___ ___ ___ ___ ___ ___
Date: ___ ___/ ___ ___/ _2_ _0_ _1_ _8_	Evaluator Initials: ___ ___ ___
Study Visit: **x** Screening Visit	

In addition to the subject ID, a patient Global Unique Identifier (GUID) will be used as the identifier for individuals participating in the study in NeuroBANK™. The GUID is an 11-character string that is generated using encryption technology and algorithms licensed by the NCRI from the National Institutes of Health (NIH).

The GUID is generated on a secure website that utilizes 128-bit Secure Socket Layer (SSL). Of note, this website is not linked to NeuroBANK™. The GUID is generated using an irreversible encryption algorithm – it accepts twelve identifying data elements, (e.g. last name at birth, first name at birth, gender at birth, day, month and year of birth, city and country of birth, etc.), and produces a unique random-generated character string, or GUID. No identifying information is stored in the system; it is simply used to generate the GUID. If the same information is entered again, the same GUID will be returned.

The GUID is entered into NeuroBANK™ when the patient is being created in the system. As the same patient may participate in multiple studies, NeuroBANK™ will also allow capturing a study-specific ID for the patient.

The Subject's 11-character GUID is

___ ___ ___ ___ ___ ___ ___ ___ ___ ___ ___

Answer ALS

Subject Number: _7_ 1_ _3__ - ___ ___ ___	Subject GUID: _N__ _E_ _U ___ ___ ___ ___ ___ ___ ___ ___
Date: ___ ___/ ___ ___/ _2_ _0_ _1_ _8_	Evaluator Initials: ___ ___ ___
Study Visit: **x** Screening Visit	

SCREENING VISIT CHECKLIST

Informed Consent:

- Was informed consent obtained from the subject?* ☒ Yes ☐ No
  (Written/verbal consent must be obtained **prior** to the start of any screening procedures)
- The consent form, version date __2/16/2017_____, for the Answer ALS study was thoroughly explained to the subject. The subject had adequate time to review the consent form and consider participation, and all questions regarding this study were answered. ☒ Yes ☐ No
- The subject was given a signed copy of the informed consent form. ☐ Yes ☐ No

Consenting process completed by Site Investigator or delegated study staff member per the delegation log of responsibilities: ☒ Yes ☐ No

Inclusion/Exclusion Review

☒ Inclusion/Exclusion Criteria Verification – *Complete Worksheet*

Screen Failure? ☐ Yes ☒ No (if yes complete worksheet)

Visit Procedures (* = Complete corresponding source worksheet and EDC)

☐ Collect Demographic Information*
☐ ALS History*
☐ ALS Gene Mutations*
☐ ALS Diagnosis / El Escorial Criteria*
☐ Medical History*
☐ Vital Signs*
☐ Measure Weight and Height*
☐ Key Study Event Review*
☐ Environmental/Social History*
☐ Risk Factor Assessment*
☐ Create GUID

Assessments & Outcomes

☐ ALSFRS-R*
☐ Ashworth Spasticity Scale*
☐ ALS Cognitive Behavioral Screen (ALS-CBS)*
☐ Slow Vital Capacity (SVC)*
☐ Hand Held Dynamometry*

Optional Procedures

☐ Lumbar Puncture for CSF Collection*
☐ Blood draw for Additional Blood samples (Uric Acid, Creatinine, Phosphorus, and Creatine Kinase (CK))*

Answer ALS

Subject Number: _7_ 1_ _3__ - ___ ___ ___	Subject GUID: _N__ _E_ _U ___ ___ ___ ___ ___ ___ ___ ___
Date: ___ ___/ ___ ___/ _2_ _0_ _1_ _8_	Evaluator Initials: ___ ___ ___
Study Visit: **x** Screening Visit	

Biomarker Studies

☐ Blood for PBMC* ☐ Plasma*

☐ Serum* ☐ DNA*

Protocol Deviation Review

Have there been any protocol deviations noted at this visit? ☐ Yes ☐ No

If **yes**, specify, and update the Protocol Deviation Log.

____

____

____

____

Concomitant Medication Review

Have there been any concomitant medications noted at this visit? ☐ Yes ☐ No

If **yes**, specify, and update the Con-Med Log.

____

____

____

Adverse Events (only Adverse Events that occur AFTER signing informed consent and are directly related to study procedures will be recorded)

Have there been any adverse events noted at this visit? ☐ Yes ☐ No

If **yes**, specify, and update the Adverse Event Log.

____

____

____

Visit Comments/Notes:

Signature of person obtaining information **Date**

Answer ALS

Subject Number: _7_ 1_ _3__ - ___ ___ ___	Subject GUID: _N__ _E_ _U ___ ___ ___ ___ ___ ___ ___ ___
Date: ___ ___/ ___ ___/ _2_ _0_ _1_ _8_	Evaluator Initials: ___ ___ ___
Study Visit: **x** Screening Visit	

INFORMED CONSENT FORM		
**Instructions:** Complete this form each time a subject signs a new version of the consent form.	**Yes**	**No**
1. Confirmed subject name, second identifier, and valid consent form.	☐	☐
2. Discussed, explained, and reviewed the consent form with the participant.	☐	☐
3. All of the participant’s questions were answered and/or concerns were addressed	☐	☐
4. The participant agreed to participate in the study and signed/dated a valid consent form prior to the start of any study procedures.	☐	☐
5. Caregiver/surrogate consent was obtained.	☐	☐
6. A copy of the signed and dated consent form was given to the participant.	☐	☐
7. A copy of the signed and dated consent form was placed in the subject’s binder.	☐	☐

Protocol Version: _______4___________

ICF Version/Date: ____2/16/2017_____

Answer ALS

Subject Number: _7_ 1_ _3__ - ___ ___ ___	Subject GUID: _N__ _E_ _U ___ ___ ___ ___ ___ ___ ___ ___
Date: ___ ___/ ___ ___/ _2_ _0_ _1_ _8_	Evaluator Initials: ___ ___ ___
Study Visit: **x** Screening Visit	

INCLUSION/EXCLUSION CRITERIA REVIEW			
**All subjects enrolled must meet eligibility criteria based on the inclusion/exclusion criteria detailed in the application and approved by the IRB.**			
**Inclusion Criteria**<br>**[NOTE: The answer to question 2, 3, or 4 must be "Yes" for the subject to be eligible.]**	**Yes**	**No**	**N/A**
1. The subject is male or female, aged 18 to 100, inclusive.	☐	☐	
2. The subject has been diagnosed with possible, laboratory-supported probable, probable or definite (according to the WFN El Escorial criteria) familial or sporadic ALS.	☐	☐	
3. The subject has a clinically diagnosed motor neuron disorder (MND), including primary lateral sclerosis (PLS), flail arm ALS, progressive muscular atrophy (PMA), monomelic amyotrophy, or another clinical variant of neurodegenerative MND (See Appendix 1 of Study Protocol).	☐	☐	
4. If the subject is an asymptomatic participant, he/she has documentation of the presence of a gene known to cause ALS.	☐	☐	☐
**Exclusion Criteria**<br>**[NOTE: If the answer to question 1 or 2 below is "Yes," then the patient is ineligible.]**	**Yes**	**No**	**N/A**
1. The participant has Spinal-Bulbar Muscular Atrophy.	☐	☐	
2. The participant has a known diagnosis of HIV/AIDS, Hepatitis B, or Hepatitis C.	☐	☐	

Subject Number: _7_ 1_ _3__ - ___ ___ ___	Subject GUID: _N__ _E_ _U ___ ___ ___ ___ ___ ___ ___ ___
Date: ___ ___/ ___ ___/ _2_ _0_ _1_ _8_	Evaluator Initials: ___ ___ ___
Study Visit: **x** Screening Visit	

**ELIGIBILITY CONFIRMATION**			
	**Yes**	**No**	**N/A**
Did the subject read, understand, and provide informed consent for this study?	☐	☐	
Date consent was received: ___ ___/___ ___/___ ___ ___ ___ (MM/DD/YYYY)<br>Person who Obtained Consent: ______________________________			
Consent Version:___4____<br>Is this a re-consent? ☐ Yes ☒ No			
Does the subject satisfy all inclusion and exclusion criteria for this study?	☐	☐	
Consent Process Description:	Explained study and procedures. Went through consent with participant. Explained risks and that they may withdraw at any time. Answered questions. Had participant sign consent.		

**STATEMENT OF ELIGIBILITY**	
This subject is [☐ **eligible** / ☐ **ineligible**] for participation in the study.	
Principal Investigator Signature:	Date:
Printed Name:	

Subject Number: _7_ 1_ _3__ - ___ ___ ___	Subject GUID: _N__ _E_ _U ___ ___ ___ ___ ___ ___ ___ ___
Date: ___ ___/ ___ ___/ _2_ _0_ _1_ _8_	Evaluator Initials: ___ ___ ___
Study Visit: **x** Screening Visit	

SCREEN FAILURE

Instructions: Please complete this form if the subject was considered ineligible and a protocol waiver(s) was not granted, if the subject decided to withdraw consent during the screening process, or for any other reason.

Please specify reason for screen failure:

☐ Subject did not meet inclusion criteria (Select all that apply):

☐ Failed inclusion criteria (list):

____

____

☐ Subject did not meet exclusion criteria (Select all that apply):

☐ Failed exclusion criteria (list):

____

____

☐ Subject withdrew consent during the screening process

☐ Other (specify): ____

____

____

____

____

____

____

Subject Number: _7_ 1_ _3__ - ___ ___ ___	Subject GUID: _N__ _E__ U___ ___ ___ ___ ___ ___ ___ ___
Date: ___ ___/ ___ ___/ _2_ _0_ _1_ _8_	Evaluator Initials: ___ ___ ___
Study Visit: **x** Screening Visit	

ALS History

Where was the ALS diagnosis made: ☐ Outside center ☐ ALS center

Date of symptom onset: ___ ___ / ___ ___ / ___ ___ ___ ___ (MM/DD/YYYY)

Date of diagnosis: ___ ___ / ___ ___ / ___ ___ ___ ___ (MM/DD/YYYY)

Site of onset – check all that apply

☐ Bulbar
- ☐ Speech ☐ Swallowing

☐ Axial
- ☐ Neck
- ☐ Trunk
- ☐ Respiratory

☐ Limb
- ☐ Upper
  - ☐ Left
  - ☐ Right
    - ☐ Hand/fingers ☐ Arm
- ☐ Lower
  - ☐ Left
  - ☐ Right
    - ☐ Ankle/foot/toes ☐ Leg

☐ Other, specify:

____

____

____

Answer ALS

Subject Number: _7_ 1_ _3__ - ___ ___ ___	Subject GUID: _N__ _E_ _U ___ ___ ___ ___ ___ ___ ___ ___
Date: ___ ___/ ___ ___/ _2_ _0_ _1_ _8_	Evaluator Initials: ___ ___ ___
Study Visit: **x** Screening Visit	

ALS Gene Mutation			
**Not Tested**	**Mutation**	**Result**	**Laboratory**
☐	ANG	☐ Positive<br>☐ Negative	
☐	C9ORF72	☐ Positive<br>☐ Negative	
☐	FUS	☐ Positive<br>☐ Negative	
☐	PROGRANULIN	☐ Positive<br>☐ Negative	
☐	SETX	☐ Positive<br>☐ Negative	
☐	SOD1	☐ Positive<br>☐ Negative	
☐	TAU	☐ Positive<br>☐ Negative	
☐	TDP-43	☐ Positive<br>☐ Negative	
☐	VAPB	☐ Positive<br>☐ Negative	
☐	VCP	☐ Positive<br>☐ Negative	
☐	Other, specify:	☐ Positive<br>☐ Negative	

Subject Number: _7_ 1_ _3__ - ___ ___ ___	Subject GUID: _N__ _E_ _U ___ ___ ___ ___ ___ ___ ___ ___
Date: ___ ___/ ___ ___/ _2_ _0_ _1_ _8_	Evaluator Initials: ___ ___ ___
Study Visit: x Screening Visit	

ALS DIAGNOSIS

Does the subject have:	Yes	No	Not Done
1. Topographical location and pattern of progression of UMN and LMN signs, including signs of spread within a region or to other regions, consistent with ALS?	☐	☐	
2. Exclusion by electrophysiological testing of all other processes including conduction block that might explain the underlying signs and symptoms?	☐	☐	☐
3. Exclusion by neuroimaging of other disease processes such as myelopathy or radiculopathy that might explain observed clinical and electrophysiological signs?	☐	☐	☐

Please check YES or NO if signs are present.

	CLINICAL						EMG		
	UMN			LMN			LMN		
	Yes	No	Not Done	Yes	No	Not Done	Yes	No	Not Done
BULBAR	☐	☐	☐	☐	☐	☐	☐	☐	☐
LUE	☐	☐	☐	☐	☐	☐	☐	☐	☐
RUE	☐	☐	☐	☐	☐	☐	☐	☐	☐
TRUNK	☐	☐	☐	☐	☐	☐	☐	☐	☐
LLE	☐	☐	☐	☐	☐	☐	☐	☐	☐
RLE	☐	☐	☐	☐	☐	☐	☐	☐	☐

Subject Number: _7_ 1_ _3__ - ___ ___ ___	Subject GUID: _N__ _E_ _U ___ ___ ___ ___ ___ ___ ___ ___
Date: ___ ___/ ___ ___/ _2_ _0_ _1_ _8_	Evaluator Initials: ___ ___ ___
Study Visit: **x** Screening Visit	

ALS DIAGNOSIS

El Escorial criteria for ALS (select one):

☐ Suspected

☐ Possible

☐ Probable laboratory supported

☐ Probable

☐ Definite

______________________________ ______________________________

Site Investigator Signature **Date** (MM/DD/YYYY)

Answer ALS

Subject Number: _7_ 1_ _3__ - ___ ___ ___	Subject GUID: _N__ _E_ _U ___ ___ ___ ___ ___ ___ ___ ___
Date: ___ ___/ ___ ___/ _2_ _0_ _1_ _8_	Evaluator Initials: ___ ___ ___
Study Visit: **x** Screening Visit	

Family History

Instructions: Enter one relative per line. Make as many copies of this form as necessary to record all family history.

**Relative** (enter code or specify)	**Hereditary**	**Gender**	**Medical Condition** (enter code(s))	**Genetic Testing Performed?**	**Known Mutation** (enter code)
	☐ Paternal<br>☐ Maternal	☐ Male<br>☐ Female		☐ Yes<br>☐ No	
	☐ Paternal<br>☐ Maternal	☐ Male<br>☐ Female		☐ Yes<br>☐ No	
	☐ Paternal<br>☐ Maternal	☐ Male<br>☐ Female		☐ Yes<br>☐ No	
	☐ Paternal<br>☐ Maternal	☐ Male<br>☐ Female		☐ Yes<br>☐ No	
	☐ Paternal<br>☐ Maternal	☐ Male<br>☐ Female		☐ Yes<br>☐ No	
	☐ Paternal<br>☐ Maternal	☐ Male<br>☐ Female		☐ Yes<br>☐ No	
	☐ Paternal<br>☐ Maternal	☐ Male<br>☐ Female		☐ Yes<br>☐ No	

**Relative:**	**Medical Condition – select all that apply:**	**Known Mutations:**
1=Mother<br>2=Father<br>3=Sister<br>4=Brother<br>5=Half-sister<br>6=Half Brother<br>7=Daughter<br>8=Son<br>9=Grandmother<br>10=Grandfather<br>11=Aunt<br>12=Uncle<br>13=Cousin<br>14=OTHER, Specify:	1=Alzheimer's disease<br>2=ALS<br>3=Dementia<br>4=Down's syndrome<br>5=Frontotemporal Dementia<br>6=Huntington's disease<br>7=Parkinson's disease<br>8=Psychiatric disorder, specify:<br>9=Arthritis<br>10=Asthma<br>11=Cancer<br>12=Circulation problems<br>13=Diabetes<br>14=Heart Disease<br>15=Lung disease<br>16=Stroke<br>17=OTHER, Specify:	1=ANG<br>2=C9ORF72<br>3=FUS<br>4=SETX<br>5=SOD1<br>6=TDP-43<br>7=VAPB<br>8=VCP<br>9= OTHER, Specify:

Answer ALS

Subject Number: _7_ 1_ _3__ - ___ ___ ___	Subject GUID: _N__ _E_ _U ___ ___ ___ ___ ___ ___ ___ ___
Date: ___ ___/ ___ ___/ _2_ _0_ _1_ _8_	Evaluator Initials: ___ ___ ___
Study Visit: **x** Screening Visit	

MEDICAL HISTORY		
**Instructions:** Please enter any and all medical history the patient may have experienced in their lifetime. Make as many copies of this form as necessary to record all relevant past medical history.		
**Description**	**Year of Diagnosis (YYYY)**	**Still Present? (Yes/No)**
		☐ Yes ☐ No
		☐ Yes ☐ No
		☐ Yes ☐ No
		☐ Yes ☐ No
		☐ Yes ☐ No
		☐ Yes ☐ No
		☐ Yes ☐ No
		☐ Yes ☐ No
		☐ Yes ☐ No
		☐ Yes ☐ No
		☐ Yes ☐ No
		☐ Yes ☐ No
		☐ Yes ☐ No
		☐ Yes ☐ No

Subject Number: _7_ 1_ _3__ - ___ ___ ___	Subject GUID: _N__ _E_ __U ___ ___ ___ ___ ___ ___ ___ ___
Date: ___ ___/ ___ ___/ _2_ _0_ _1_ _8_	Evaluator Initials: ___ ___ ___
Study Visit: **x** Screening Visit	

**DEMOGRAPHICS**
Date of Birth: ___ ___ / ___ ___ / ___ ___ ___ ___ (MM/DD/YYYY)
Age: ___________
Gender: ☐ Male ☐ Female
*Ethnic Category (Select **one**):<br>☐ Non-Hispanic or Latino<br>☐ Hispanic or Latino
*Racial Categories (Select **all that apply**):<br>☐ White<br>☐ Black/African American<br>☐ Asian<br>☐ Native Hawaiian/Pacific Islander<br>☐ American Indian/Alaska Native
***PLEASE NOTE: Ethnic and Racial categories collected via subject self-report**

Answer ALS

Subject Number: _7_ 1_ _3__ - ___ ___ ___	Subject GUID: _N__ _E__U__ __ __ __ __ __ __ __
Date: ___ ___/ ___ ___/ _2_ _0_ _1_ _8_	Evaluator Initials: ___ ___ ___
Study Visit: **x** Screening Visit	

CNS-LABILITY SCALE

Date Performed: _____ _____ / _____ _____ / _____ _____ _____ _____

Evaluator's Initials: ________________

INSTRUCTIONS FOR SUBJECT

Please select the number that describes the degree to which each item has applied to you DURING THE PAST WEEK.

	Does not Apply	Rarely Applies	Occasionally Applies	Frequently Applies	Applies Most of the Time
1. There are times when I feel fine 1 minute, and then I'll become tearful the next over something small or for no reason at all.	1	2	3	4	5
2. Others have told me that I seem to become amused very easily or that I seem to become amused about things that aren't funny.	1	2	3	4	5
3. I find myself crying very easily.	1	2	3	4	5
4. I find that even when I try to control my laughter, I am often unable to do so.	1	2	3	4	5
5. There are times when I won't be thinking of anything happy or funny at all, but then I'll suddenly be overcome by funny or happy thoughts.	1	2	3	4	5
6. I find that even when I try to control my crying, I am often unable to do so.	1	2	3	4	5
7. I find that I am easily overcome by laughter.	1	2	3	4	5

Answer ALS

Subject Number: _7_ 1_ _3__ - ___ ___ ___	Subject GUID: _N__ _E_ _U ___ ___ ___ ___ ___ ___ ___ ___
Date: ___ ___/ ___ ___/ _2_ _0_ _1_ _8_	Evaluator Initials: ___ ___ ___
Study Visit: **x** Screening Visit	

VITAL SIGNS				
**Not Done**	**Test**	**Measurement**	**Unit**	**Measurement Specification**
☐	Temperature	______.____	☐ °F<br>☒ °C	Method (Select One):<br>☐ Axillary<br>☐ Oral<br>☐ Rectal<br>☐ Tympanic<br>☒ Other (specify):__temporal______
☐	Blood Pressure	Systolic: ______<br>Diastolic: ______	mmHg	Position (Select One):<br>☐ Standing ☐ Left Arm<br>☒ Sitting ☐ Right Arm<br>☐ Supine
☐	Heart Rate	______	beats/min	
☐	Respiratory Rate	______	breaths/min	
☐	Weight	______	☒ pounds<br>☐ kilograms	
☐	Height *(Screening Only)*	______	☒ inches<br>☐ centimeters	
☐	BMI	______		

Subject Number: _7_ 1_ _3__ - ___ ___ ___	Subject GUID: _N__ _E_ _U ___ ___ ___ ___ ___ ___ ___ ___
Date: ___ ___/ ___ ___/ _2_ _0_ _1_ _8_	Evaluator Initials: ___ ___ ___
Study Visit: **x** Screening Visit	

VITAL CAPACITY

Instructions: A printout from the spirometer of all Slow Vital Capacity (SVC) or Forced Vital Capacity (FVC) trials will be retained. Three VC trials are required for each testing session, however up to 5 trials may be performed if the variability between the highest and second highest VC is 10% or greater for the first 3 trials. Up to three of the best trials are recorded on the CRF in the EDC.

Type of Vital Capacity Collected:

☐ SVC
☐ FVC

Position:

☐ Supine
☐ Upright
☐ Unknown

PLACE SPIROMETER PRINTOUT HERE

****Must be signed and dated by the Evaluator****

Answer ALS

Subject Number: _7_ 1_ _3__ - ___ ___ ___	Subject GUID: _N__ _E_ _U ___ ___ ___ ___ ___ ___ ___ ___
Date: ___ ___/ ___ ___/ _2_ _0_ _1_ _8_	Evaluator Initials: ___ ___ ___
Study Visit: **x** Screening Visit	

**ALSFRS-R**	
Was the ALSFRS-R Performed? ☐ Yes ☐ No	
Responded by: ☐ Patient ☐ Patient via Caregiver	
Mode of Administration: ☐ In person ☐ Telephone ☐ Other:______________________	
**QUESTIONS:**	SCORE
**1. Speech**<br>4 = Normal speech processes<br>3 = Detectable speech disturbances<br>2 = Intelligible with repeating<br>1 = Speech combined with non-vocal communication<br>0 = Loss of useful speech	
**2. Salivation**<br>4 = Normal<br>3 = Slight but definite excess of saliva in mouth; may have nighttime drooling<br>2 = Moderately excessive saliva; may have minimal drooling<br>1 = Marked excess of saliva with some drooling<br>0 = Marked drooling; requires constant tissue or handkerchief	
**3. Swallowing**<br>4 = Normal eating habits<br>3 = Early eating problems – occasional choking<br>2 = Dietary consistency changes<br>1 = Needs supplemental tube feeding<br>0 = NPO (exclusively parenteral or enteral feeding)	
**4. Handwriting**<br>4 = Normal<br>3 = Slow or sloppy; all words are legible<br>2 = Not all words are legible<br>1 = No words are legible but can still grip a pen<br>0 = Unable to grip pen	
**5a. Cutting Food and Handling Utensils (patients without gastrostomy)**<br>4 = Normal<br>3 = Somewhat slow and clumsy, but no help needed<br>2 = Can cut most foods, although clumsy and slow; some help needed<br>1 = Food must be cut by someone, but can still feed slowly<br>0 = Needs to be fed	
**5b. Cutting Food and Handling Utensils (alternate scale for patients with gastrostomy)**<br>4 = Normal<br>3 = Clumsy, but able to perform all manipulations independently<br>2 = Some help needed with closures and fasteners<br>1 = Provides minimal assistance to caregivers<br>0 = Unable to perform any aspect of task	

Subject Number: _7_ 1_ _3__ - ___ ___ ___	Subject GUID: _N__ _E__ _U__ ___ ___ ___ ___ ___ ___ ___ ___
Date: ___ ___/ ___ ___/ _2_ _0_ _1_ _8_	Evaluator Initials: ___ ___ ___
Study Visit: **x** Screening Visit	

**6. Dressing and Hygiene**<br>4 = Normal function<br>3 = Independent, can complete self-care with effort or decreased efficiency<br>2 = Intermittent assistance or substitute methods<br>1 = Needs attendant for self-care<br>0 = Total dependence	
**7. Turning in Bed and Adjusting Bed Clothes**<br>4 = Normal function<br>3 = Somewhat slow and clumsy, but no help needed<br>2 = Can turn alone, or adjust sheets, but with great difficulty<br>1 = Can initiate, but not turn or adjust sheets alone<br>0 = Helpless	
**8. Walking**<br>4 = Normal<br>3 = Early ambulation difficulties<br>2 = Walks with assistance<br>1 = Nonambulatory functional movement only<br>0 = No purposeful leg movement	
**9. Climbing Stairs**<br>4 = Normal<br>3 = Slow<br>2 = Mild unsteadiness or fatigue<br>1 = Needs assistance<br>0 = Cannot do	
**R-1. Dyspnea**<br>4 = None<br>3 = Occurs when walking<br>2 = Occurs with one or more of the following: eating, bathing, dressing<br>1 = Occurs at rest, difficulty breathing when either sitting or lying<br>0 = Significant difficulty, considering using mechanical respiratory support	
**R-2 Orthopnea**<br>4 = None<br>3 = Some difficulty sleeping at night due to shortness of breath, does not routinely use more than two pillows<br>2 = Needs extra pillow in order to sleep (more than two)<br>1 = Can only sleep sitting up<br>0 = Unable to sleep without mechanical assistance	
**R-3 Respiratory Insufficiency**<br>4 = None<br>3 = Intermittent use of NIPPV<br>2 = Continuous use of NIPPV during the night<br>1 = Continuous use of NIPPV during the night and day<br>0 = Invasive mechanical ventilation by intubation or tracheostomy	

Answer ALS

Subject Number: _7_ 1_ _3__ - ___ ___ ___	Subject GUID: _N__ _E__ _U ___ ___ ___ ___ ___ ___ ___ ___
Date: ___ ___/ ___ ___/ _2_ _0_ _1_ _8_	Evaluator Initials: ___ ___ ___
Study Visit: **x** Screening Visit	

Hand page 2 to caregiver **ALS Cognitive Behavioral Screen (CBS)™**

Attention

a. Commands: *I am going to say some commands. Please listen carefully and then do what I say. (If patient is unable to indicate with finger, movement can be substituted with eyes, arm or other means).*

1. Point/indicate (with your finger) to the ceiling and then make a fist.
2. Touch your shoulder, point to the floor, and then make a fist.

#errors	0	1+
Score	1	0

b. Mental Addition/Language: I am going to say some phrases. I want you to tell me the number of syllables in each phrase. For example, "the table" has 3 syllables. (Repetition of each phrase is allowed once)

1. The weather is nice (correct response: 5) answer ___
2. Tomorrow will be sunny (correct response: 7) answer ___

#errors	0	1+
Score	1	0

(score 0 if >20 sec on either)

c. Eye movements: Saccades and Antisaccades

\# of correct saccades out of 8: ____/8 Score: 8/8 = 1 point, ≤7/8 = 0 points

\# of correct antisaccades out of 8: ____/8 Score: 8/8 = 2 points, ≤7/8 = 1 point, ≤6/8=0 points

/5

Concentration

I am going to say some numbers. After I say them, I want you to say them to me backwards, or in reverse order. For example, if I say 3-6, you would say 6-3. (If written, do not allow pt to write forward span. Discontinue after failure on 2 consecutive trials).

	Correct	Incorrect		Correct	Incorrect
2-9 (9-2)	_____	____	7-8-6-4 (4-6-8-7)	____	____
6-4 (4-6)	_____	____	5-4-1-9 (9-1-4-5)	____	____
3-7-2 (2-7-3)	_____	_____	8-2-5-9-3 (3-9-5-2-8)	____	_____
5-8-1 (1-8-5)	_____	_____	5-7-6-3-9 (9-3-6-7-5)	____	_____

Maximum Span Correct:

/5

Tracking/Monitoring

a. Months: *Please say the months of the year backwards, starting with Dec.*

Dec Nov Oct Sept Aug Jul June May Apr Mar Feb Jan

#errors	0	1	2+
Score	2	1	0

b. Alphabet: *Please say/write the alphabet*

A B C D E F G H I J K L M N O P Q R S T U V W X Y Z

#errors	0	1+
Score	1	0

c. Alternation Task*: I want you to alternate between numbers and letters, starting with 1-A, 2-B, 3-C, and so on. Please continue from there, alternating between # and letter, in order, until I tell you to stop.*
(Errors: any mistake in sequencing, i.e. 7-H or 8-9)

4-D 5-E 6-F 7-G 8-H 9-I 10-J 11-K 12-L 13-M

#errors	0	1	2
Score	2	1	0

/5

Initiation and Retrieval

Say/write as many words as you can think of starting with the letter F, as quickly as you can, in 1 min. You can't say/write the names of people, places or numbers. Please don't say/write the same word with a different ending, like truck and trucks. (S words can be substituted for F words). Errors: repetitions, rule violations.

1. ____________	9. ____________	17. ____________
2. ____________	10. ____________	18. ____________
3. ____________	11. ____________	19. ____________
4. ____________	12. ____________	20. ____________
5. ____________	13. ____________	
6. ____________	14. ____________	
7. ____________	15. ____________	
8. ____________	16. ____________	

#correct words	>12	12-8	<8	≤4
Score:	3	2	1	0*

plus

#errors	0	1	2+
Score	2	1	0

*if ≤4 words, total verbal fluency score = 0 regardless of # of errors

/5

Copyright© 2006-2011 Sutter West Bay Hospitals, aba California Pacific Medical Center. All rights reserved.

TOTAL SCORE **/20**

Answer ALS

Subject Number: _7_ 1_ _3__ - ___ ___ ___	Subject GUID: _N__ _E_ _U ___ ___ ___ ___ ___ ___ ___ ___
Date: ___ ___/ ___ ___/ _2_ _0_ _1_ _8_	Evaluator Initials: ___ ___ ___
Study Visit: **x** Screening Visit	

Answer ALS

Subject Number: _7_ 1_ _3__ - __ __ __	Subject GUID: _N__ _E__ _U__ __ __ __ __ __ __ __ __
Date: __ __/ __ __/ _2_ _0_ _1_ _8_	Evaluator Initials: __ __ __
Study Visit: **x** Screening Visit	

Caregiver Initials: __ __ __ relationship:__________

ALS CBS™ ALS Cognitive Behavioral Screen Caregiver Questionnaire

These questions pertain to possible changes that you have noticed since the onset of ALS symptoms. As best you can, consider changes that are unrelated to physical weakness. For example, question #1 asks about interest in activities. If the person can no longer play tennis but still seems interested in it (i.e. talks about it, watches it on t.v.), then you would circle 3 for no change in level of interest.

If the person has always had the trait in question, please respond No Change, since there has been no change over time.

**Compared to before ALS, does he/she:** *Please circle a number*	**No Change**	**Small Change**	**Medium Change**	**Large Change**
1. Have less interest in topics/events that used to be important to them?	3	2	1	0
2. Show little emotion, or seem less responsive emotionally?	3	2	1	0
3. Seem more agreeable or pleasant than in the past with fewer worries?	3	2	1	0
4. Fail to think things through before acting?	3	2	1	0
5. Seem more withdrawn from others but not sad?	3	2	1	0
6. Get confused or distracted more easily?	3	2	1	0
7. Have less ability to deal with frustration or stress?	3	2	1	0
8. Seem less concerned about the feelings or concerns of others than before:	3	2	1	0
9. Get angry or irritable more easily than before?	3	2	1	0
10. Seem more sarcastic or childlike than before?	3	2	1	0
11. Eat more or have a new preference for particular foods (i.e. sweets)?	3	2	1	0
12. Have more trouble changing opinions or adapting to new situations?	3	2	1	0
13. Show less judgment or more problems making good decisions (i.e. regarding safety, finances, etc)	3	2	1	0
14. Have less awareness of obvious problems or changes, or deny them?	3	2	1	0
15. Have new problems with language, such as saying the wrong word more often, making up new words, or declines in spelling ability?	3	2	1	0

TOTAL SCORE: ____ / 45

The following questions relate to current symptoms, not changes over time:

Do you think your loved one:	**YES**	**NO**
• Seems depressed on most days?	[ ]	[ ]
• Seems anxious on most days?	[ ]	[ ]
• Seems extremely fatigued on most days?	[ ]	[ ]
• Suffers from unexpected crying or laughing spells?	[ ]	[ ]

Copyright© 2006-2011 Sutter West Bay Hospitals, aba California Pacific Medical Center. All rights reserved.

Answer ALS

Subject Number: _7_ 1_ _3__ - ___ ___ ___	Subject GUID: _N__ _E_ __U ___ ___ ___ ___ ___ ___ ___ ___
Date: ___ ___/ ___ ___/ _2_ _0_ _1_ _8_	Evaluator Initials: ___ ___ ___
Study Visit: **x** Screening Visit	

Subject Number: _7_ 1_ _3__ - ___ ___ ___	Subject GUID: _N__ _E_ _U ___ ___ ___ ___ ___ ___ ___ ___
Date: ___ ___/ ___ ___/ _2_ _0_ _1_ _8_	Evaluator Initials: ___ ___ ___
Study Visit: **x** Screening Visit	

ASHWORTH SPASTICITY SCALE
Date Performed: _____ _____ / _____ _____ / _____ _____ _____ _____
Evaluator's Initials: __________________
*Key:<br>1 No increase in muscle tone<br>2 Slight increase in tone giving a "catch" when affected part is moved in flexion or extension<br>3 More marked increase in tone but affected part is easily flexed.<br>4 Considerable increase in tone; passive movement difficult.<br>5 Affected part is rigid in flexion or extension.<br>6 Not Tested<br>7 Not Tested (subject unable to perform task)

**Not Done**	**Limb**	**Score***
☐	Right Arm	
☐	Left Arm	
☐	Right Leg	
☐	Left Leg	

Answer ALS

Subject Number: _7_ 1_ _3__ - ___ ___ ___	Subject GUID: _N__ _E_ _U ___ ___ ___ ___ ___ ___ ___ ___
Date: ___ ___/ ___ ___/ _2_ _0_ _1_ _8_	Evaluator Initials: ___ ___ ___
Study Visit: **x** Screening Visit	

REFLEXES
Date Performed: _____ _____ / _____ _____ / _____ _____ _____ _____
Evaluator's Initials: _________________

Instructions:

If the reflex was a **1+ or 2+,** was the reflex ABNORMALLY retained in a weak/wasted limb suggesting hyperreflexia? Please answer by checking the appropriate box as indicated below. 0 = Absent 1+ = Present

Not Done	Cranial	Absent	Present
❑	Jaw Jerk	❍	❍
❑	Facial Reflex	❍	❍
❑	Palmomental Sign	❍	❍

Not Done	Right Cervical	0 (Absent)	1+ (Present)	2+	3+	4+	Retained in Weak Limb?
❑	Triceps Reflex	❍	❍	❍	❍	❍	❍Yes ❍No ❍N/A
❑	Biceps Reflex	❍	❍	❍	❍	❍	❍Yes ❍No ❍N/A
❑	Brachioradialis	❍	❍	❍	❍	❍	❍Yes ❍No ❍N/A
❑	Finger Flexors	❍	❍				
❑	Clonus	❍	❍				
❑	Hoffman's Sign	❍	❍				

Not Done	Left Cervical	0 (Absent)	1+ (Present)	2+	3+	4+	Retained in Weak Limb?
❑	Triceps Reflex	❍	❍	❍	❍	❍	❍Yes ❍No ❍N/A
❑	Biceps Reflex	❍	❍	❍	❍	❍	❍Yes ❍No ❍N/A
❑	Brachioradialis	❍	❍	❍	❍	❍	❍Yes ❍No ❍N/A
❑	Finger Flexors	❍	❍				
❑	Clonus	❍	❍				
❑	Hoffman's Sign	❍	❍				

Answer ALS

Subject Number: _7_ 1_ _3__ - ___ ___ ___	Subject GUID: _N__ _E_ _U ___ ___ ___ ___ ___ ___ ___ ___
Date: ___ ___/ ___ ___/ _2_ _0_ _1_ _8_	Evaluator Initials: ___ ___ ___
Study Visit: **x** Screening Visit	

Not Done	Right Lumbosacral	0 (Absent)	1+ (Present)	2+	3+	4+	Retained in Weak Limb?
❑	Patellar	❍	❍	❍	❍	❍	❍Yes ❍No ❍N/A
❑	Ankle	❍	❍	❍	❍	❍	❍Yes ❍No ❍N/A
❑	Crossed Adduction	❍	❍				
❑	Clonus	❍	❍				
❑	Babinski Sign	❍	❍				

Not Done	Left Lumbosacral	0 (Absent)	1+ (Present)	2+	3+	4+	Retained in Weak Limb?
❑	Patellar	❍	❍	❍	❍	❍	❍Yes ❍No ❍N/A
❑	Ankle	❍	❍	❍	❍	❍	❍Yes ❍No ❍N/A
❑	Crossed Adduction	❍	❍				
❑	Clonus	❍	❍				
❑	Babinski Sign	❍	❍				

Answer ALS

Subject Number: _7_ 1_ _3__ - ___ ___ ___	Subject GUID: _N__ _E_ _U ___ ___ ___ ___ ___ ___ ___ ___
Date: ___ ___/ ___ ___/ _2_ _0_ _1_ _8_	Evaluator Initials: ___ ___ ___
Study Visit: **x** Screening Visit	

HAND HELD DYNAMOMETRY (HHD)

Date Performed: __ __ /__ __/ __ __ __ __

Evaluator's Initials: ___________

Not Tested	Muscle	Check if Not Done	Trial 1 (lbs)	Trial 2 (lbs)	Trial 3 (lbs) (if needed)	Able to Break		Not Done Reason
						Y	N	
	LEFT SHOULDER FLEXION	❑				❑	❑	❑ Too weak<br>❑ Other:
	RIGHT SHOULDER FLEXION	❑				❑	❑	❑ Too weak<br>❑ Other:
	LEFT ELBOW FLEXION	❑				❑	❑	❑ Too weak<br>❑ Other:
	RIGHT ELBOW FLEXION	❑				❑	❑	❑ Too weak<br>❑ Other:
	LEFT ELBOW EXTENSION	❑				❑	❑	❑ Too weak<br>❑ Other:
	RIGHT ELBOW EXTENSION	❑				❑	❑	❑ Too weak<br>❑ Other:
	LEFT WRIST EXTENSION	❑				❑	❑	❑ Too weak<br>❑ Other:
	RIGHT WRIST EXTENSION	❑				❑	❑	❑ Too weak<br>❑ Other:
	LEFT HIP FLEXION	❑				❑	❑	❑ Too weak<br>❑ Other:
	RIGHT HIP FLEXION	❑				❑	❑	❑ Too weak<br>❑ Other:
	LEFT KNEE FLEXION	❑				❑	❑	❑ Too weak<br>❑ Other:
	RIGHT KNEE FLEXION	❑				❑	❑	❑ Too weak<br>❑ Other:
	LEFT KNEE EXTENSION	❑				❑	❑	❑ Too weak<br>❑ Other:
	RIGHT KNEE EXTENSION	❑				❑	❑	❑ Too weak<br>❑ Other:
	LEFT ANKLE DORSIFLEXION	❑				❑	❑	❑ Too weak<br>❑ Other:
	RIGHT ANKLE DORSIFLEXION	❑				❑	❑	❑ Too weak<br>❑ Other:
	LEFT FIRST DORSAL INTEROSSEOUS	❑				❑	❑	❑ Too weak<br>❑ Other:
	RIGHT FIRST DORSAL INTEROSSEOUS	❑				❑	❑	❑ Too weak<br>❑ Other:

Answer ALS

Subject Number: _7_ 1_ _3__ - ___ ___ ___	Subject GUID: _N__ _E_ _U ___ ___ ___ ___ ___ ___ ___ ___
Date: ___ ___/ ___ ___/ _2_ _0_ _1_ _8_	Evaluator Initials: ___ ___ ___
Study Visit: **x** Screening Visit	

GRIP STRENGTH TESTING

Date Performed: __ __ / __ __ / __ __

Evaluator's Initials:

Not Tested		Trial 1 (pounds)	Trial 2 (pounds)	If "Not Tested", explain
	**LEFT GRIP**<br>**Setting:**			○ **Too weak**<br>○ **Other:**
	**RIGHT GRIP**<br>**Setting:**			○ **Too weak**<br>○ **Other:**

Brief Environmental Questionnaire

Geography:

In what city/state do you live? ________________

In the time prior to your diagnosis, in what city/state did you live? ________________

Toxin Exposure:

Have you used any of the following products more than twice per month for at least 6 months (check all that apply)?

- ☐ Insecticide sprays inside your home
- ☐ Insecticide sprays outside your home
- ☐ Herbicides

Head injury (more than one year prior to symptom onset):

Have you ever been admitted to the hospital for a head injury? ○ Yes ○ No

Have you ever been seen in the ED for a head injury? ○ Yes ○ No

Have you had any concussions? ○ Yes ○ No

If so, how many? ________________

Habits:

Have you ever been a smoker? ○ Yes ○ No

Are you an active smoker? ○ Yes ○ No

If so, for how many years? ________________

How many packs per day (on average)? ________________

How much alcohol do you drink per week, if any? ________________ (drinks per week)

In the 10 years prior to your diagnosis, approximately how much alcohol did you drink (on average per week), if any? ________________ (drinks per week)

Prior to your symptom onset:

Subject Number: _7_ 1_ _3__ - ___ ___ ___	Subject GUID: _N__ _E_ _U ___ ___ ___ ___ ___ ___ ___ ___
Date: ___ ___/ ___ ___/ _2_ _0_ _1_ _8_	Evaluator Initials: ___ ___ ___
Study Visit: **x** Screening Visit	

How many marathons have you run? ________________
How many days per week do you exercise at least moderately (break a sweat)? ________________

Military:
Were you in the military? ○ Yes ○ No
How many years? ________________
Were you deployed outside the US? ________________
If so, what years? ________________
To where? ________________

Work History:
Which of the following occupations have you had for at least 1 year (check all that apply)?
- ❑ Management Occupations
- ❑ Business and Financial Operations Occupations
- ❑ Computer and Mathematical Occupations
- ❑ Architecture and Engineering Occupations
- ❑ Life, Physical, and Social Science Occupations
- ❑ Community and Social Service Occupations
- ❑ Legal Occupations
- ❑ Education, Training, and Library Occupations
- ❑ Arts, Design, Entertainment, Sports, and Media Occupations
- ❑ Healthcare Practitioners and Technical Occupations
- ❑ Healthcare Support Occupations
- ❑ Protective Service Occupations
- ❑ Food Preparation and Serving Related Occupations
- ❑ Building and Grounds Cleaning and Maintenance Occupations
- ❑ Personal Care and Service Occupations
- ❑ Sales and Related Occupations
- ❑ Office and Administrative Support Occupations
- ❑ Farming, Fishing, and Forestry Occupations
- ❑ Construction and Extraction Occupations
- ❑ Installation, Maintenance, and Repair Occupations
- ❑ Production Occupations
- ❑ Transportation and Material Moving Occupations
- ❑ Military Specific Occupations

Sports History:

Subject Number: _7_ 1_ _3__ - ___ ___ ___	Subject GUID: _N__ _E_ _U ___ ___ ___ ___ ___ ___ ___ ___
Date: ___ ___/ ___ ___/ _2_ _0_ _1_ _8_	Evaluator Initials: ___ ___ ___
Study Visit: **x** Screening Visit	

Sport (Circle One)	Level (High School, College, Amateur, Recreational, Professional)	Number of Years
Soccer<br>Football<br>Baseball<br>Hockey (ice/field)<br>Lacrosse<br>Track & Field/Distance<br>Running<br>Swimming<br>Tennis<br>Golf<br>Other:		

PBMC COLLECTION (Cedars)

Instructions: Sites are to follow the Cedars SOP titled "Peripheral Blood Collection and Processing for Reprogramming to iPSCs" or "Peripheral Blood Collection and Processing for Cryopreservation."

Please refer to section H of the site MOP for complete collection, processing, and shipping details.

Date Collected: _____ _____ / _____ _____ / _____ _____ _____ _____

Collector's Initials: __________________

Number of Tubes Collected: __________________

Date Tubes Shipped to Cedars-Sinai: _____ _____ / _____ _____ / _____ _____ _____ _____

DNA – WHOLE BLOOD COLLECTION (NYGC)

Answer ALS

Subject Number: _7_ 1_ _3__ - ___ ___ ___	Subject GUID: _N__ _E__U ___ ___ ___ ___ ___ ___ ___ ___
Date: ___ ___/ ___ ___/ _2_ _0_ _1_ _8_	Evaluator Initials: ___ ___ ___
Study Visit: **x** Screening Visit	

**Instructions:** All samples will be collected in accordance with the policies and guidelines of the site's institution.<br>Please refer to section H of the site MOP for complete collection, processing, and shipping details.
Date Collected: _____ _____ / _____ _____ / _____ _____ _____ _____
Collector's Initials: _________________
Time Collected: ____ ____ : ____ ____ (24-Hr Clock)
Number of tubes collected: _________________
Date Tubes Shipped to the NYGC: _____ _____ / _____ _____ / _____ _____ _____ _____

**DNA – ALIQUOT COLLECTION (MGH Biorepository)**<br>**Instructions:** All samples will be collected in accordance with the policies and guidelines of the site's institution.<br>Please refer to section H of the site MOP for complete collection, processing, and shipping details.
Date Collected: _____ _____ / _____ _____ / _____ _____ _____ _____
Collector's Initials: _________________
Collection Number:
Time Collected: ____ ____ : ____ ____ (24-Hr Clock)
Time of aliquot: ____ ____ : ____ ____ (24-Hr Clock)
Number of 1.0mL aliquots:
Volume of LAST aliquot if less than 1.0mL:
Time aliquots put on dry ice: ____ ____ : ____ ____ (24-Hr Clock)
Time aliquots put in -70ºC or -80ºC freezer: ____ ____ : ____ ____ (24-Hr Clock)

Subject Number: _7_ 1_ _3__ - ___ ___ ___	Subject GUID: _N__ _E_ _U ___ ___ ___ ___ ___ ___ ___ ___
Date: ___ ___/ ___ ___/ _2_ _0_ _1_ _8_	Evaluator Initials: ___ ___ ___
Study Visit: **x** Screening Visit	

**PLASMA COLLECTION (MGH Biorepository)**<br>**Instructions:** Blood samples will be collected in accordance with the policies and guidelines of the site's institution.<br><br>Please refer to section H of the site MOP for complete collection, processing, and shipping details.	
Date Plasma Samples Collected: _____ _____ / _____ _____ / _____ _____ _____ _____	
Collector's Initials: _________________	
________<br>Collection Number:	Place Sample Label Here
Time Collected:	____ ____ : ____ ____ (24-Hr Clock)
Time centrifugation started:	____ ____ : ____ ____ (24-Hr Clock)
Speed of centrifugation:	__1750__ x gravity (g)
Duration of centrifugation:	___10____ minutes
Time aliquoted:	____ ____ : ____ ____ (24-Hr Clock)
Time aliquots put on dry ice:	____ ____ : ____ ____ (24-Hr Clock)
Time aliquots put in -70ºC or -80ºC freezer:	____ ____ : ____ ____ (24-Hr Clock)
Did plasma remain pink after centrifugation, indicating hemolysis?	❍ Yes ❍ No

Number of full (0.5mL) aliquots: ______________

Volume of aliquots: **0.5mL**

Volume of LAST aliquot if less than 0.5mL: _______________

Subject Number: _7_ 1_ _3__ - ___ ___ ___	Subject GUID: _N__ _E_ _U ___ ___ ___ ___ ___ ___ ___ ___
Date: ___ ___/ ___ ___/ _2_ _0_ _1_ _8_	Evaluator Initials: ___ ___ ___
Study Visit: **x** Screening Visit	

**SERUM COLLECTION (MGH Biorepository)**<br>**Instructions:** Blood samples will be collected in accordance with the policies and guidelines of the site's institution.<br><br>Please refer to section H of the site MOP for complete collection, processing, and shipping details.
Date Serum Samples Collected: _____ _____ / _____ _____ / _____ _____ _____ _____
Collector's Initials: _________________
Collection Number
Time Collected: ____ ____ : ____ ____ (24-Hr Clock)
Time centrifugation started: : ____ ____ : ____ ____ (24-Hr Clock)
Speed of centrifugation: _1300__ x gravity (g)
Duration of centrifugation: ___10____ minutes
Time aliquoted: ____ ____ : ____ ____ (24-Hr Clock)
Time aliquots put on dry ice: ____ ____ : ____ ____ (24-Hr Clock
Time aliquots put in -70ºC or -80ºC freezer: ____ ____ : ____ ____ (24-Hr Clock
Did serum remain pink after centrifugation, indicating hemolysis? ❍ Yes ❍ No

Number of full (0.5mL) aliquots: _______________

Volume of aliquots: **0.5mL**

Volume of LAST aliquot if less than 0.5mL: ________________

Subject Number: _7_ 1_ _3__ - ___ ___ ___	Subject GUID: _N__ _E_ _U ___ ___ ___ ___ ___ ___ ___ ___
Date: ___ ___/ ___ ___/ _2_ _0_ _1_ _8_	Evaluator Initials: ___ ___ ___
Study Visit: **x** Screening Visit	

PERMANENT ASSISTED VENTILATION (PAV)
Did the subject reach permanent assisted ventilation (PAV)* during the study?<br>☐ Yes ☐ No<br><br>If yes, start date: ___ ___ / ___ ___ / ___ ___ ___ ___<br><br>Comments: ______________________________
*Assisted ventilation is defined as permanent when BiPAP or invasive ventilation is used for > 22 hours in a 24 hour period for 7 consecutive days. Date started is the FIRST day of the 7 day period.
NON-INVASIVE VENTILATION (NIVV)
☐ Subject has not used non-invasive ventilation (NIV) during the study period.<br>☐ Non-invasive ventilation (NIV) use is continuing at the end of the study.

NIV Usage (Hours/Day)	Start Date	Stop Date

Subject Number: _7_ 1_ _3__ - ___ ___ ___	Subject GUID: _N__ _E__ _U ___ ___ ___ ___ ___ ___ ___ ___
Date: ___ ___/ ___ ___/ _2_ _0_ _1_ _8_	Evaluator Initials: ___ ___ ___
Study Visit: **x** Screening Visit	

FEEDING TUBE

Date recommended: ___ ___/___ ___/___ ___ ___ ___

Date accepted: ___ ___/___ ___/___ ___ ___ ___

Admission date: ___ ___/___ ___/___ ___ ___ ___

Discharge date: ___ ___/___ ___/___ ___ ___ ___

Type of Feeding Tube: ○ Nasogastric ○ Gastrostomy

Placement Method:

○ General surgery ○ Interventional radiology

○ Microscopic Laparotomy ○ Percutaneous Endoscopic Gastrostomy

○ Other, specify: ______________________________

Feeding tube was: ○ Prophylactic/Elective ○ Emergent

Morbidity/mortality related to feeding tube:

☐ Aspiration

☐ Death (please complete the Mortality Form)

☐ Excessive Pain

☐ Hemorrhage

☐ Local Infection

☐ Nausea/vomiting

☐ Oxygen desaturation/inadequate ventilation during procedure

☐ Peritonitis

☐ Procedure aborted secondary to anatomy

☐ Other, specify: ____

Subject Number: _7_ 1_ _3__ - ___ ___ ___	Subject GUID: _N__ _E__U ___ ___ ___ ___ ___ ___ ___ ___
Date: ___ ___/ ___ ___/ _2_ _0_ _1_ _8_	Evaluator Initials: ___ ___ ___
Study Visit: **x** Screening Visit	

**TRACHEOSTOMY**
Did the subject require a tracheostomy during the study?<br>☐ Yes<br>☐ No<br>Date recommended: ___ ___/___ ___/___ ___ ___ ___<br>Date of tracheotomy: ___ ___/___ ___/___ ___ ___ ___<br>Admission date: ___ ___/___ ___/___ ___ ___ ___<br>Discharge date: ___ ___/___ ___/___ ___ ___ ___<br>Reason for tracheotomy:<br>○ Respiratory Failure<br>○ Secretion Control<br>○ Other, specify: ______________________________

**DIAPHRAGM PACING SYSTEM (DPS)**
Did the subject have a diaphragm pacing system placed during the study?<br>☐ Yes ☐ No ☐ NA (placed prior to enrollment)<br>Admission Date: ____ ____/____ ____/____ ____ ____ ____ (MM/DD/YYYY)<br>Date of Placement: ____ ____/____ ____/____ ____ ____ ____ (MM/DD/YYYY)<br>Discharge Date: ____ ____/____ ____/____ ____ ____ ____ (MM/DD/YYYY)<br><br>Comments:<br>____<br>____<br>____<br>____________________

Answer ALS

Subject Number: _7_ 1_ _3__ - ___ ___ ___	Subject GUID: _N__ _E_ _U ___ ___ ___ ___ ___ ___ ___ ___
Date: ___ ___/ ___ ___/ _2_ _0_ _1_ _8_	Evaluator Initials: ___ ___ ___
Study Visit: **x** Screening Visit	

PREGNANCY
(To be completed for female subjects only)

Did the subject become pregnant during the study?: ☐ Yes ☐ No

Date reported:	____ ____/____ ____/____ ____ ____ ____
Start date of last menses:	____ ____/____ ____/____ ____ ____ ____
Date pregnancy confirmed:	____ ____/____ ____/____ ____ ____ ____
Anticipated date of childbirth:	____ ____/____ ____/____ ____ ____ ____

Pregnancy History:

	**0**	**1**	**2**	**3**	**4**	**5**	**6**	**> 6**
Number of Pregnancies	☐	☐	☐	☐	☐	☐	☐	☐
Number of Normal Deliveries	☐	☐	☐	☐	☐	☐	☐	☐
Spontaneous Miscarriage	☐	☐	☐	☐	☐	☐	☐	☐

Other (please specify):

Pregnancy Outcome:

☐ Not known at this date
☐ Still Birth
☐ Uneventful (normal/healthy baby)
☐ Neonatal death
☐ Induced Abortion
☐ Spontaneous Abortion
☐ Birth defects

Comments:____

Date of outcome: ___ ___/___ ___/___ ___ ___ ___

Pregnancy reported by (study staff name): ________________________________

Answer ALS

Subject Number: _7_ 1_ _3__ - ___ ___ ___	Subject GUID: _N__ _E_ _U ___ ___ ___ ___ ___ ___ ___ ___
Date: ___ ___/ ___ ___/ _2_ _0_ _1_ _8_	Evaluator Initials: ___ ___ ___
Study Visit: **x** Screening Visit	

SUBJECT FINAL DISPOSITION

Subject's participation in this study has ended.

☐ Yes

☐ No

If **Yes**, please select **one** of the following options:

☐ Subject was a Screen Failure	Date of Screen Failure:
	Reason:

☐ Subject died (Please complete Mortality Form)

☐ Discontinued Participation

If Discontinued Participation, Date Last Known Alive: ___ ___ / ___ ___ / ___ ___ ___ ___

☐ Other (Specify): ____

If Other, Date Last Known Alive: ___ ___ / ___ ___ / ___ ___ ___ ___

MORTALITY FORM

Did the subject die? ☐ Yes ☐ No

If **Yes**:

Date of death: ____ ____/____ ____/____ ____ ____ ____ (MM/DD/YYYY)

Cause of death:

____

ICD-10 CM Code for cause of death: __________________________________

Was a general autopsy performed? ☐ Yes ☐ No

If **Yes**, Date of Autopsy: ____ ____/____ ____/____ ____ ____ ____ (MM/DD/YYYY)

If **YES**, location of autopsy: ____

If **Yes,** has a copy of the autopsy report been obtained? ☐ Yes ☐ No
