# Supplementary material for: Answer ALS, a large-scale resource for sporadic and familial ALS combining clinical and multi-omics data from induced pluripotent cell lines
Source: Nat Neurosci. 2022 Feb 3;25(2):226–37. doi: 10.1038/s41593-021-01006-0 (PMC8825283; doi:10.1038/s41593-021-01006-0)
Supplement: Supplementary file 2 — Supplementary Table 1 Clinic locations for AALS. Supplementary Table 2 AALS clinical events. Supplementary Table 3 Data and biospecimen sources. The authors declare that all data supporting the findings of the present study are available within the paper and its supplementary information files and web portals listed in this table. Supplementary Table 4 Overall clinical demographics. Supplementary Table 5 Fast versus slow progression demographics. Supplementary Table 6 Characterization and validation of IPS cell lines. SupplementaryTable 19 Cell line authentication. Supplementary Table 20 Data-level definitions. Supplementary Table 21 Examples of data levels for each assay. Supplementary Table 22 Stage 1 cell culture media. Supplementary Table 23 Stage 2 platedown media. Supplementary Table 24 Stage 2 media. Supplementary Table 25 Stage 3 media. Supplementary Table 26 Antibody reagents for iPS evaluation. [file 41593_2021_1006_MOESM2_ESM.pdf]

Supplementary Tables 1-6, 19-26

Supplementary Table 1. Clinic Locations for Answer ALS

Clinic	Location	Local PI
Johns Hopkins University	Baltimore, MD	Nick Maragakis MD
Massachusetts General Hospital	Boston, MA	James Berry MD
Emory University	Atlanta, GA	Jon Glass MD
Washington University	St. Louis, MO	Tim Miller MD, PhD
Northwestern University	Chicago, IL	Senda Ajroud-Driss MD
Texas Neurology	Dallas, TX	Daragh Heitzman MD
Cedars Sinai Medical Center	Los Angeles, CA	Robert Baloh MD, PhD

Supplementary Table 2. Answer ALS Clinical Events

	Enrollment	Follow-up Period				Post Participation Followup Period[1]
Week	0	12	24	36	48	Every 3 months
Window		84±42 days	168±42 days	252±42 days	336±42 days	(±42 days)
Visit Number	Screening/Visit 1	Visit 2	Visit 3	Visit 4	Visit 5	Follow-up
Eligibility/Consenting						
Written informed consent	X[HC]					
Inclusion/Exclusion review	X[HC]					
Clinical Information Collection						
Medical History/Demographics*	X[HC]					
Create GUID*	X[HC]					
ALS History/Genetics*	X					
Environmental Questionnaire*	X[HC]					
Concomitant Medication review*	X[HC]	X[HC]	X	=	X	
Adverse Event Review*	X[HC]	X[HC]	X	X	X	
Key Study Event Review	X	X	X	X	X	X
Vitals Signs/Height&Weight[2]	X[HC]	X[HC]	X	X	X	
Confirmation of Diagnosis*	X[HC]	X[HC]	X	X	X	X
Outcome Measure Collection						
Slow vital capacity	X	X	X	X	X	X
Hand Held Dynamometry	X[HC**]	X[HC**]	X	X	X	
Penn Upper Motor Neuron Burden (UMN-B)[3]	X[HC]	X	X	X	X	
ALSFRS-R*	X	X	X	X	X	X
ALS Cognitive Behavioral Scale (ALSCBS)	X	X	X	X	X	
SAMPLE COLLECTION						
Lumbar Puncture (CSF collection) OPTIONAL	X[HC]	X[HC]	X	X	X	
Additional Blood Samples OPTIONAL	X[HC]	X[HC]	X	X	X	
Biomarker Studies	X[HC]	X[HC]	X	X	X	

[1] Follow-up continued every 3 months to collect data on survival status/Key Study Events. This information was obtained via chart review and/or participant contact.
[2] Vital signs include systolic and diastolic pressure in mmHg, respiratory rate/minute, heart rate/minute, temperature and weight. Height measured at Screening Visit only.
[3] The Penn Upper Motor Neuron-Burden (UMN-B) includes testing of reflexes, the CNS Lability Scale (CNS-LS) and the Ashworth Spasticity Scale
[4] The participant may choose to have CSF collected at any visit (if only collected once), all visits, or no visits.
[5] Additional blood samples include Uric Acid, Creatinine, Phosphorus, and Creatine Kinase (CK).
[6] Biomarker Studies included blood samples for plasma, serum, whole blood for DNA and aliquots, and PBMC collection. PBMC was collected at Visit I. Whole blood for DNA sequencing was drawn once at any visit, preferably Visit 1. Whole blood can be drawn at any visit for aliquot. Plasma and serum were collected at each visit. Plasma and serum were stored in the NEALS Biorepository for future analysis. Please refer to the Site Manual of Procedures (MOP) for collection, storage, and shipping information.
[HC]Procedures to be completed also by healthy control volunteers,
[HC**] HHD is optional for healthy control (!·IC) volunteers only
* Procedures with an asterisk(*) were completed via the telephone if these measures were not collected at the scheduled visits.

Supplementary Table 3. Data and Biospecimen Sources. The authors declare that all data supporting the findings of this study are available within the paper and its supplementary information files and web portals listed in this table.

**Reagent**	**Location**	**Web Address**
Clinical Data sets	Neurobank	http://www.neurobank.org
Whole Genome	NY Genome Center	https://www.nygenome.org/als-consortium/
Induced Pluripotent cell lines	Cedars Sinai	https://www.cedars-sinai.edu/Research/Research-Cores/Induced-Pluripotent-Stem-Cell-Core-/Answer-ALS-Project.aspx
Biofluids (serum, CSF)	Neurobank	https://www.neals.org/for-als-researchers/neals-sample-repository/
ALL Clinical and Omics data (transcriptome, proteome, epigenome); selecting/ ordering cell lines	Answer ALS	https://data.answerals.org/home

Supplementary Table 4. Overall Clinical Demographics

			Subjects			
**Variable**	**Level**	**Overall**	**ALS**	**Asymptomatic ALS**	**Healthy Control**	**Non-ALS-MND**
Participants	N	100.0% (1047)	82.2% (861)	1.1% (12)	10.3% (108)	6.3% (66)
Sex	Female	40.6% (423)	37.4% (320)	58.3% (7)	66.4% (71)	37.9% (25)
	Male	59.4% (618)	62.6% (536)	41.7% (5)	33.6% (36)	62.1% (41)
	[missing]	.% (6)	.% (5)	.% (0)	.% (1)	.% (0)
Race	American Indian	0.2% (2)	0.1% (1)	0.0% (0)	1.0% (1)	0.0% (0)
	Asian	2.0% (21)	1.5% (13)	0.0% (0)	5.7% (6)	3.0% (2)
	Black	4.8% (49)	5.0% (42)	0.0% (0)	4.8% (5)	3.0% (2)
	Pacific Islander	0.1% (1)	0.1% (1)	0.0% (0)	0.0% (0)	0.0% (0)
	White	92.9% (956)	93.3% (789)	100.0% (12)	88.6% (93)	93.9% (62)
	[missing]	.% (18)	.% (15)	.% (0)	.% (3)	.% (0)
Ethnicity	Hispanic or Latino	4.8% (50)	5.3% (45)	0.0% (0)	2.8% (3)	3.1% (2)
	Not Hispanic or Latino	95.2% (989)	94.7% (810)	100.0% (12)	97.2% (104)	96.9% (63)
	[missing]	.% (8)	.% (6)	.% (0)	.% (1)	.% (1)
Age at baseline (yrs)	Years (mean(SD))	58.9±11.6 (20.0,91.0)	59.3±11.1 (24.0,91.0)	48.3±10.3 (33.0,62.0)	55.0±14.1 (20.0,82.0)	61.9±12.0 (26.0,85.0)
Time between symptom onset and diagnosis	Months (mean(SD))	15.9±20.4 (-5.7,286)	14.8±16.8 (-5.7,185)	N/A	N/A	40.8±52.6 (0.1,286)
Time between symptom onset and study enrollment	Months (mean(SD))	32.0±39.4 (0.6,458)	29.8±35.6 (0.6,458)	N/A	N/A	78.4±75.3 (11.1,353)
BMI at screening visit	mean(SD)	26.8±6.39 (10.1,150)	26.5±4.83 (10.1,44.4)	29.2±3.38 (23.6,34.2)	29.2±14.9 (17.0,150)	27.3±5.61 (16.6,47.3)
ALSFRS-R at first ALSFRS-R visit	mean(SD)	33.8±8.65 (0.0,47.0)	33.8±8.67 (0.0,47.0)	N/A	N/A	33.5±8.44 (7.0,46.0)
ALSFRS-R slope		-.73±0.87 (-5.1,1.4)	-.77±0.88 (-5.1,1.4)	N/A	N/A	-.11±0.40 (-1.6,1.0)
FVC (%-pred) at first ALSFRS-R visit	mean(SD)	69.9±24.0 (4.0,126)	69.6±23.9 (4.0,125)	N/A	N/A	73.7±25.3 (17.0,126)
FVC slope		-1.5±2.53 (-16,14.1)	-1.6±2.59 (-16,14.1)	N/A	N/A	-.12±0.86 (-1.9,2.1)
Edaravone	No	90.0% (942)	87.9% (757)	100.0% (12)	100.0% (108)	98.5% (65)
	Yes	10.0% (105)	12.1% (104)	0.0% (0)	0.0% (0)	1.5% (1)
Riluzole	No	46.3% (485)	36.4% (313)	91.7% (11)	100.0% (108)	80.3% (53)
	Yes	53.7% (562)	63.6% (548)	8.3% (1)	0.0% (0)	19.7% (13)
Follow-Up Duration	Months (mean(SD))	13.3±17.3 (0.0,340)	12.5±12.6 (0.0,94.1)	N/A	N/A	24.0±47.2 (0.0,340)

Supplementary Table 5. Fast vs Slow Progression Demographics

Fast vs. Slow Progressors			Cohort		Level
Variable	Level	Overall	Fast	Slow	P-val
Participants	N	100.0% (200)	50.0% (100)	50.0% (100)	.
Sex	Female	38.0% (76)	42.0% (42)	34.0% (34)	0.244
	Male	62.0% (124)	58.0% (58)	66.0% (66)	0.244
	[missing]	N/A	N/A	N/A	N/A
Race	American Indian	N/A	N/A	N/A	.
	Asian	0.5% (1)	0.0% (0)	1.0% (1)	0.321
	Black	4.5% (9)	2.0% (2)	7.0% (7)	0.094
	Pacific Islander	0.5% (1)	0.0% (0)	1.0% (1)	0.321
	White	94.4% (187)	98.0% (96)	91.0% (91)	0.033
	[missing]	.% (2)	.% (2)	.% (0)	N/A
Ethnicity	Hispanic or Latino	4.0% (8)	5.1% (5)	3.0% (3)	0.462
	Not Hispanic or Latino	96.0% (191)	94.9% (94)	97.0% (97)	0.462
	[missing]	.% (1)	.% (1)	.% (0)	N/A
Age at baseline (yrs)	Years (mean(SD))	60.6±10.4 (29.0,85.0)	60.5±10.2 (29.0,84.0)	60.8±10.8 (34.0,85.0)	0.855
Time between symptom onset and diagnosis	Months (mean(SD))	16.5±21.0 (-5.7,157)	10.3±11.0 (-5.7,90.3)	23.1±26.6 (1.1,157)	<.001
Time between symptom onset and study enrollment	Months (mean(SD))	33.9±48.9 (0.6,458)	16.9±15.7 (0.6,104)	52.0±63.7 (2.9,458)	<.001
BMI at screening visit	mean(SD)	26.0±4.90 (13.7,42.0)	25.6±4.83 (13.7,38.1)	26.5±4.97 (16.0,42.0)	0.218
ALSFRS-R at first ALSFRS-R visit	mean(SD)	32.9±8.20 (10.0,47.0)	33.8±6.93 (14.0,46.0)	32.0±9.24 (10.0,47.0)	0.111
ALSFRS-R slope		-1.1±1.44 (-5.1,1.4)	-2.3±0.78 (-5.1,-1.6)	0.25±0.35 (-0.0,1.4)	<.001
FVC (%-pred) at first ALSFRS-R visit	mean(SD)	66.8±21.8 (12.0,100)	65.4±21.1 (16.0,94.0)	68.4±22.7 (12.0,100)	0.528
FVC slope		-1.9±2.66 (-16,3.0)	-3.3±2.98 (-16,3.0)	-.76±1.68 (-6.5,1.7)	<.001
Edaravone	No	87.5% (175)	85.0% (85)	90.0% (90)	0.285
	Yes	12.5% (25)	15.0% (15)	10.0% (10)	
Riluzole	No	36.5% (73)	32.0% (32)	41.0% (41)	0.186
	Yes	63.5% (127)	68.0% (68)	59.0% (59)	
Follow-Up Duration	Months (mean(SD))	11.3±11.8 (1.3,94.1)	7.86±4.36 (1.3,18.6)	14.7±15.4 (1.9,94.1)	<.001
Subject Death	No	52.0% (104)	28.0% (28)	76.0% (76)	<.001
	Yes	48.0% (96)	72.0% (72)	24.0% (24)	
Time Onset to Death (months)	Months (mean(SD))	34.7±27.6 (8.3,187)	29.5±15.7 (8.3,105)	50.3±45.3 (9.7,187)	0.001

Supplementary Table 6. Characterization and Validation of IPS cell lines

The following testing specifications have been met for the specified cell line:

Test Description	Test Specification	Result
**Mycoplasma**	No contamination detected	Pass
**Alkaline Phosphatase Staining**	Positive AP staining	Pass
**Karyotype by G-Banding**	Normal Karyotype	Pass
**Pluripotency**		
PrimeView Global Gene Expression Profile Assay (PluriTest)	Pluripotency score ≥ 20 and novelty score ≤ 1.6	N/A
Immunocytochemistry (IF-IC)	OCT3/4, NANOG, SOX2, TRA-1-60, TRA-1-81, SSEA4	Pass
TaqMan® hPSC Scorecard™ Assay	Confirm appropriate expression of self-renewal factors	Pass
**Differentiation**		
EB Formation	Successful Embryoid Body (EB) formation after 14 days	Pass
TaqMan® hPSC Scorecard™ Assay	Tri-lineage differentiation potential *Endoderm, Ectoderm and Mesoderm*	Pass
**Reprogramming Plasmid Integration**		
Genomic DNA PCR	Confirm the presence or absence of exogenous reprogramming plasmids	Absent
**Parent Cell Line Lineage Determination**		
TCRB + TCRG T-Cell Clonality Assay (Blood derived cell lines only)	Confirm presence or absence of clonal T-cell receptor beta chain and gamma chain gene rearrangements in iPSCs	Confirmed
**Cell Line Authentication**		
STR Analysis	Confirm identity matching score is above 80%	Confirmed

Supplemental Table 19. Cell Line Authentication
A typical example of an iPSC line STR matching report

CELL LINE AUTHENTICATION:

Parent Cell Line: NEUFV237VCZ

AMEL	CSF1PO	D13S317	D16S539	D5S818	D7S820	TH01	TPOX	vWA
X, Y	10	11, 12	11	11	9, 10	9.3	8, 11	19

iPSC Line: CS7VCZiALS-n3

AMEL	CSF1PO	D13S317	D16S539	D5S818	D7S820	TH01	TPOX	vWA
X, Y	10	11, 12	11	11	9, 10	9.3	8, 11	19

% Identity Match: 100%

IDEXX IBR #(s): 22009-2017 #19

Supplemental Table 20. Data Level Definitions

Data Levels	Description
Level 1	Immutable raw data
Level 2	Raw data mapped to reference
Level 3	Most-processed sample-specific data
Level 4	Analysis from joining Level 3 data for specific assay

Supplementary Table 21. Examples of Data Levels for each assay

**Assay**	**Level 1**	**Level 2**	**Level 3**	**Level 4**
Genomics	FASTQ	BAM	VCF	Joint VCF
Epigenomics	FASTQ	BAM	Peaks	Differential Peaks
Transcriptomics	FASTQ	BAM	Counts	Differential Counts
Proteomics	WIFF	MZML	Intensities	Differential Intensities

Supplementary Table 22. Stage 1 Cell Culture Media

Stage 1 (Day 0-Day 6)	Manufacturer	Catalog #	1X Concentration
IMDM	Life Technologies	12440061	47.5%
F12	Life Technologies	11765062	47.5%
NEAA	Life Technologies	11140-50	1%
B27	Life Technologies	17504044	2%
N2	Life Technologies	17502048	1%
Anti/Anti	Life Technologies	15240062	1%
LDN193189	Cayman Chemical	19396	0.2 µM
CHIR99021	Xcess bioscience	M60002	3 µM
SB431542	Cayman Chemical	13031	10 µM

Supplementary Table 23. Stage 2 Platedown Media

Stage 2 Platedown (Day 6)	Manufacturer	Catalog #	1X Concentration
IMDM	Life Technologies	12440061	47.45%
F12	Life Technologies	11765062	47.45%
NEAA	Life Technologies	11140-50	1%
B27	Life Technologies	17504044	2%
N2	Life Technologies	17502048	1%
Anti/Anti	Life Technologies	15240062	1%
All-trans RA	Stemgent	04-0021	0.1 µM
SAG	Cayman Chemical	11914	1 µM
LDN193189	Cayman Chemical	19396	0.2 µM
CHIR99021	Xcess bioscience	M60002	3 µM
SB431542	Cayman Chemical	13031	10 µM
Rock Inhibitor (Y-27632)	Stemcell Technologies	72308	10 µM

Supplementary Table 24. Stage 2 Media

Stage 2 (Day 7-11)	Manufacturer	Catalog #	1X Concentration
IMDM	Life Technologies	12440061	47.5%
F12	Life Technologies	11765062	47.5%
NEAA	Life Technologies	11140-50	1%
B27	Life Technologies	17504044	2%
N2	Life Technologies	17502048	1%
Anti/Anti	Life Technologies	15240062	1%
All-trans RA	Stemgent	04-0021	0.1 µM
SAG	Cayman Chemical	11914	1 µM
LDN193189	Cayman Chemical	19396	0.2 µM
CHIR99021	Xcess bioscience	M60002	3 µM
SB431542	Cayman Chemical	13031	10 µM

Supplementary Table 25. Stage 3 Media

Stage 3 (Day 12-Day32)	Manufacturer	Catalog #	1X Concentration
IMDM	Life Technologies	12440061	47.5%
F12	Life Technologies	11765062	47.5%
NEAA	Life Technologies	11140-50	1%
B27	Life Technologies	17504044	2%
N2	Life Technologies	17502048	1%
Anti/Anti	Life Technologies	15240062	1%
*SAG	Cayman Chemical	11914	0.1 µM
db-cAMP	Millipore	28745	0.1 µM
All-trans RA	Stemgent	04-0021	0.5 µM
Compound E	Calbiochem	565790	0.1 µM
DAPT	Cayman Chemical	13197	2.5 µM
Ascorbic Acid	Sigma-Aldrich	A4403	200 ng/mL
BDNF (-80)	Peprotech	450-02	10 ng/mL
GDNF (-80)	Peprotech	450-10	10 ng/mL

Supplementary Table 26. Antibody reagents for iPS Evaluation

Antibody or Stain	Manufacturer	Catalog #	Dilution
SMI 32	Biolegend	801701	1:1000
Human Islet-1 Antibody	R&D	AF1837	1:250
Nkx-6.1	DSHB	F55A10-s	1:1000
Tuj (TUBB3 polyclonal antibody)	Abnova	PAB7874	1:1000
Anti-Nestin, Human Antibody	Sigma Aldrich	ABD69	1:1000
S100beta	Sigma Aldrich	S2532	1:250
Donkey anti-mouse secondary	Life Technologies	A-10037	1:1000
Donkey anti-rabbit secondary	Life Technologies	A-31573	1:1000
Donkey anti-goat secondary	Life Technologies	A-21447	1:1000
DAPI	Life Technologies	D3571	0.1µg/mL
