# Supplementary material for: Answer ALS, a large-scale resource for sporadic and familial ALS combining clinical and multi-omics data from induced pluripotent cell lines
Source: Nat Neurosci. 2022 Feb 3;25(2):226–37. doi: 10.1038/s41593-021-01006-0 (PMC8825283; doi:10.1038/s41593-021-01006-0)
Supplement: Supplementary file 3 — Reporting Summary [file 41593_2021_1006_MOESM3_ESM.pdf]

nature research

Corresponding author(s): Jeffrey D. Rothstein MD, PhD

Last updated by author(s): May 20, 2021

Reporting Summary

Nature Research wishes to improve the reproducibility of the work that we publish. This form provides structure for consistency and transparency in reporting. For further information on Nature Research policies, see our Editorial Policies and the Editorial Policy Checklist.

Statistics

For all statistical analyses, confirm that the following items are present in the figure legend, table legend, main text, or Methods section.

n/a	Confirmed	
☐	☒	The exact sample size (*n*) for each experimental group/condition, given as a discrete number and unit of measurement
☐	☒	A statement on whether measurements were taken from distinct samples or whether the same sample was measured repeatedly
☐	☒	The statistical test(s) used AND whether they are one- or two-sided<br>*Only common tests should be described solely by name; describe more complex techniques in the Methods section.*
☐	☒	A description of all covariates tested
☐	☒	A description of any assumptions or corrections, such as tests of normality and adjustment for multiple comparisons
☐	☒	A full description of the statistical parameters including central tendency (e.g. means) or other basic estimates (e.g. regression coefficient) AND variation (e.g. standard deviation) or associated estimates of uncertainty (e.g. confidence intervals)
☐	☒	For null hypothesis testing, the test statistic (e.g. *F*, *t*, *r*) with confidence intervals, effect sizes, degrees of freedom and *P* value noted<br>*Give P values as exact values whenever suitable.*
☒	☐	For Bayesian analysis, information on the choice of priors and Markov chain Monte Carlo settings
☒	☐	For hierarchical and complex designs, identification of the appropriate level for tests and full reporting of outcomes
☐	☒	Estimates of effect sizes (e.g. Cohen's *d*, Pearson's *r*), indicating how they were calculated

Our web collection on statistics for biologists contains articles on many of the points above.

Software and code

Policy information about availability of computer code

Data collection

NeuroBank Web based clinical data collection; Neurobank is a proprietary Massachusetts General Hospital owned software

Data analysis

IBM Watson employed proprietary Voice and cognition Analytics software.
Encode blacklist (v2)
Homer (v4.11)
Simple Error Rate Estimate (SERE)
Sentieon v. 201911
SIFT9
PolyPhen210
Mutation Taster11
Intervar Li
Annovar v. 2018Apr16
tRVIS13
Gene Damage Index (GDI)
LoFTool
Python library scikit-allel package
LRT_Prediction
Mutation Taster
Mutation assessor
FATHMM prediction
dbNSFP (RadialSVM_pred and LR_pred)
RegulomeDB
Hisat2

April 2020

QCed
featureCounts
DESeq2
Ingenuity Pathway Analysis
GSEA
GOrilla
Cytoscape
Bowtie2
MACS2
DESeq2
PyProphet
mapDIA

For manuscripts utilizing custom algorithms or software that are central to the research but not yet described in published literature, software must be made available to editors and reviewers. We strongly encourage code deposition in a community repository (e.g. GitHub). See the Nature Research guidelines for submitting code & software for further information.

Data

Policy information about availability of data

All manuscripts must include a data availability statement. This statement should provide the following information, where applicable:

- Accession codes, unique identifiers, or web links for publicly available datasets
- A list of figures that have associated raw data
- A description of any restrictions on data availability

All data supporting the findings of this study are available within the paper and its supplementary information files and web portals listed in Extended Table 3. The public data portal is: https://dataportal.answerals.org/home. All data are supplied as raw data files. Figures summarize the totality of the data acquired to date. All data set are periodically updated on the publicly accessible web portal. Data is freely available to all users after completing an online Data Use Agreement.

Field-specific reporting

Please select the one below that is the best fit for your research. If you are not sure, read the appropriate sections before making your selection.

☒ Life sciences ☐ Behavioural & social sciences ☐ Ecological, evolutionary & environmental sciences

For a reference copy of the document with all sections, see nature.com/documents/nr-reporting-summary-flat.pdf

Life sciences study design

All studies must disclose on these points even when the disclosure is negative.

Sample size

The total population of the resource was approximately ~1100 ALS patients and controls. As this was not a clinical trial there was no predetermined sample size. The overall plan was to gather approximately 50-100 patient with the C9orf72 mutation out of the total enrolled sample. Since the C9orf72 patient population is estimated, in the USA, to be 8% of sporadic ALS and 20-40% of familial ALS cases, the guesstimate was the plan to gather approx 1000 patients across the USA would achieve that goal.

Data exclusions

no data exclusions

Replication

Batch technical control (BTC): The BTC controls for technical variability of a particular 'Omics assay between different batch runs. Briefly, one iPSC line from a healthy donor (CS2AE8iCTR-n6 line) was differentiated in a single large batch at the beginning of the project at the cell generation center (Cedars-Sinai iPSC Core). Multiple biological samples, including snap frozen cell pellets and cryopreserved cell pellets, were prepared to last over a significant period of the data generation component of the project. With each shipment batch, end users at each 'Omics center receive the appropriate BTC biological sample. Each shipment batch comprises three to four batches of iPSC-derived motor neurons of ALS and healthy control (CTR) subjects, as well as the BTC biological sample, while each differentiation batch comprises 10-15 iPSC lines from different experimental subjects. Since BTC pellets were produced at same time with the same diMNs differentiation standard operating procedure (SOP), a given assay should technically return similar results for any BTC sample across multiple 'Omics batch runs. The BTC thus controls for 'Omics assay-specific variability.
Batch differentiation control (BDC): The BDC controls for inter-batch variability in iPSC differentiation to diMNs. Briefly, a differentiation batch comprises 10-15 iPSC lines from different ALS and CTR subjects. The same iPSC line used to produce the BTC (CS2AE8iCTR-n6 line) is differentiated in every batch with the other experimental iPSC lines at the cell generation center and is referred to as the Batch Differentiation Control (BDC). This line is thawed, expanded, differentiated, and pelleted in addition to the ALS or healthy control (CTR) lines in each batch. The repeated differentiation of this single line, therefore, serves as a differentiation control, reflecting the intrinsic variability in the iPSC to diMNs differentiation process of the same line across multiple differentiation batches. 'Omics centers receive a BDC sample along with ALS and CTR samples for each differentiation batch. Thus, in addition to the BTC sample, a shipment to the 'Omics center contains multiple BDC samples (one for each differentiation batch included in the shipment).
All replication studies were successful.

Randomization

The program was a resource generation program so no patient randomization was appropriate

Blinding

The program was a resource generation program, and not a clinical trial, so no patient blinding was appropriate

Reporting for specific materials, systems and methods

We require information from authors about some types of materials, experimental systems and methods used in many studies. Here, indicate whether each material, system or method listed is relevant to your study. If you are not sure if a list item applies to your research, read the appropriate section before selecting a response.

Materials & experimental systems

n/a	Involved in the study	
☐	☒	Antibodies
☐	☒	Eukaryotic cell lines
☒	☐	Palaeontology and archaeology
☒	☐	Animals and other organisms
☐	☒	Human research participants
☐	☒	Clinical data
☒	☐	Dual use research of concern

Methods

n/a	Involved in the study	
☒	☐	ChIP-seq
☒	☐	Flow cytometry
☒	☐	MRI-based neuroimaging

Antibodies

Antibodies used

SMI 32, Biolegend 801701, 1:1000
Human Islet-1 Antibody, R&D AF1837, 1:250
Nkx-6.1 ,DSHB F55A10-s, 1:1000
Tuj (TUBB3 polyclonal antibody), Abnova PAB7874, 1:1000
Anti-Nestin, Human Antibody, Sigma Aldrich ABD69 ,1:1000
S100beta Sigma Aldrich S2532 1:250
Donkey anti-mouse secondary Life Technologies A-10037 1:1000
Donkey anti-rabbit secondary Life Technologies A-31573 1:1000
Donkey anti-goat secondary Life Technologies A-21447 1:1000
DAPI Life Technologies D3571 0.1µg/mL

Validation

All commercial antibodies deteiled provide validation information online

Eukaryotic cell lines

Policy information about cell lines

Cell line source(s)

Answer ALS generates iPS cell lines from ALS and control subject PBMC as detailed in the manuscript; HEK293 cell line (MilliporeSigma, 85120602-1VL).

Authentication

1. IPS Cell Lines:
Extended Data Table 6 outlines all the procedure employed to characterize and validate the IPS cell lines generated including:Mycoplasma Detection
Alkaline Phosphatase Staining
Karyotype by G-Banding
Immunocytochemistry (IF-IC)
TaqMan® hPSC Scorecard™ Assay
EB Formation
Genomic DNA PCR
TCRB + TCRG T-Cell Clonality Assay
STR Analysis

2. HEK293 Cell line.
HEK293 cell line was not authenticated (commercial source)

Mycoplasma contamination

All cell lines undergo mycoplasma detection and were negative

Commonly misidentified lines
(See ICLAC register)

none

Human research participants

Policy information about studies involving human research participants

Population characteristics

The enrolled participant population for the Answer ALS program (1047 individuals) had clinical characteristics comparable to past large sALS population demographics, with a slightly higher number of male (618) than female (423) participants, site of disease onset predominantly limb rather than bulbar, and a mean age of disease onset of approximately 57. The mean delay in clinical diagnosis for ALS patients included in the study was 14.8 months. A higher percentage of patients with rapid

progression had bulbar onset disease. There was a wide range of disease progression rates over the time period of observation with an average follow-up duration of 12.5 months and an average rate of decline of 0.77 points per month. The smaller population of fALS patients in the resource had typical representations of the common gene mutations including C9orf72 and SOD1, with a small subset of C9orf72 and non C9orf72 ALS patients developing cognitive decline during the study. A small number of individuals were ALS mutation carriers (Asymptomatic ALS, 12) without overt neurological disease. Non-ALS MND included patients with predominantly upper motor neuron disease and not formally categorized as ALS (e.g. primary lateral sclerosis)(66). The healthy control subject population (108) consisted of age matched participants without ALS or a family history of ALS.

Recruitment

Local ALS Clinic population (Maryland, Massachusetts, Ohio, Georgia, Texas, Illinois, California, Missouri). The were no recruitment bias; all patients diagnosed with ALS ALS were eligible to join study.

Ethics oversight

IRB review boards at Johns Hopkins, Massachusetts General Hospital, Ohio State University, Emory University, Northwestern University, Neurology Center, and Cedar Sinai Medical Center approved the clinical recruitment and, data and biological collection protocol at each of the 8 clinical research sites

Note that full information on the approval of the study protocol must also be provided in the manuscript.

Clinical data

Policy information about clinical studies

All manuscripts should comply with the ICMJE guidelines for publication of clinical research and a completed CONSORT checklist must be included with all submissions.

Clinical trial registration

NCT02574390

Study protocol

Patients will have 5 study visits; screening, 3, 6, 9 and 12 months. There will be a one year post-participation follow-up period, during which they will receive an email or phone call interview once every 3 months. During the first year, samples will be collected, breathing, muscle strength, spasticity, general function and cognitive behavior will be assessed. Healthy controls will have 2 study visits during which blood samples will be collected and questionnaires given.
Study Population
patients with ALS, Primary Lateral Sclerosis Flail Arm ALS Progressive Muscular Atrophy Monomelic Amyotrophy Motor Neuron Disease Asymptomatic ALS Gene Carriers
Criteria
Inclusion Criteria:
Participants with familial or sporadic ALS diagnosed as possible, laboratory-supported probable, probable or definite according to the World Federation of Neurology (WFN) El Escorial criteria, Primary Lateral Sclerosis Flail Arm ALS, Progressive Muscular Atrophy, Monomelic Amyotrophy, Motor Neuron Disease, Asymptomatic ALS Gene Carriers
Participants who are ages 18-100, inclusive.
Exclusion Criteria:
Participants with Spinal-Bulbar Muscular Atrophy
Known diagnosis of HIV/AIDS, Hepatitis B, or Hepatitis C.

Primary Outcome Measures :
ALS Functional Rating Scale-Revised (ALSFRS-R) [Time Frame: once every 3 months for one year]
12 questions about patient's ability to function in certain activities of daily living. Each question is out of 4 with 4 being normal and 0 being completely impaired.

ALS Cognitive Behavioral Scale (ALS-CBS) [Time Frame: once every 3 months for one year]
short measure of cognition and behavior in patients with ALS. The cognitive portion consists of 8 tasks with a perfect score being 20. The behavioral portion measures changes in personality and behavior since the onset of ALS symptoms as well as mood, pseudobulbar affect and fatigue and is completed by a family member or caregiver. A normal score is 45.

Slow Vital Capacity (SVC) [Time Frame: once every 3 months for one year]
measurement of the maximum amount of air that can be exhaled following a deep breath.

Strength Testing with Hand Held Dynamometer (HHD) [Time Frame: once every 3 months for one year]
muscle strength testing performed on upper and lower limbs, ankles, wrists and fingers using a small hand held device. These measurements are followed over time and compared to measure decline.

Biospecimen Retention: Samples With DNA
plasma, serum, DNA, Cerebrospinal fluid, induced pluripotent stem cells,

Data collection

Neurobank electronic data forms. Data was collected at each patient visit at the study site clinics (Johns Hopkins University, Baltimore MD; Emory University, Atlanta, GA; Massachusetts General Hospital, Boston MA, Cedars Sinai, Los Angeles; Northwestern Univ, Chicago IL; Ohio State Univ, Columbus OH; Neurology Center, Dallas, TX

Outcomes

Standard ALS outcome measures - ALSFRS, CBS, neurological exam, survival; forced vital capacity; Population statistics listed
